# α‐Functionalisation of Ketones Through Metal‐Free Electrophilic Activation

**DOI:** 10.1002/anie.202006398

**Published:** 2020-09-11

**Authors:** Wojciech Zawodny, Christopher J. Teskey, Magdalena Mishevska, Martin Völkl, Boris Maryasin, Leticia González, Nuno Maulide

**Affiliations:** ^1^ Institute of Organic Chemistry University of Vienna Währinger Strasse 38 1090 Vienna Austria; ^2^ Institute of Theoretical Chemistry University of Vienna Währinger Strasse 17 1090 Vienna Austria

**Keywords:** sigmatropic rearrangement, vinyl cations, vinyl triflate, α-arylation, α-oxyamination

## Abstract

Triflic anhydride mediated activation of acetophenones leads to highly electrophilic intermediates that can undergo a variety of transformations when treated with nucleophiles. This electrophilic ketone activation gives access to α‐arylated and α‐oxyaminated acetophenones under metal‐free conditions in moderate to excellent yields and enables extension to the synthesis of arylated morpholines via generation of vinylsulfonium salts. Computational investigations confirmed the transient existence of intermediates derived from vinyl triflates and the role of the oxygen atoms at the *para* position of aromatic ring in facilitating their stabilisation.

## Graphical abstract

Electrophilic ketone activation: Triflic anhydride mediated activation of acetophenones leads to highly electrophilic intermediates undergoing various transformations with nucleophiles. The methodology gives access to α‐arylated and α‐oxyaminated acetophenones under metal‐free conditions in moderate to excellent yields.

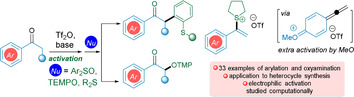

Functionalisation of carbonyl compounds at the α‐position is of central importance in synthetic organic chemistry. The α‐arylation of carbonyl compounds has gained particular attention due to the interesting pharmacological and biological properties exhibited by such scaffolds.[^1a^, ^1b^] The advent of transition‐metal‐catalysed coupling reactions (involving mainly Pd, Ni and Cu) of aryl halides with carbonyl‐derived enolates has facilitated access to them (Scheme 1 a).[^1c^, ^1d^, ^1e^, ^1f^] Transition‐metal‐free approaches to α‐arylation involve applications of stoichiometric processes involving enolate anions and electrophilic aromatic derivatives of iodine,^[2]^ sulfur,^[3]^or benzyne.^[4]^ Methodologies proceeding via *N*‐alkoxyenamines^[5]^ or enolonium equivalents^[6]^ have also been developed.

**Scheme 1:**
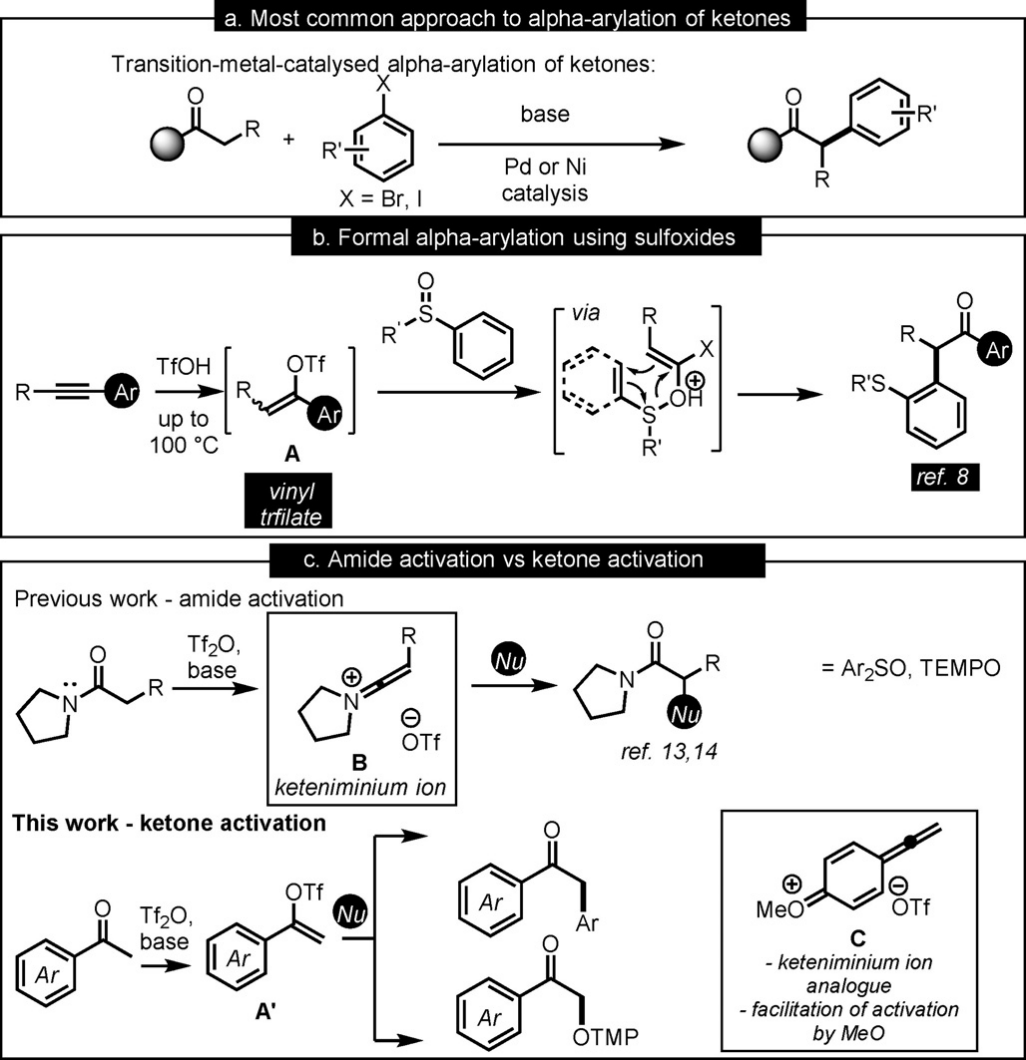
(a) Previous approaches to α‐arylation of ketones. (b) Hydrative α‐arylation of acetylenes. (c) Amide activation and ketone activation.

Radical nucleophilic aromatic substitution (S_RN_1) also enables α‐arylation of ketones.^[7]^ Alternatively, a formal metal‐free α‐arylation of carbonyl compounds can be achieved by using aryl sulfoxides (Scheme 1 b).^[8]^ With aryl‐substituted acetylenes, the method requires somewhat forcing conditions (high temperatures and solvent‐free) to facilitate the reaction of the vinyl triflate intermediate **A** with the aryl sulfoxides (Scheme 1 b).

Our group has a long‐standing interest in the chemoselective triflic‐anhydride‐mediated activation of amides, stemming from the pioneering early work of Ghosez^[9]^ and more recent reports by Charette,^[10]^ Movassaghi,^[11]^ Huang and others.^[12]^ Upon treatment of an amide with triflic anhydride and a base, a highly electrophilic keteniminium intermediate **B** is generated (Scheme 1 c). **B** can undergo a variety of transformations with nucleophilic species, including aryl sulfoxides, leading to α‐arylation via a [3,3]‐sigmatropic rearrangement,^[13]^ or TEMPO to furnish α‐oxyaminated amides following an umpolung event (Scheme 1 c).^[14]^ We hypothesized whether putative analogues of the pivotal keteniminium could be generated when the lone pair of the nitrogen atom of the amide functionality is replaced with another strong electron‐donor, such as electron‐rich aromatic rings, leading us to propose *para*‐methoxy‐substituted acetophenones. The highly reactive analogous intermediate **C** might reside in equilibrium with the vinyl triflate **A′** (Scheme 1 c). Although the formation of vinyl triflates from the corresponding acetophenones is well‐established,^[15]^ and they can be often purified by chromatography and isolated, the reactivity of such species with electron‐donating groups at the *para*‐position is scarcely reported due to their decreased stability.^[16]^

Herein, we report, to the best of our knowledge, the first example of electrophilic ketone activation and subsequent metal‐free α‐arylation and oxyamination.

We first set out to attempt α‐arylation of ketones by using 4‐methoxyacetophenone and diphenyl sulfoxide as model substrates (Scheme 2 a, for full optimization details, see the SI), investigating a range of conditions. Initially, standard procedures used for amide activation (Tf_2_O, 2‐I‐pyr in DCE at 0 °C for 15 min, then addition of the sulfoxide) gave the target α‐arylated product **2 a** in a moderate yield. Pleasingly, stoichiometric amounts of a bulky, non‐nucleophilic base (2,6‐di‐*tert*‐butyl‐4‐methylpyridine, DTBMP) led to an increase in yield and even allowed us to scale up the reaction to 7 mmol (60 % yield). On the other hand, the use of an excess of the base afforded the corresponding phenylacetylene. As alluded to previously, attempted isolation of the intermediate enol triflate failed due to decomposition.

**Scheme 2:**
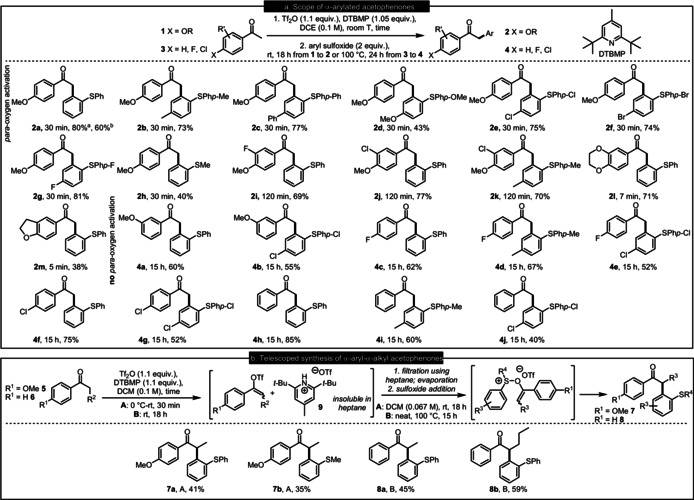
a) Scope of α‐arylation of ketones and b) telescoped synthesis of α‐aryl‐α‐alkyl ketones; ^a^ 0.2 mmol scale; ^b^ 7.0 mmol scale; DTBMP −2,6‐di‐*tert*‐butyl‐4‐methyl‐pyridine.

With the optimized conditions in hand, we investigated the scope of the methodology by screening various sulfoxides with the model substrate 4‐methoxyacetophenone (Scheme 2 a). A variety of substituents was tolerated at the *para*‐position of the aryl sulfoxides, including the electron‐donating methyl, phenyl and methoxy groups (**2 b**–**d**), as well as halogens (**2 e**–**g**). Additionally, an aryl alkyl sulfoxide could also be employed, affording **2 h**. We then moved on to investigate the scope of 4‐methoxyacetophenone derivatives and how the substitution pattern on the aromatic ring affects the duration of the ketone activation, the completion of which is indicated by intensely purple colour of the reaction mixture. With halogens at the 3‐position of the aromatic ring, the activation time prior to the addition of sulfoxide had to be extended to 120 min. Nevertheless, the corresponding products were obtained in excellent yields (**2 i**–**k**). Lastly, we explored substrates where the activating oxygen atom in the *para*‐position of the aromatic rings is incorporated within a ring. Six‐ and five‐membered cyclic substrates proved more reactive than the model substrate 4‐methoxyacetophenone, necessitating activation for shorter time to give products **2 l**,**m**.

Encouraged by the development of this procedure for the metal‐free α‐arylation of 4‐methoxyacetophenone and its derivatives, we aimed to extend this one pot procedure to cases where there is no strongly electron‐donating group in the *para* position of the ketone's aromatic ring (Scheme 2 a). As expected, longer reaction times were needed for activation under conditions akin to those applied to *para*‐oxygen activated substrates.[^15a^, ^15b^, ^17^]

3‐Methoxyacetophenone (**4 a**,**b)**, 4‐fluoroacetophenone (**4 c**–**e**), 4‐chloroacetophenone (**4 f**,**g**) and acetophenone (**4 h**–**j**) gave good yields with diphenyl sulfoxide as well as its *para*‐substituted analogues carrying methyl‐ or chloro‐ substituents.

Unfortunately, with 4‐methoxypropiophenone and diphenyl sulfoxide, the one‐pot approach did not furnish the target compound in satisfactory yields. Monitoring the activation of 4‐methoxypropiophenone by ^1^H NMR showed that the consumption of the starting material to generate the vinyl triflate reaction intermediate **A** competes with the decomposition of the latter (see the SI for details). Hence, we hypothesized that the observed low yields may stem from the presence of the pyridinium triflate **9** in the reaction mixture after the generation of the reactive intermediate (Scheme 2 b). To this end, a telescoped approach, in which we added a non‐polar solvent after the generation of vinyl triflate, in order to precipitate **9**, was developed. This protocol increased the yields of α‐aryl‐α‐alkyl ketones **7** to synthetically useful levels and was then applied to unactivated acetophenones, also increasing the yields of α‐aryl‐α‐alkyl ketones **8** with respect to the one‐pot procedure.

Inspired by previous work in our group,^[14]^ we then sought application of this novel ketone activation to α‐oxyamination (Scheme 3 a).^[18]^ Following removal of pyridinium triflate **9**, we substituted addition of an aryl sulfoxide for an excess of TEMPO to yield products **10**. As previously observed upon α‐arylation, 4‐methoxyacetophenone and its 3‐haloanalogues gave the best yields for products **10 a**–**c**. The six‐membered ring substrate gave **10 d** in a similar yield, while the more reactive **10 e**,**f** worked less well.

**Scheme 3:**
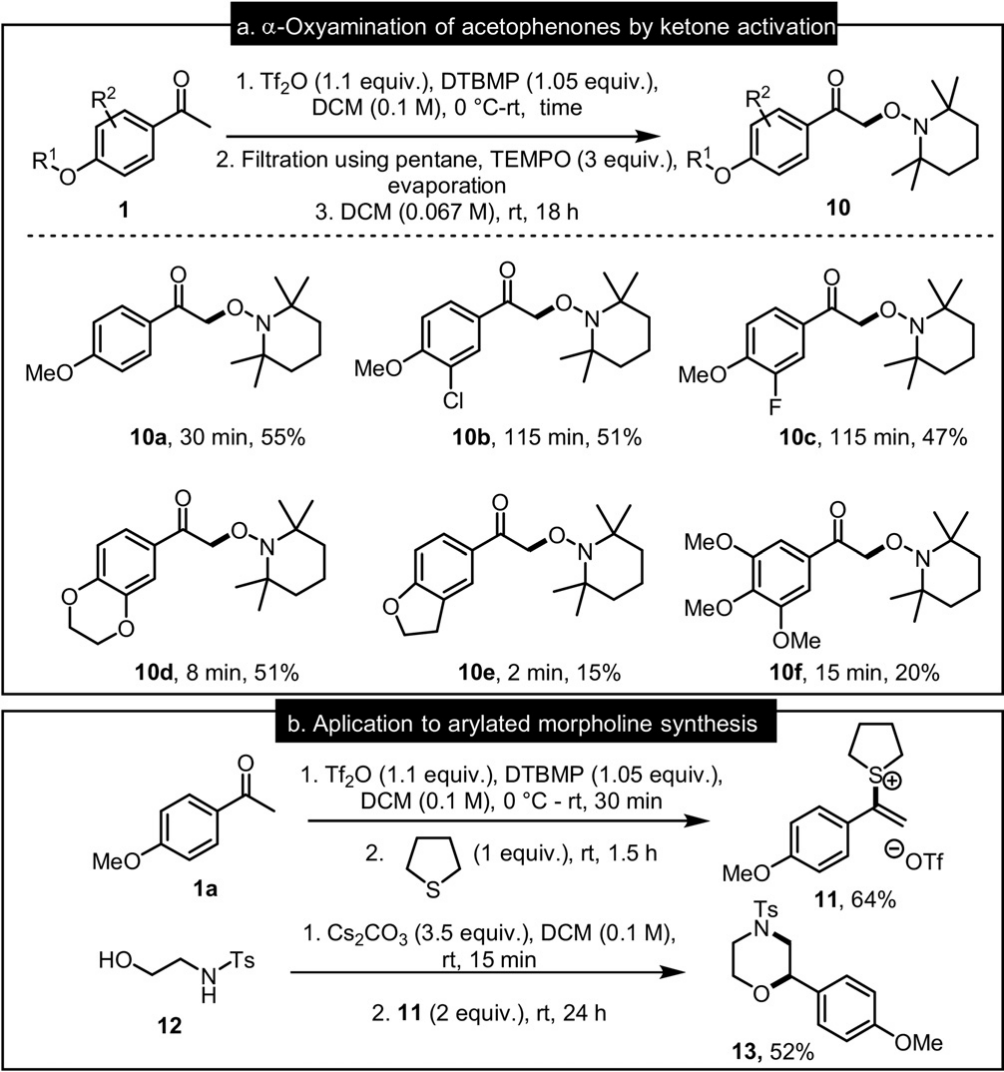
a) α‐Aminoxylation of acetophenones; b) Application of ketone activation to the synthesis of a morpholine.

The highly reactive intermediates generated by ketone activation can be captured by other nucleophiles than aryl sulfoxides or TEMPO (Scheme 3 b). For instance, addition of tetrahydrothiophene allows the obtention of vinylsulfonium triflate **11**. After isolation by aqueous work‐up without further purification, and following Aggarwal's methodology,^[19]^ **11** was treated with *N*‐tosyl‐aminoethanol **12** under basic conditions to obtain morpholine **13**.

Following our findings and the observation that acetophenones with electron donating *para*‐substitution are activated much more easily than their unsubstituted counterparts, we studied this electrophilic ketone activation computationally. Figure 1 shows the computed reaction profile for the conversion of the initial intermediate **A** into the keteniminium analogue **C** via the vinyl triflate **B**. Three systems are presented for comparison: the system **I** derived from *para*‐methoxy‐acetophenone, **II**—from the dihydrofuran‐substituted acetophenone and **III**—from unsubstituted acetophenone.

**Figure 1:**
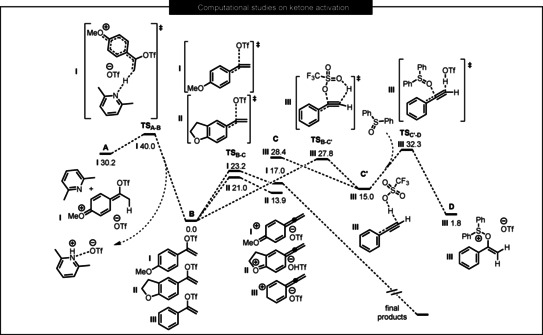
Computed relative free energy profile (DLPNO‐CCSD(T)/def2‐TZVP//B3LYP‐D3/def2‐SVP, Δ*G*_298,DCM_, kcal mol^−1^) for the conversion of the intermediate **A** to the intermediate **C** (**C′** in the case of the system **III**) via vinyl triflate **B** (taken as a reference 0.0 kcal mol^−1^). The intermediate **C** (**C′**) leads to the final products. The pathway for the transformation of the intermediate **C′** to the precursor of the sigmatropic rearrangement **D** is also depicted. See the SI for computational details.

The first step is deprotonation by the base (2,6‐dimethylpyridine is used for the calculations), leading to the vinyl triflate **B** via the transition state **TS_A‐B_** with a small barrier of Δ*G* ^≠^(**A**→**B, I**)=9.8 kcal mol^−1^. This step is highly exergonic (Δ*G*(**A**→**B, I**)=−30.2 kcal mol^−1^).

In the next step, the C(sp^2^)−O bond with the triflate fragment is broken and the intermediate **C** is formed via the transition state **TS_B‐C_**. The computed barrier of the **B**→**C** step is substantially lower for the cyclic system **II**: ΔΔ*G* ^≠^= Δ*G* ^≠^(**B**→**C, I**)−Δ*G* ^≠^(**B**→**C, II**)=2.2 kcal mol^−1^. This means, in accordance with the Eyring equation, that the half‐life time (*t*_1/2_) of the step **B**→**C** for system **II** is approx. 40 times smaller as compared to system **I**. This result agrees well with the experimental evidence. Intermediate **C** is also found to be 3.1 kcal mol^−1^ more stable for system **II**. In the case of system **III**, intermediate **C** is highly destabilized (Δ*G*(**B**→**C, III**)=28.4 kcal mol^−1^) and undergoes a barrierless rearrangement to the deprotonated form intermediate **C′** with a distinctive hydrogen bond, as shown in Figure 1. The intermediate **C′** is connected to the corresponding vinyl triflate **B** via transition state **TS_B‐C′_** with the very high barrier of Δ*G* ^≠^(**B**→**C′**)=27.8 kcal mol^−1^. The latter explains the experimental observation that elevated temperatures are required for the reaction with unsubstituted acetophenone. The second step **B**→**C** is computed to be endergonic. However, intermediate **C** undergoes additional steps leading to the final products making the overall process thermodynamically accessible. Our calculations show that in the case of the system **III** the sigmatropic rearrangement precursor intermediate **D** can be obtained directly from intermediate **C′** via the transition state **TS_C′‐D_**.

It is worth noting that calculations deny the possibility of a concerted conversion of the intermediate **A** to **C**. The mechanism is stepwise and requires the formation of the intermediate **B**.

In summary, we have shown that the electrophilic ketone activation enables α‐arylation, ‐oxyamination and formation of vinylsulfonium salts from acetophenones under mild conditions in good to excellent yields. Stabilisation of the acetophenone‐derived vinyl cationic species with oxygen atoms at the *para* position of the aromatic ring leads to a reaction pathway resembling that of electrophilic amide activation and leading to intermediate keteniminium ion analogues. Computational studies support the experimental observations and explained differences in reactivity between different vinyl triflates.

## Conflict of interest

The authors declare no conflict of interest.

## Supporting information

As a service to our authors and readers, this journal provides supporting information supplied by the authors. Such materials are peer reviewed and may be re‐organized for online delivery, but are not copy‐edited or typeset. Technical support issues arising from supporting information (other than missing files) should be addressed to the authors.

Supplementary
