# Supplementary material for: α‐Functionalisation of Ketones Through Metal‐Free Electrophilic Activation
Source: Angew Chem Int Ed Engl. 2020 Sep 11;59(47):20935–9. doi: 10.1002/anie.202006398 (PMC7693173; doi:10.1002/anie.202006398)
Supplement: Supplementary file 1 — Supplementary [file ANIE-59-20935-s001.pdf]

Angewandte Chemie
Eine Zeitschrift der Gesellschaft Deutscher Chemiker

Supporting Information

α-Functionalisation of Ketones Through Metal-Free Electrophilic Activation

*Wojciech Zawodny, Christopher J. Teskey, Magdalena Mishevska, Martin Völkl, Boris Maryasin, Leticia González, and Nuno Maulide**

anie_202006398_sm_miscellaneous_information.pdf

Table of contents

1 Experimental section 2

1.1 General experimental information 2

1.2 Explanation of terms 3

1.3 General procedures 3

1.4 Optimisation of alpha-arylation of 4-methoxyacetophenone 6

1.5 Detection of vinyl triflate intermediate using 1H NMR 7

1.5.1 Monitoring ketone activation of 4-methoxypropiophenone by 1H NMR 10

1.6 Synthesis of starting material sulfoxides 12

1.7 Characterisation data 14

1.7.1 Alpha-arylation of ketones 14

1.7.2 Alpha-aminoxylation of ketones 32

1.8 Formation of vinylsulfonium salts 36

2 NMR printouts 39

3 Computational details 74

3.1 Coordinates of the most stable ($\Delta G_{298,DCM}$) conformations as computed at the DLPNO-CCSD(T)/def2-TZVP//B3LYP-D3/def2-SVP level of theory 74

4 References 81

1 Experimental section

1.1 General experimental information

Unless otherwise stated, all glassware was flame-dried before use and all reactions were performed under an atmosphere of argon. All solvents were distilled from appropriate drying agents prior to use or directly taken from commercial sealed bottles under an atmosphere of argon. All reagents were used as received from commercial suppliers unless otherwise stated. Triflic anhydride (trifluoromethanesulfonic acid anhydride) was distilled over phosphorus pentoxide prior to use. Reaction progress was monitored by thin layer chromatography (TLC) performed on aluminum plates coated with silica gel F254 with 0.2 mm thickness. Chromatograms were visualized by fluorescence quenching with UV light at 254 nm or by staining using potassium permanganate. Flash column chromatography was performed using silica gel 60 (230-400 mesh, Merck and co.). Neat infra-red spectra were recorded using a Brucker Vertex 70 FT-IR spectrometer. Wavenumbers are reported in cm^{-1}. Mass spectra were obtained using a Brucker maXis UHR-TOF spectrometer, using electrospray ionization (ESI) and by Agilent 7200B GC/Q-TOF spectrometer, using electron impact (EI). All ^{1}H NMR and ^{13}C NMR spectra were recorded using a Bruker AV NEO 500, AV III 600 or AV III HD 700 spectrometer in $CDCl_3$. Chemical shifts were given in parts per million (ppm, δ), referenced to the solvent peak of $CDCl_3$, defined at δ = 7.26 ppm (^{1}H NMR) and δ = 77.16 (^{13}C NMR). Coupling constants are quoted in Hz (*J*). ^{1}H NMR splitting patterns were designated as singlet (s), doublet (d), triplet (t), quartet (q), pentet (p). Splitting patterns that could not be interpreted or easily visualized were designated as multiplet (m) or broad (br).

1.2 Explanation of terms

For convenience, the following terms will be used in the procedures below:

Activated acetophenone – acetophenone incorporating an oxygen atom at the *para* position of the aromatic ring, increasing its reactivity and therefore requiring shorter activation time (usually no longer than 2 hours).

Unactivated acetophenone – acetophenone without an oxygen atom at the *para* position of the aromatic ring, requiring longer activation times (typically 15-18 hours).

Activation time - time between the addition of triflic anhydride and the next step of the synthesis, i.e. addition of aryl sulfoxide or addition of apolar solvent and filtration of the pyridinium triflate present in the reaction mixture).

1.3 General procedures

General procedure A1 for one pot ketone activation and interception by aryl sulfoxide from activated acetophenones:

To a flame dried Schlenk tube under argon with a stirring bar and solution of a ketone (1.0 equiv.), 2,6-di-*tert*-butyl-4-methylpyridine (1.05-1.1 equiv.) in DCE (0.1 M) was added triflic anhydride (1.1 equiv.) at room temperature (unless lower temperature is stated). The reaction mixture was stirred at room temperature for 30 min (for 4-methoxyacetophenone as a substrate) or other time stated (up to 120 min depending on the acetophenone used – referred to later as activation time). To the reaction mixture at room temperature was then added the aryl sulfoxide (2.0 equiv.). The reaction mixture was stirred for 18 h at room temperature. Water was added to quench the reaction mixture. The reaction mixture was extracted with DCM (3x), dried over sodium sulfate, evaporated *in vacuo* and purified by normal phase flash column chromatography using a suitable mixture of solvents. Usual solvent mixtures for column chromatography were heptane-ethyl acetate, heptane-DCM or DCM-toluene (in the latter case, this eluent was used when the TLC separation between the starting material and product with other eluents was small).

General procedure A2 for one pot ketone activation and interception by aryl sulfoxide from unactivated acetophenones:

To a flame dried Schlenk tube under argon equipped with a stirring bar and a solution of a ketone (1.0 equiv.), 2,6-di-*tert*-butyl-4-methylpyridine (1.05-1.1 equiv.) in DCE (0.1 M) at room temperature was added triflic anhydride (1.1 equiv.). The reaction mixture was stirred at room temperature for 15 h. To the reaction mixture at room temperature was added the

aryl sulfoxide (2.0-3.0 equiv.). The reaction mixture was heated at 100 °C in a sealed tube for 24 h. The reaction mixture was allowed to cool down to room temperature, and water was added to quench the reaction mixture. Then it was extracted with DCM (3x), dried over sodium sulfate, evaporated *in vacuo* and purified by normal phase flash column chromatography using a suitable mixture of solvents. Usual solvent mixtures for column chromatography were heptane-ethyl acetate, heptane-DCM or DCM-toluene (in the latter case, this eluent was used when the TLC separation between the starting material and product with other eluents was small).

General procedure B1 for telescopic alpha arylation of activated ketones:

To a flame dried Schlenk tube under argon equipped with a stirring bar and a solution of a ketone (1.0 equiv.), 2,6-di-*tert*-butyl-4-methylpyridine (1.05-1.1 equiv.) in DCM (0.1 M) at 0 °C was added triflic anhydride (1.1 equiv.). The reaction mixture was allowed to warm up to room temperature and stirred at room temperature for 30 min (for 4-methoxyacetophenone or 1-(4-methoxyphenyl)propan-1-one as a substrate) or other time stated with other substrates. To the reaction mixture at room temperature was added heptane* (approx. 20 mL), and the resulting precipitate of 2,6-di-*tert*-butyl-4-methylpyridinium triflate was filtered over a sintered funnel. The precipitate was washed with extra heptane. Aryl sulfoxide (2.0 equiv.) was added and the reaction mixture was evaporated *in vacuo*, then the residue was dissolved in DCM (0.067 M), and the reaction mixture was stirred at room temperature for 18 h. The reaction mixture was quenched with distilled water, and extracted with DCM from distilled water (2x). The combined organic extract was was dried over sodium sulfate, evaporated *in vacuo* and purified by normal phase flash column chromatography using a suitable mixture of solvents.

General procedure B2 for telescopic alpha arylation of unactivated ketones:

To a flame dried Schlenk tube under argon equipped with a stirring bar and a solution of a ketone (1.0 equiv.), 2,6-di-*tert*-butyl-4-methylpyridine (1.05-1.1 equiv.) in DCM (0.1 M) at room temperature was added triflic anhydride (1.1 equiv.). The reaction mixture was stirred at room temperature for 18 h. To the reaction mixture at room temperature was added heptane* (approx. 20 mL), and the resulting precipitate of 2,6-di-*tert*-butyl-4-methylpyridinium triflate was filtered over a sintered funnel. The precipitate was washed with extra heptane. Aryl sulfoxide (2.0-3.0 equiv.) was added and the reaction mixture was evaporated *in vacuo*. The reaction mixture was heated neat at 100 °C in a sealed tube for 15 h. The reaction mixture was allowed to cool down to room temperature, extracted with

DCM from distilled water (2x). The combined extract was dried over sodium sulfate, evaporated *in vacuo* and purified by normal phase flash column chromatography using a suitable mixture of solvents.

General procedure C for telescopic alpha-aminoxylation of activated ketones:

To a flame dried Schlenk tube under argon equipped with a stirring bar and a solution of a ketone (1.0 equiv.), 2,6-di-*tert*-butyl-4-methylpyridine (1.05-1.1 equiv.) in DCM (0.1 M) at room temperature was added triflic anhydride (1.1 equiv.). The reaction mixture was stirred at room temperature for 30 min (for 4-methoxyacetophenone as a substrate) or other time stated. To the reaction mixture at room temperature was added pentane* (approx. 20 mL), and the resulting precipitate of 2,6-di-*tert*-butyl-4-methylpyridinium triflate was filtered over a sintered funnel. The precipitate was washed with extra pentane. TEMPO (3.0 equiv.) was added and the reaction mixture was evaporated *in vacuo*, then dissolved in DCM (0.067 M), and stirred at room temperature for 18 h. The reaction mixture was extracted with DCM from distilled water, dried over sodium sulfate, evaporated *in vacuo* and purified by normal phase flash column chromatography using a suitable mixture of solvents.

*For the telescopic procedures above including filtration of the 2,6-di-*tert*-butyl-4-methylpyridinium triflate salt, both pentane and heptane can be used. The advantage of pentane is that it the reaction mixture is not evaporated to dryness when DCE is used as a solvent for ketone activation; similarly, DCM can be replaced with DCE during activation and after addition of sulfoxide.

1.4 Optimisation of alpha-arylation of 4-methoxyacetophenone

General procedure A1 was employed for optimisation (using 0.2 mmol of 4-methoxyacetophenone and 1 equiv. of bromoform (19.2 µL, 0.2 mmol) added after work-up to determine the 1H NMR yield.

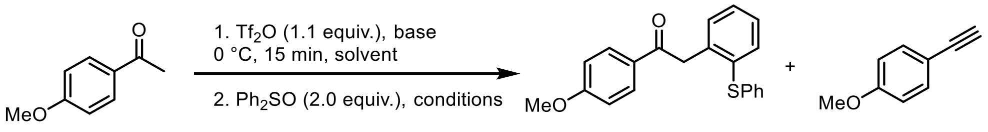

1 2

Entry	Base (equiv.)	Solvent	Conditions	NMR yields	
				1	2
**1**	**2-iodopyridine (2.2)**	**DCE**	**0 ° -> rt, o/n**	**30%**	nd
**2**	**2-iodopyridine (2.2)**	**DCM**	**0 ° -> rt, o/n**	**40%**	nd
**3**	2-iodopyridine (2.2)	DCE	100 °C, 3.5 h	36%	nd
**4**	2-iodopyridine (2.2)	toluene	100 °C, 3.5 h	0%	nd
**5**	2-iodopyridine (2.2)	MeCN	100 °C, 3.5 h	10%	nd
**6**	2-iodopyridine (3.3)	DCM	0 ° -> rt, 18 h	16%	nd
**7**	2-iodopyridine (1.1)	DCE	0 ° -> rt, 18 h	19%	nd
**8**	2-fluoropyridine (2.2 equiv.)	DCE	0 ° -> rt, 18 h	2%	nd
**9**	2-chloropyridine (2.2 equiv.)	DCE	0 ° -> rt, 18 h	13%	nd
**10**	2-bromopyridine (2.2)	DCE	0 ° -> rt, 18 h	23%	nd
**11**	2-methoxypyridine (2.2)	DCE	0 ° -> rt, 18 h	trace	nd
**12**	NEt_3 (2.2 equiv.)	DCE	0 ° -> rt, 18 h	0%	nd
**13**	2-methylpyridine	DCE	0 ° -> rt, 18 h	2%	nd
**14**	-	DCE	0 ° -> rt, 18 h	0%	nd
**15**	2,6-di-*t*-Bu-4-MePyr (3.3 equiv.)[a]	DCE	rt, 18 h	42%	35%
**16**	2,6-di-*t*-Bu-4-MePyr (3.3 equiv.)[a]	DCE	rt, 18 h	0%	69%
**17**	2,6-di-*t*-Bu-4-MePyr (1.1 equiv.)[b]	DCE	rt, 18 h	76%	0%
**18**	2,6-di-*t*-Bu-4-MePyr (1.1 equiv.)[b,c]	DCE	rt, 18 h	55%	0%
**19**	2,6-di-*t*-Bu-4-MePyr (1.1 equiv.)[b]	DCE	rt, 3 h	78%	0%
**20**	2,6-di-*t*-Bu-4-MePyr (1.1 equiv.)[b]	DCE	100 °C, 3 h	76%	0%

[a]Tf_2O added at rt and stirred for 120 min before Ph_2SO addition; [b]Tf_2O added at rt and stirred for 30 min; [c]1.1 equiv. of Ph_2SO used; nd – not determined.

1.5 Detection of vinyl triflate intermediate using 1H NMR

To optimise the conditions for ketone activation, we performed 1H NMR experiments in deuterated DCM to determine the timescale of vinyl triflate formation and its decomposition. With just 1.1 equivalent of 2,6-di-*tert*-butyl-4-methylpyridine, the vinyl triflate formed within a few minutes after the triflic anhydride addition, and was stable in solution for several hours (no major changes after 6 h). With 2.2 equivalents of the base, an alkyne formed over time.

The vinyl triflate derived from 4-methoxyacetophenone is only stable in solution and does not survive concentration *in vacuo* or aqueous work-up, hydrolyzing back to 4-methoxyacetophenone.

The following experiments were performed to investigate the conversion of starting material and the ratio of reactive intermediate and alkyne formed. The doublets at 5.51 and 5.26 ppm indicated the presence of vinyl triflate as an intermediate.

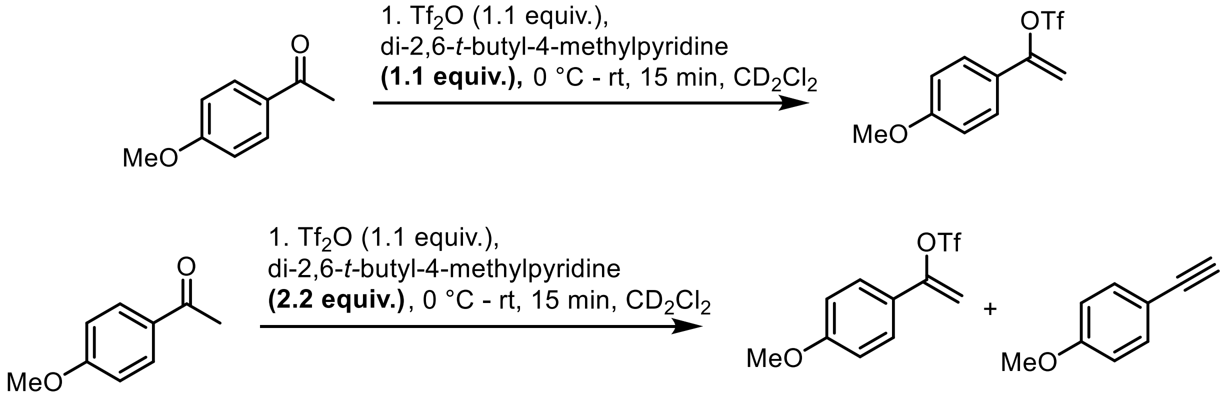

General procedure A1 was followed. To a dried Schlenk tube under argon with a solution of a 4-methoxyacetophenone (15 mg, 0.1 mmol), 2,6-di-*tert*-butyl-4-methylpyridine (22.6 mg, 0.11 mmol or 43.2 mg, 0.22 mmol) in CD_2Cl_2 (1 mL) at 0 °C was added triflic anhydride (18.5 µL, 0.11 mmol). The reaction mixture (0.5 mL) transferred to an NMR tube and the reaction was monitored by 1H NMR every 15 minutes for 6 hours (the first measurement was performed after 15 minutes). No starting material was observed in the NMR after the first measurement.

Ratio of vinyl triflate and alkyne when performing ketone activation with 1.1 equiv. and 2.2 equiv. of 2,6-di-*tert*-butyl-4-methylpyridine:

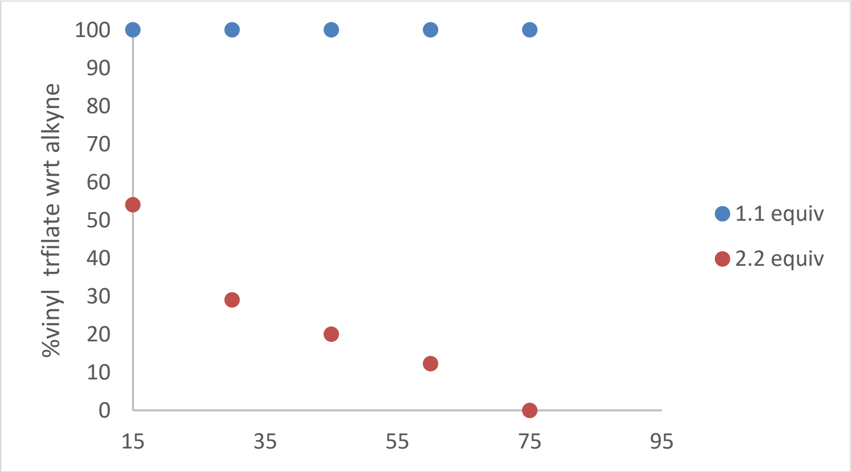

1H NMR in CD_2Cl_2 with 1.1 equiv. of 2,6-di-*tert*-butyl-4-methylpyridine and 30 minutes after Tf_2O addition

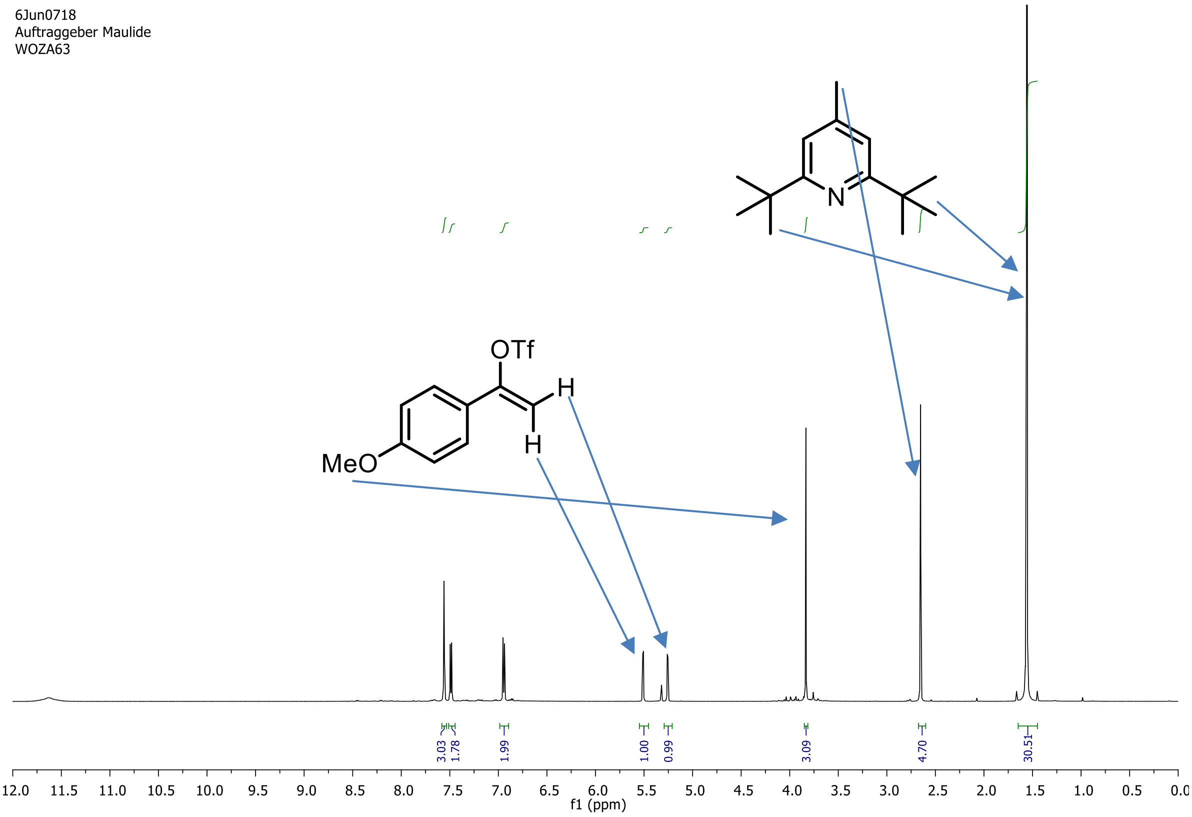

1H NMR in CD_2Cl_2 with 2.2 equiv. of 2,6-di-*tert*-butyl-4-methylpyridine and 15 minutes after Tf_2O addition

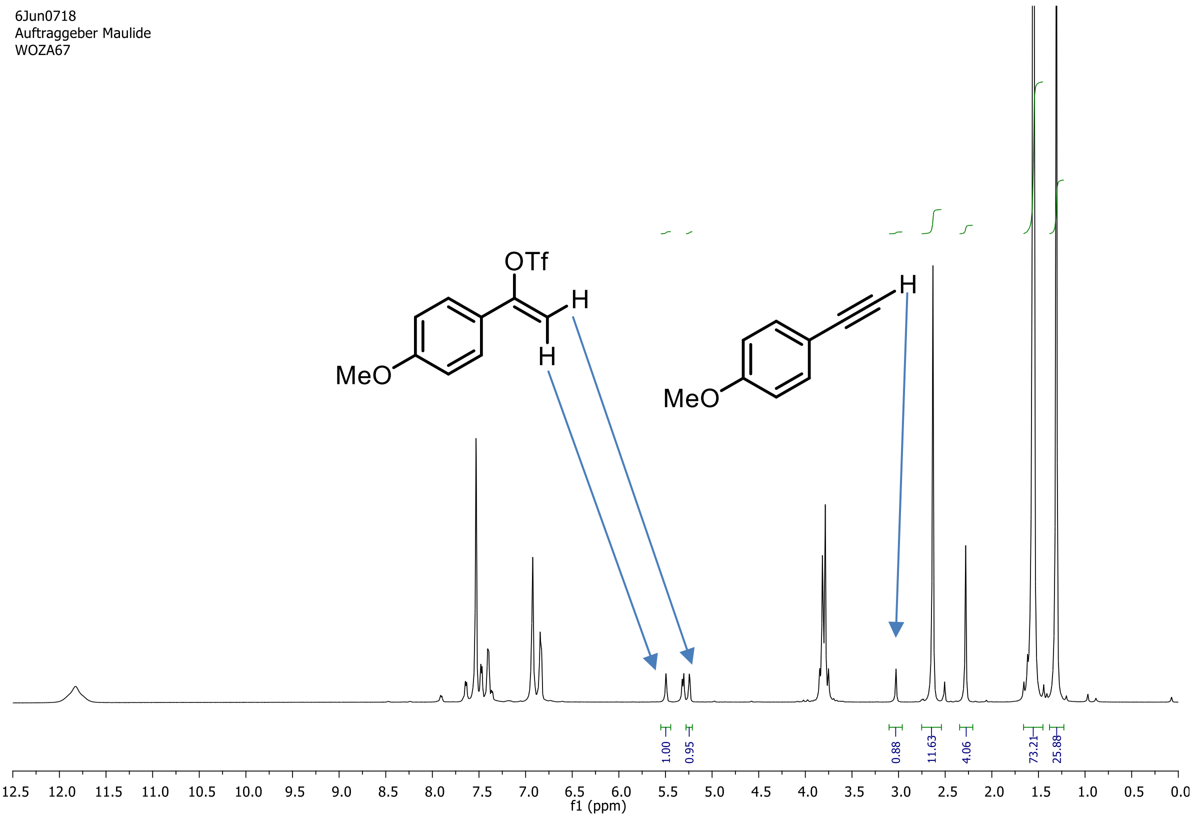

1.5.1 Monitoring ketone activation of 4-methoxypropiophenone by 1H NMR

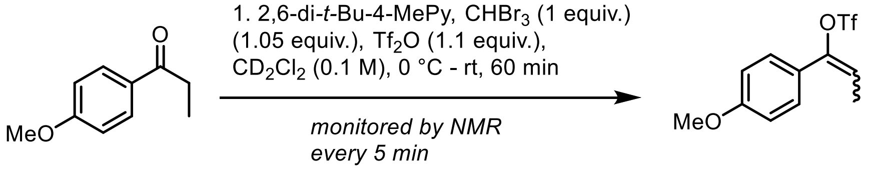

Scheme 1. Monitoring of formation of vinyl triflate derived from 4-methoxypropiophenone by 1H NMR.

To a dried Schlenk tube under argon with a solution of a 4-methoxypropiophenone (30.7 µL, 0.2 mmol), 2,6-di-*tert*-butyl-4-methylpyridine (43.1 mg, 0.21 mmol), bromoform (19.2 µL, 0.2 mmol) in CD_2Cl_2 (2 mL) at 0 °C was added triflic anhydride (37 µL, 0.22 mmol). The reaction mixture (0.5 mL) was transferred to an NMR tube and the reaction was monitored by 1H NMR every 5 minutes. NMR yields of starting material and reactive species form were determined with respect to the bromoform 1H NMR shift.

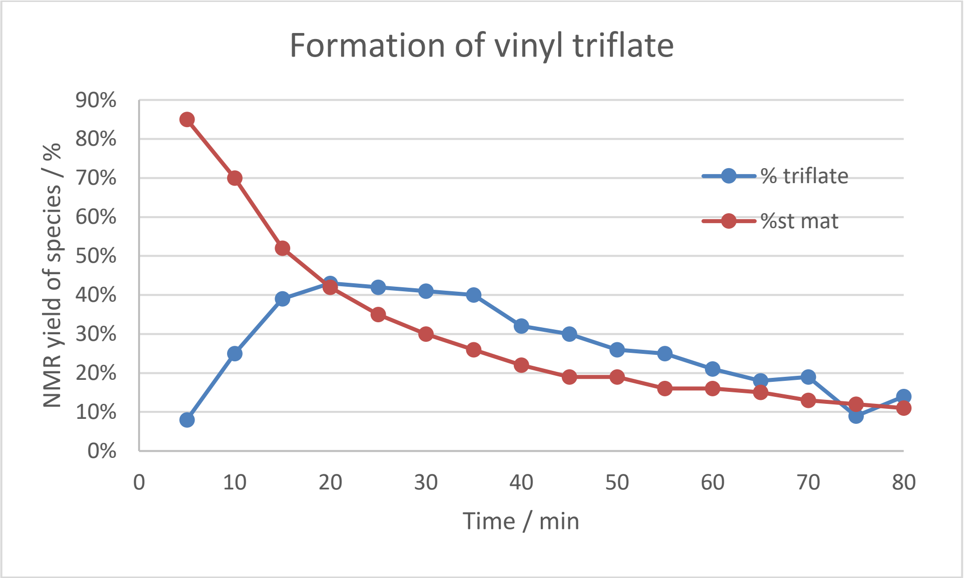

1H NMR in CD_2Cl_2 10 minutes after Tf_2O addition

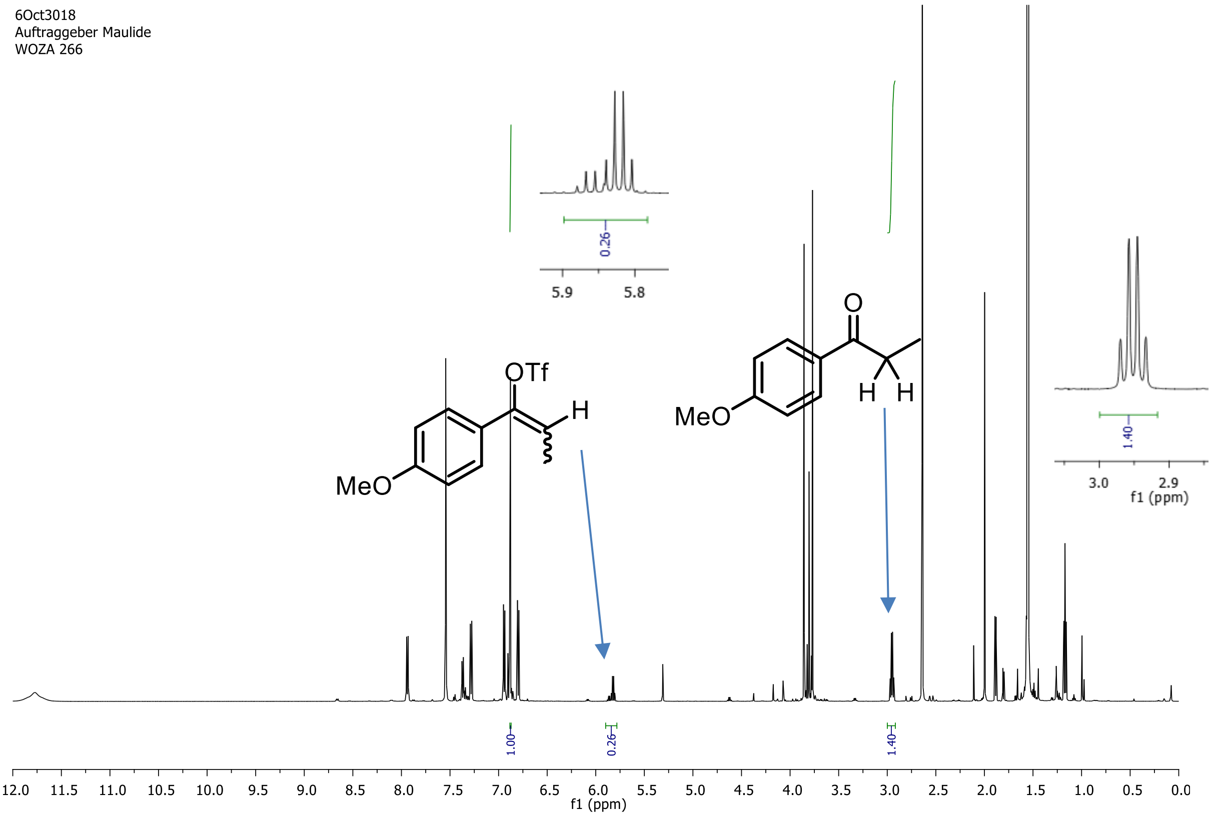

1.6 Synthesis of starting material sulfoxides

4,4'-Sulfinylbis(methoxybenzene)

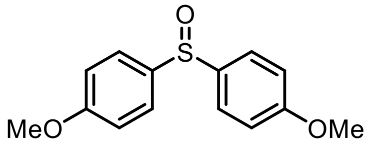

4'-Sulfinylbis(methoxybenzene) was prepared according to a literature procedure[1] on a 5 mmol scale and obtained as a white solid (1.08 g, 82% yield). The spectral properties are in agreement with those reported in the literature.

4,4''-Sulfinyldi-1,1'-biphenyl

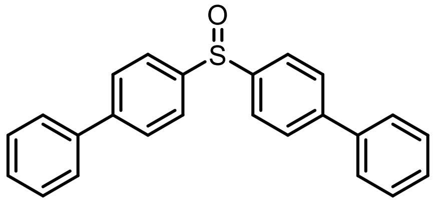

4,4''-Sulfinyldi-1,1'-biphenyl was prepared according to a literature procedure[2] on a 6 mmol scale and the title compound was obtained as a white solid (1.48 g, 70% yield). The spectral properties are in agreement with those reported in the literature.

4,4'-Sulfinylbis(bromobenzene)

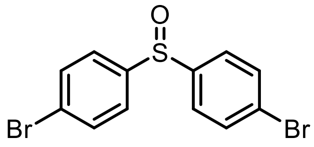

4,4'-Sulfinylbis(bromobenzene) was prepared according to a literature procedure[1] on a 10 mmol scale and was obtained (1.82 g, 50 % yield) as a white solid. The spectral properties are in agreement with those reported in the literature.

4,4'-Sulfinylbis(fluorobenzene)

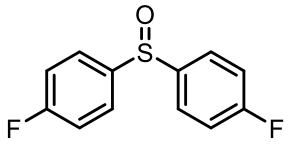

4,4'-Sulfinylbis(fluorobenzene) was prepared according to a literature procedure[1] on a 10 mmol scale and was obtained (1.29 g, 54% yield) as a yellow oil. The spectral properties are in agreement with those reported in the literature.

1.7 Characterisation data

1.7.1 Alpha-arylation of ketones

1-(4-methoxyphenyl)-2-(2-(phenylthio)phenyl)ethan-1-one 2a

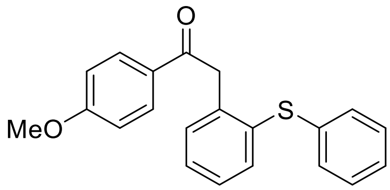

Following General procedure A1 using 4-methoxyacetophenone (0.030 g, 0.2 mmol), 2,6-di-*tert*-butyl-4-methylpyridine (0.0452 g, 0.22 mmol), trifluoromethanesulfonic acid anhydride (37 µL, 0.22 mmol) and diphenyl sulfoxide (0.0809 g, 0.4 mmol) gave after 30 min of activation and after purification by flash column chromatography (SiO_2, heptane-EtOAc 19:1, then 14:1, then 9:1) target compound **2a** as a yellow solid (0.0535 g, 80%).

R_f 0.5 (heptane:EtOAc 7:3).

^{1}H NMR (400 MHz, $CDCl_3$) δ 7.90 – 7.82 (m, 2H), 7.36 (d, *J* = 7.7 Hz, 1H), 7.21 (d, *J* = 4.0 Hz, 2H), 7.18 – 7.11 (m, 3H), 7.10 – 7.03 (m, 3H), 6.84 – 6.77 (m, 2H), 4.34 (s, 2H), 3.76 (s, 3H).

^{13}C NMR (151 MHz, $CDCl_3$) δ 195.9 (CO), 163.6 (*C*), 138.1 (*C*), 136.9 (*C*), 134.7 (*C*H), 134.0 (*C*), 131.3 (*C*H), 130.8 (2 x *C*H), 129.9 (*C*), 129.3 (2 x *C*H), 129.2 (2 x *C*H), 128.6 (*C*H), 128.1 (*C*H), 126.4 (*C*H), 113.8 (2 x *C*H), 55.6 (OCH_3), 43.6 (CH_2).

IR (neat) ν/cm^{-1}: 2922, 2851, 1677, 1599, 1467, 1261, 1169, 738.

HRMS (ESI+): exact mass for $[M+Na]^+$ ($C_{21}H_{18}O_2SNa^+$) calculated: 357.0920, found: 357.0919.

The data are in accordance with those reported previously.[3]

1-(4-methoxyphenyl)-2-(5-methyl-2-(p-tolylthio)phenyl)ethan-1-one 2b

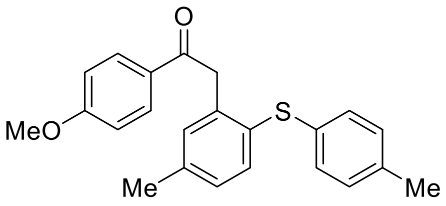

Following General procedure A1 using 4-methoxyacetophenone (0.030 g, 0.2 mmol), 2,6-di-*tert*-butyl-4-methylpyridine (0.0452 g, 0.22 mmol), trifluoromethanesulfonic acid anhydride (37 µL, 0.22 mmol) and *p*-tolyl sulfoxide (0.0921 g, 0.4 mmol) gave after 30 min of activation and purification by flash column chromatography (SiO_2, heptane-EtOAc 19:1, then 14:1, then 9:1) target compound **2b** as a colourless oil (0.053 g, 73%).

R_f 0.48 (heptane:EtOAc 7:3).

^{1}H NMR (600 MHz, $CDCl_3$) δ 8.00 – 7.93 (m, 2H), 7.37 – 7.31 (m, 1H), 7.13 – 7.10 (m, 1H), 7.08 – 7.01 (m, 5H), 6.93 – 6.88 (m, 2H), 4.38 (s, 2H, CH_2), 3.86 (s, 3H, OCH_3), 2.32 (s, CH_3), 2.29 (s, CH_3).

^{13}C NMR (151 MHz, $CDCl_3$) δ 196.2 (*C*O), 163.6 (*C*), 138.6 (*C*), 137.9 (*C*), 136.2 (*C*), 134.7 (*C*H), 133.7 (*C*), 131.9 (*C*H), 130.9 (2 x *C*H), 130.8 (*C*), 130.0 (*C*), 129.9 (2 x *C*H), 129.3 (2 x *C*H), 129.0 (*C*H), 113.8 (2 x *C*H), 55.6 (O*C*H_3), 43.5 (CH_2), 21.3 (*C*H_3), 21.1 (*C*H_3).

FTIR (neat) v_{max}, cm^{-1}: 2874, 1680, 1600, 1261, 1168, 831.

HRMS (ESI+): exact mass for $[M+H]^+$ ($C_{23}H_{23}O_2S^+$) calculated: 363.1413, found: 363.1413.

2-(4-([1,1'-biphenyl]-4-ylthio)-[1,1'-biphenyl]-3-yl)-1-(4-methoxyphenyl)ethan-1-one 2c

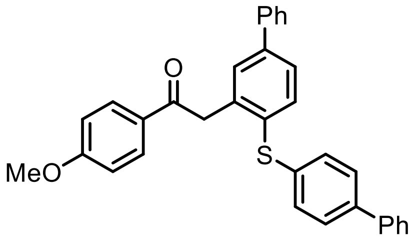

Following General procedure A1 using 4-methoxyacetophenone (0.030 g, 0.2 mmol), 2,6-di-*tert*-butyl-4-methylpyridine (0.0452 g, 0.22 mmol), trifluoromethanesulfonic acid anhydride (37 µL, 0.22 mmol) and 4,4''-sulfinyldi-1,1'-biphenyl (0.142 g, 0.4 mmol) gave after 30 min of activation and purification by flash column chromatography (SiO_2, DCM-heptane 1:1, then 3:1, then 4:1) target compound **2c** as a white solid (0.0745 g, 77%).

R_f 0.44 (heptane:EtOAc 7:3).

^{1}H NMR (600 MHz, $CDCl_3$) δ 8.00 (d, *J* = 8.8 Hz, 2H), 7.62 – 7.41 (m, 13H), 7.38 – 7.32 (m, 2H), 7.29 – 7.25 (m, 2H), 6.90 (d, *J* = 8.8 Hz, 2H), 4.55 (s, 2H, CH_2), 3.86 (s, 3H, OCH_3).

^{13}C NMR (151 MHz, $CDCl_3$) δ 195.8 (CO), 163.7 (*C*), 141.6 (*C*), 140.1 (*C*), 140.2 (*C*), 139.4 (*C*), 138.4 (*C*), 136.0 (*C*), 135.1 (*C*H), 132.9 (*C*), 130.9 (2 x *C*H), 130.1 (*C*H), 129.9 (*C*),

129.7 (2 x *C*H), 129.0 (2 x *C*H), 128.9 (2 x *C*H), 127.9 (2 x *C*H), 127.8 (*C*H), 127.5 (*C*H), 127.3 (2 x *C*H), 127.0 (2 x *C*H), 126.9 (*C*H), 113.9 (2 x *C*H), , 55.6 (O*C*H_3), 43.70 (*C*H_2).

FTIR (neat) ν_{max}, cm^{-1}: 2923, 1679, 1600, 1509, 1477, 1327, 1260, 1218, 1168, 990, 830, 760, 697.

HRMS (ESI+): exact mass for $[M+H]^+$ ($C_{33}H_{27}O_2S^+$) calculated: 487.1726, found: 487.1723

2-(5-methoxy-2-((4-methoxyphenyl)thio)phenyl)-1-(4-methoxyphenyl)ethan-1-one 2d

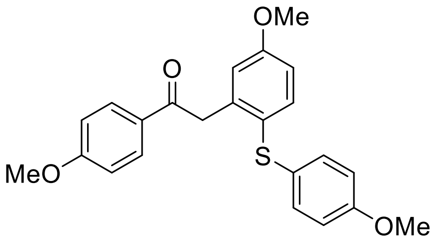

Following General procedure A1 using 4-methoxyacetophenone (0.030 g, 0.2 mmol), 2,6-di-*tert*-butyl-4-methylpyridine (0.0431 g, 0.21 mmol), trifluoromethanesulfonic acid anhydride (37 µL, 0.22 mmol) and 4,4'-sulfinylbis(methoxybenzene) (0.105 g, 0.4 mmol) gave after 30 min of activation and purification by flash column chromatography (SiO_2, heptane-EtOAc 19:1, then 9:1, then 5:1) target compound **2d** as a yellow oil (0.034 g, 43%).

R_f 0.27 (heptane:EtOAc 7:3).

^{1}H NMR (400 MHz, $CDCl_3$) δ 7.96 – 7.90 (m, 2H), 7.39 (d, *J* = 8.6 Hz, 1H), 7.11 – 7.05 (m, 2H), 6.93 – 6.87 (m, 2H), 6.85 – 6.82 (m, 1H), 6.79 – 6.73 (m, 3H), 4.39 (s, 2H), 3.86 (s, 3H), 3.78 (s, 3H), 3.76 (s, 3H).

^{13}C NMR (101 MHz, $CDCl_3$) δ 195.9 (*C*O), 163.6 (*C*), 159.9 (*C*), 158.6 (*C*), 139.7 (*C*), 136.3 (*C*H), 130.9 (2 x *C*H), 130.8 (2 x *C*H), 130.0 (*C*), 128.3 (*C*), 125.6 (*C*), 116.7 (*C*H), 114.9 (2 x *C*H), 113.9 (*C*H), 113.8 (2 x *C*H), 55.6 (O*C*H_3), 55.4 (2 x O*C*H_3), 43.8 (*C*H_2).

FTIR (neat) ν_{max}, cm^{-1}: 2936, 2836, 1627, 1597, 1493, 1243, 1170, 1028, 826 cm^{-1}.

HRMS (ESI+): calculated for $C_{23}H_{22}O_4SNa^+$ [$M+Na^+$] 417.1131, found: 417.1126.

2-(5-chloro-2-((4-chlorophenyl)thio)phenyl)-1-(4-methoxyphenyl)ethan-1-one 2e

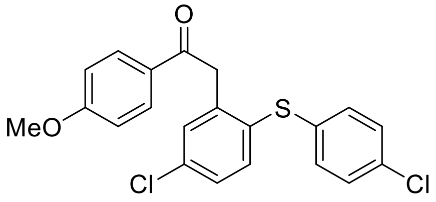

Following General procedure A1 using 4-methoxyacetophenone (0.030 g, 0.2 mmol), 2,6-di-*tert*-butyl-4-methylpyridine (0.0452 g, 0.22 mmol), trifluoromethanesulfonic acid anhydride (37 µL, 0.22 mmol) and 4,4'-sulfinylbis(chlorobenzene) (0.108 g, 0.4 mmol) gave after 30 min of activation and purification by flash column chromatography (SiO_2, heptane-EtOAc 19:1, then 14:1, then 9:1) target compound **2e** as a yellow oil (0.0605 g, 75%).

R_f 0.51 (heptane:EtOAc 7:3).

^{1}H NMR (400 MHz, $CDCl_3$) δ 7.95 – 7.89 (m, 2H), 7.38 – 7.30 (m, 2H), 7.24 (dd, J = 8.3, 2.3 Hz, 1H), 7.20 – 7.15 (m, 2H), 7.07 – 7.01 (m, 2H), 6.95 – 6.89 (m, 2H), 4.39 (s, 2H), 3.87 (s, 3H).

^{13}C NMR (101 MHz, $CDCl_3$) δ 194.8 (C=O), 163.9 (C), 139.9 (C), 135.8 (CH), 135.1 (C), 134.9 (C), 132.7 (C), 132.4 (C), 131.7 (CH), 130.8 (2 x CH), 130.6 (2 x CH), 129.7 (C), 129.5 (2 x CH), 128.5 (CH), 114.0 (2 x CH), 55.6 (CH_3), 43.3 (CH_2).

FTIR (neat) ν_{max}, cm^{-1}: 2934, 1681, 1600, 1475, 1262, 1221, 1170, 1091, 818.

HRMS (ESI+): calculated for $C_{21}H_{16}O_2SCl_2Na^+$ [$M+Na^+$] 425.0140, found: 425.0138.

2-(5-bromo-2-((4-bromophenyl)thio)phenyl)-1-(4-methoxyphenyl)ethan-1-one 2f

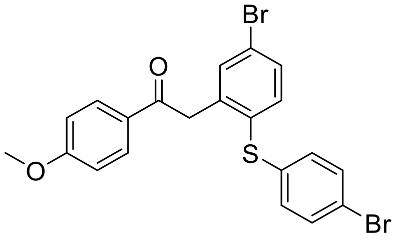

Following General procedure A1 using 4-methoxyacetophenone (0.030 g, 0.2 mmol), 2,6-di-*tert*-butyl-4-methylpyridine (0.0452 g, 0.22 mmol), trifluoromethanesulfonic acid anhydride (37 µL, 0.22 mmol) and 4,4'-sulfinylbis(bromobenzene) (0.216 g, 0.6 mmol) gave after 30 min of activation and purification by flash column chromatography (SiO_2, DCM-heptane 1:1, then 3:1, then 4:1) target compound **2f** as a white solid (0.073 g, 74%).

R_f 0.5 (heptane:EtOAc 7:3).

^{1}H NMR (600 MHz, $CDCl_3$) δ 7.95 – 7.90 (m, 2H), 7.47 (d, *J* = 2.1 Hz, 1H), 7.39 (dd, *J* = 8.3, 2.2 Hz, 1H), 7.34 – 7.30 (m, 2H), 7.29 – 7.25 (m, 1H), 7.00 – 6.96 (m, 2H), 6.95 – 6.89 (m, 2H), 4.38 (s, 2H, CH_2), 3.87 (s, 3H, OCH_3).

^{13}C NMR (151 MHz, $CDCl_3$) δ 194.8 (CO), 163.9 (Ar*C*), 140.2 (Ar*C*), 136.0 (Ar*C*H), 135.7 (Ar*C*), 134.6 (Ar*C*H), 133.0 (Ar*C*), 132.4 (2 x Ar*C*H), 131.5 (Ar*C*H), 130.9 (2 x Ar*C*H), 130.7 (2 x Ar*C*H), 129.7 (Ar*C*), 123.0 (Ar*C*), 120.7 (Ar*C*), 114.0 (2 x Ar*C*H), 55.7 (OCH_3), 43.3 (CH_2).

IR (neat) ν/cm^{-1}: 2930,1679, 1600, 1470, 1262, 1221, 1169, 1007, 810.

HRMS (ESI+): exact mass for $[M+Na]^+$ ($C_{21}H_{16}O_2SNaBr_2^+$) calculated: 512.9130, found: 512.9113.

2-(5-fluoro-2-((4-fluorophenyl)thio)phenyl)-1-(4-methoxyphenyl)ethan-1-one 2g

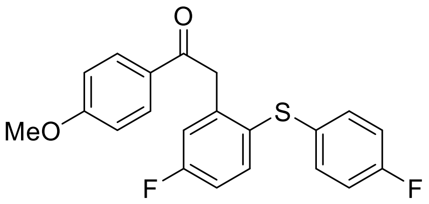

Following General procedure A1 using 4-methoxyacetophenone (0.030 g, 0.2 mmol), 2,6-di-*tert*-butyl-4-methylpyridine (0.0452 g, 0.22 mmol), trifluoromethanesulfonic acid anhydride (37 µL, 0.22 mmol) and 4,4'-sulfinylbis(fluorobenzene) (0.0953 g, 0.4 mmol) gave after 30 min of activation and purification by flash column chromatography (SiO_2, DCM-heptane 1:1, then 3:1, then 4:1) target compound **2g** as a yellow oil (0.060 g, 81%).

R_f 0.45 (heptane:EtOAc 7:3).

^{1}H NMR (600 MHz, $CDCl_3$) δ 7.98 – 7.89 (m, 2H), 7.42 (dd, *J* = 8.6, 5.7 Hz, 1H), 7.13 – 7.03 (m, 2H), 7.05 (dd, *J* = 9.3, 2.8 Hz, 1H), 6.98 (dd, *J* = 8.3, 2.9 Hz, 1H), 6.95 – 6.89 (m, 4H), 4.41 (s, 2H), 3.87 (s, 3H).

^{13}C NMR (101 MHz, $CDCl_3$) δ 195.0 (*C*=O), 163.8 (Ar*C*), 162.8 (d, $^1J_{CF}$ = 249.0 Hz, Ar*C*), 161.8 (d, ^1J = 246.7 Hz, Ar*C*), 140.6 (d, $^2J_{CF}$ = 8.2 Hz, Ar*C*), 136.6 (d, $^2J_{CF}$ = 8.4 Hz, Ar*C*H), 131.9 (d, J_{CF} = 2.3 Hz, Ar*C*), 131.2 (d, $^2J_{CF}$ = 8.0 Hz, 2 x Ar*C*H), 130.7 (2 x Ar*C*H), 129.7 (Ar*C*), 129.6 (d, J_{CF} = 3.3 Hz, Ar*C*), 118.6 (d, J_{CF} = 22.3 Hz, Ar*C*H), 116.4 (d, $^2J_{CF}$ = 22.1 Hz, 2 x Ar*C*H), 115.4 (d, J_{CF} = 21.6 Hz, Ar*C*H), 113.9 (2 x Ar*C*H), 55.6 (d, J_{CF} = 1.9 Hz, CH_3), 43.5 (CH_2).

^{19}F NMR (659 MHz, $CDCl_3$) δ -112.53, -115.51.

FTIR (neat) v_{max}, cm^{-1}: 2922, 1678, 1599, 1577, 1510, 1489, 1473, 1262, 1229, 1170, 826.

HRMS (ESI+): calculated for $C_{21}H_{16}F_2O_2SNa^+$ [M + Na^+] 393.0731; found 393.0724.

1-(4-methoxyphenyl)-2-(2-(methylthio)phenyl)ethan-1-one 2h

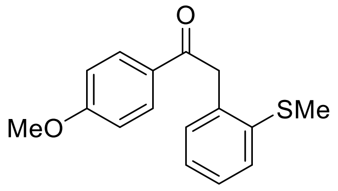

Following General procedure A1 using 4-methoxyacetophenone (0.030 g, 0.2 mmol), 2,6-di-*tert*-butyl-4-methylpyridine (0.0452 g, 0.22 mmol), trifluoromethanesulfonic acid anhydride (37 µL, 0.22 mmol) and (methylsulfinyl)benzene (0.0561 g, 0.4 mmol) gave after 30 min of activation and purification by flash column chromatography (SiO_2, heptane-EtOAc 19:1, then 14:1, then 9:1) target compound **2h** as a yellow solid (0.0218 g, 40%).

R_f 0.42 (heptane:EtOAc 7:3).

^{1}H NMR (400 MHz, $CDCl_3$) δ 8.08 – 8.00 (m, 2H), 7.36 – 7.30 (m, 1H), 7.30 – 7.23 (m, 1H), 7.21 – 7.11 (m, 2H), 6.99 – 6.90 (m, 2H), 4.39 (s, 2H), 3.87 (s, 3H), 2.44 (s, 3H).

^{13}C NMR (101 MHz, $CDCl_3$) δ 196.0 (*C*O), 163.7 (Ar*C*), 137.8 (Ar*C*), 134.5 (Ar*C*), 130.9 (2 x Ar*C*H), 130.6 (Ar*C*H), 130.1 (Ar*C*), 127.9 (Ar*C*H), 127.5 (Ar*C*H), 125.8 (Ar*C*H), 113.9 (2 x Ar*C*H), 55.8 (*C*H_3), 43.3 (*C*H_2), 16.8 (*C*H_3).

The data are in accordance with those reported previously.[3]

1-(3-fluoro-4-methoxyphenyl)-2-(2-(phenylthio)phenyl)ethan-1-one 2i

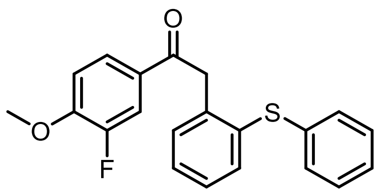

Following General procedure A1 using 3-fluoro-4-methoxyacetophenone (0.0336 g, 0.2 mmol), 2,6-di-*tert*-butyl-4-methylpyridine (0.0452 g, 0.22 mmol), trifluoromethanesulfonic acid anhydride (37 µL, 0.22 mmol) and diphenyl sulfoxide (0.0809 g, 0.4 mmol) gave after

120 min of activation and purification by flash column chromatography (SiO_2, DCM-heptane 1:1, then 3:1, then 4:1) target compound **2i** as a yellow oil (0.0486 g, 69%).

R_f 0.44 (heptane:EtOAc 7:3).

^{1}H NMR (400 MHz, $CDCl_3$) δ 7.67 (ddd, J = 8.5, 2.1, 1.1 Hz, 1H), 7.61 (dd, J = 11.9, 2.1 Hz, 1H), 7.41 – 7.35 (m, J = 7.4 Hz, 1H), 7.24 – 7.04 (m, 8H), 6.86 (t, J = 8.3 Hz, 1H), 4.32 (s, 2H), 3.86 (s, 3H).

^{13}C NMR (151 MHz, $CDCl_3$) δ 195.1 (d, $^4J_{CF}$ = 1.7 Hz, C=O), 152.1 (d, $^1J_{CF}$ = 247.9 Hz, Ar*C*), 152.0 (d, $^2J_{CF}$ = 10.9 Hz, Ar*C*), 137.7 (Ar*C*), 136.7 (Ar*C*), 134.8 (Ar*C*H), 134.0 (Ar*C*), 131.3 (Ar*C*H), 130.1 (d, $^3J_{CF}$ = 5.0 Hz, Ar*C*), 129.3 (2 x Ar*C*H), 129.3 (2 x Ar*C*H), 128.7 (Ar*C*H), 128.3 (Ar*C*H), 126.5 (Ar*C*H), 125.9 (d, $^3J_{CF}$ = 3.3 Hz, Ar*C*H), 116.1 (d, $^2J_{CF}$ = 19.0 Hz, Ar*C*H), 112.4 (d, J = 1.6 Hz, Ar*C*H), 56.4 (CH_2), 43.6 (CH_3).

^{19}F NMR (659 MHz, $CDCl_3$) δ -134.25.

FTIR (neat) ν_{max}, cm^{-1}: 2935, 1682, 1611, 1582, 1518, 1434, 1328, 1283, 1262, 1137, 1119, 1016, 743.

HRMS (ESI+): calculated for $C_{21}H_{17}FO_2SNa^+$ [M + Na^+] 375.0825; found 375.0822

1-(3-chloro-4-methoxyphenyl)-2-(2-(phenylthio)phenyl)ethan-1-one 2j

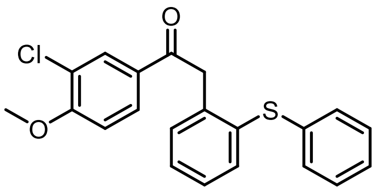

Following General procedure A1 using 3-chloro-4-methoxyacetophenone (0.0369 g, 0.2 mmol), 2,6-di-*tert*-butyl-4-methylpyridine (0.0452 g, 0.22 mmol), trifluoromethanesulfonic acid anhydride (37 µL, 0.22 mmol) and diphenyl sulfoxide (0.0809 g, 0.4 mmol) gave after 120 min of activation and purification by flash column chromatography (SiO_2, DCM-heptane 1:1, then 3:1, then 4:1) target compound **2j** as a white solid (0.0568 g, 77%).

R_f 0.38 (heptane:EtOAc 7:3).

^{1}H NMR (600 MHz, $CDCl_3$) δ 8.03 (d, J = 2.2 Hz, 1H), 7.90 (dd, J = 8.6, 2.2 Hz, 1H), 7.48 – 7.45 (m, 1H), 7.36 – 7.23 (m, 5H), 7.21 – 7.15 (m, 3H), 6.94 (d, J = 8.7 Hz, 1H), 4.42 (s, 2H), 3.97 (s, 3H).

^{13}C NMR (151 MHz, CDCl3) δ 195.0 (*C*=O), 158.9 (*C*), 137.6 (*C*), 136.7 (*C*), 134.7 (*C*H), 134.0 (*C*), 131.4 (*C*H), 130.8 (*C*H), 130.4 (*C*), 129.3 (2 x *C*H), 129.2 (2 x *C*H), 129.0 (*C*H), 128.7 (*C*H), 128.3 (*C*H), 126.5 (*C*H), 122.9 (*C*), 111.3 (*C*H), 56.5 (CH_2), 43.5 (CH_3).

FTIR (neat) ν_{max}, cm^{-1}: 3056, 1681, 1593, 1567, 1502, 1476, 1439, 1276, 1257, 1061, 1015, 742.

HRMS (ESI+): calculated for $C_{21}H_{17}ClO_2SNa^+$ [M + Na$^+$] 391.0530; found 391.0524.

1-(3-chloro-4-methoxyphenyl)-2-(5-methyl-2-(p-tolylthio)phenyl)ethan-1-one 2k

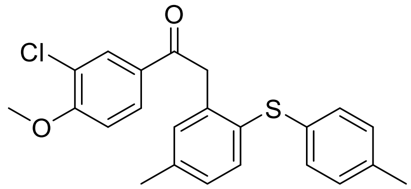

Following General procedure A1 using 3-chloro-4-methoxyacetophenone (0.0369 g, 0.2 mmol), 2,6-di-*tert*-butyl-4-methylpyridine (0.0452 g, 0.22 mmol), trifluoromethanesulfonic acid anhydride (37 µL, 0.22 mmol) and 4,4'-sulfinylbis(methylbenzene) (0.138 g, 0.6 mmol) gave after 120 min of activation and purification by flash column chromatography (SiO_2, DCM-heptane 1:1, then 3:1, then 4:1) target compound **2k** as a yellow oil (0.0556 g, 70%).

$\mathbf{R_f}$ 0.47 (heptane:EtOAc 7:3).

^{1}H NMR (600 MHz, $CDCl_3$) δ 7.99 (d, *J* = 2.2 Hz, 1H), 7.87 (dd, *J* = 8.6, 2.2 Hz, 1H), 7.34 (d, *J* = 7.9 Hz, 1H), 7.11 – 7.09 (m, *J* = 1.1 Hz, 1H), 7.06 (dd, *J* = 7.9, 1.4 Hz, 1H), 7.02 (br s, 4H), 6.91 (d, *J* = 8.7 Hz, 1H), 4.35 (s, 2H), 3.96 (s, 3H), 2.33 (s, 3H), 2.28 (s, 3H).

^{13}C NMR (151 MHz, $CDCl_3$) δ 195.3 (*C*=O), 158.8 (*C*), 138.7 (*C*), 137.4 (*C*), 136.3 (*C*), 134.8 (*C*H), 133.5 (*C*), 132.0 (*C*H), 130.9 (*C*H), 130.7 (*C*), 130.5 (*C*), 130.0 (2 x *C*H), 129.2 (2 x *C*H), 129.2 (*C*H), 129.1 (*C*H), 122.9 (*C*), 111.3 (*C*H), 56.5 (CH_3), 43.5 (CH_2), 21.3 (CH_3), 21.1 (CH_3).

FTIR (neat) ν_{max}, cm^{-1}: 3014, 2921, 2847, 1679, 1592, 1566, 1492, 1403, 1324, 1274, 1255, 1198, 1060, 1015, 803, 695.

HRMS (ESI+): calculated for $C_{23}H_{22}ClO_2S^+$ [M + H$^+$] 397.1024; found 397.1019.

1-(2,3-dihydrobenzo[b][1,4]dioxin-6-yl)-2-(2-(phenylthio)phenyl)ethan-1-one 2l

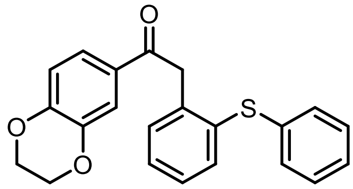

Following General procedure A1 using 1-(2,3-dihydrobenzo[b][1,4]dioxin-6-yl)ethan-1-one (0.0356 g, 0.2 mmol), 2,6-di-*tert*-butyl-4-methylpyridine (0.0452 g, 0.22 mmol), trifluoromethanesulfonic acid anhydride (37 µL, 0.22 mmol) and diphenyl sulfoxide (0.0809 g, 0.4 mmol) gave after 7 min of activation and purification by flash column chromatography (SiO_2, DCM-heptane 1:1, then 3:1, then 4:1) target compound as a yellow oil (0.0515 g, 71%).

R_f 0.22 (heptane:EtOAc 7:3).

^{1}H NMR (400 MHz, $CDCl_3$) δ 7.48 – 7.39 (m, 2H), 7.35 (d, *J* = 7.5 Hz, 1H), 7.24 – 7.04 (m, 8H), 6.78 (d, *J* = 8.4 Hz, 1H), 4.31 (s, 2H), 4.24 – 4.14 (m, *J* = 7.1, 2.8 Hz, 4H).

^{13}C NMR (101 MHz, $CDCl_3$) δ_C 195.8 (*C*O), 148.2 (*C*), 143.4 (*C*), 138.0 (*C*), 136.8 (*C*), 134.6 (*C*H), 134.2 (*C*), 131.3 (*C*H), 130.8 (*C*), 129.4 (2 x *C*H), 129.2 (2 x *C*H), 128.5 (*C*H), 128.1 (*C*H), 126.5 (*C*H), 122.7 (*C*H), 118.1 (*C*H), 117.3 (*C*H), 64.8 (R*C*H_2OR), 64.2 (R*C*H_2OR), 43.6 (*C*H_2).

FTIR (neat) ν_{max}, cm^{-1}: 3056, 2925, 1675, 1603, 1579, 1504, 1427, 1319, 1284, 1255, 1063, 895, 739, 670.

HRMS (ESI+): calculated for $C_{22}H_{19}O_3S^+$ [M + H$^+$] 363.1049; found 363.1047.

1-(2,3-dihydrobenzofuran-5-yl)-2-(2-(phenylthio)phenyl)ethan-1-one 2m

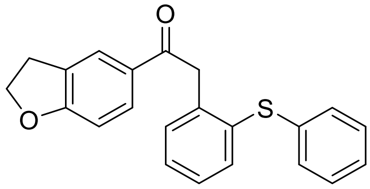

Following General procedure A1 using 1-(2,3-dihydrobenzofuran-5-yl)ethan-1-one (0.0324 g, 0.2 mmol), 2,6-di-*tert*-butyl-4-methylpyridine (0.0431 g, 0.22 mmol), trifluoromethanesulfonic acid anhydride (37 µL, 0.22 mmol) and diphenyl sulfoxide (0.0809 g, 0.4 mmol) gave after 5 min of activation and purification by flash column chromatography (SiO_2, DCM-heptane 1:1, then 3:1, then 4:1) target compound as a yellow oil (0.0263 g, 38%).

R$_f$ 0.44 (heptane:EtOAc 7:3).

^{1}H NMR (400 MHz, $CDCl_3$) δ 7.79 – 7.71 (m, 2H), 7.36 (d, *J* = 7.5 Hz, 1H), 7.24 – 7.04 (m, 8H), 6.68 (d, *J* = 8.4 Hz, 1H), 4.56 (t, *J* = 8.8 Hz, 2H), 4.33 (s, 2H), 3.13 (t, *J* = 8.7 Hz, 2H).

^{13}C NMR (101 MHz, $CDCl_3$) δ 195.8 (*C*O), 164.5 (*C*), 138.2 (*C*), 137.0 (*C*), 134.7 (*C*H), 134.0 (*C*), 131.3 (*C*H), 130.6 (*C*H), 130.3 (*C*), 129.3 (2 x *C*H), 129.2 (2 x *C*H), 128.6 (*C*H), 128.1 (*C*H), 127.8 (*C*), 126.4 (*C*H), 125.9 (*C*H), 109.1 (*C*H), 72.3 (*C*H_2), 43.6 (*C*H_2), 29.1 (*C*H_2).

FTIR (neat) ν_{max}, cm^{-1}: 3056, 2920, 2852. 1671, 1604, 1583, 1437, 1325, 1238, 1107, 1096, 979, 739, 689.

HRMS (ESI+): calculated for $C_{22}H_{19}O_2S^+$ [M + H^+] 347.1100; found 347.1097.

1-(3-methoxyphenyl)-2-(2-(phenylthio)phenyl)ethan-1-one 4a

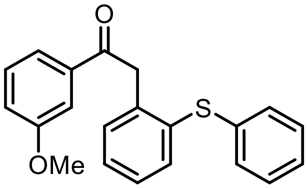

Following General procedure A2 using 3-methoxyacetophenone (27.6 µL, 0.2 mmol), 2,6-di-*tert*-butyl-4-methylpyridine (0.0452 g, 0.22 mmol), trifluoromethanesulfonic acid anhydride (37 µL, 0.22 mmol) and diphenyl sulfoxide (0.089 g, 0.4 mmol) gave after purification by flash column chromatography (SiO_2, DCM-heptane 1:1, then 3:1, then 4:1) target compound **4a** as a yellow oil (0.0401 g, 60%).

R$_f$ 0.5 (heptane:EtOAc 7:3).

^{1}H NMR (400 MHz, $CDCl_3$) δ_H 7.51 – 7.33 (m, 3H, 3 x Ar*H*), 7.29 – 6.98 (m, 10H, 10 x Ar*H*), 4.38 (s, 2H, C*H*$_2$), 3.75 (s, 3H, C*H*$_3$).

^{13}C NMR (101 MHz, $CDCl_3$) δ_C 197.2 (*C*=O), 159.9 (Ar*C*), 138.3 (Ar*C*), 137.8 (Ar*C*), 136.8 (Ar*C*), 134.7 (Ar*C*H), 134.2 (Ar*C*), 131.4 (Ar*C*H), 129.7 (2 x Ar*C*H), 129.4 (2 x Ar*C*H), 129.2 (Ar*C*H), 128.6 (Ar*C*H), 128.3 (Ar*C*H), 126.5 (Ar*C*H), 121.2 (Ar*C*H), 119.8 (Ar*C*H), 112.7 (Ar*C*H), 55.6 (O*C*H_3), 44.1 (*C*H_2).

HRMS (ESI+): calculated for $C_{21}H_{18}O_2S$ [M + H^+] 335.1100; found 335.1091

The data are in accordance with those reported previously.[3]

2-(5-chloro-2-((4-chlorophenyl)thio)phenyl)-1-(3-methoxyphenyl)ethan-1-one 4b

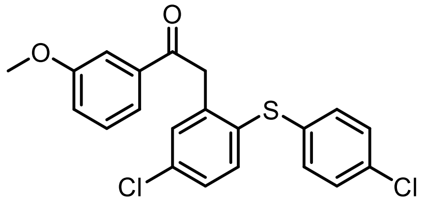

Following General procedure A2 using 3-methoxyacetophenone (27.6 µL, 0.2 mmol), 2,6-di-*tert*-butyl-4-methylpyridine (0.0452 g, 0.22 mmol), trifluoromethanesulfonic acid anhydride (37 µL, 0.22 mmol) and 4,4'-sulfinylbis(chlorobenzene) (0.108 g, 0.4 mmol) gave after purification by flash column chromatography (SiO_2, DCM-heptane 1:1, then 3:1, then 4:1) target compound **4b** as a yellow oil (0.0444 g, 55%).

R_f 0.57 (heptane:EtOAc 7:3).

^{1}H NMR (400 MHz, $CDCl_3$) δ_H 7.71 – 6.73 (m, 11H, 11 x Ar*H*), 4.34 (s, 2H, C*H*$_2$), 3.76 (s, 3H, C*H*$_3$).

^{13}C NMR (101 MHz, $CDCl_3$) δ_C 196.1 (*C*=O), 160.0 (*C*), 139.6 (*C*), 138.0 (*C*), 135.9 (*C*H), 135.0 (*C*), 134.9 (*C*), 132.8 (*C*), 132.5 (*C*), 131.7 (*C*H), 130.6 (2 x *C*H), 129.8 (*C*H), 129.5 (2 x *C*H), 128.6 (*C*H), 121.0 (*C*H), 120.0 (*C*H), 112.7 (*C*H), 55.6 (O*C*H$_3$), 43.8 (*C*H$_2$).

FTIR (neat) ν_{max}, cm^{-1}: 3050, 2921,1685, 1582, 1473, 1428, 1255, 1091, 1011, 814.

HRMS calculated for $C_{21}H_{16}Cl_2O_2NaS^+$ [M + Na$^+$] 425.0140; found 425.0142

1-(4-fluorophenyl)-2-(2-(phenylthio)phenyl)ethan-1-one 4c

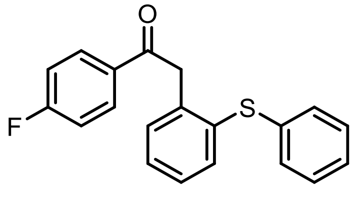

Following General procedure A2 using 4-fluoroacetophenone (24.4 µL, 0.2 mmol), 2,6-di-*tert*-butyl-4-methylpyridine (0.0452 g, 0.22 mmol), trifluoromethanesulfonic acid anhydride (37 µL, 0.22 mmol) and diphenyl sulfoxide (0.089 g, 0.4 mmol) gave after purification by flash column chromatography (SiO_2, DCM-heptane 1:1, then 3:1, then 4:1) target compound **4c** as a yellow oil (0.0400 g, 62%).

R_f 0.54 (heptane:EtOAc 7:3).

^{1}H NMR (400 MHz, $CDCl_3$) δ 7.93 – 7.86 (m, 2H), 7.40 – 7.34 (m, 1H), 7.26 – 6.96 (m, 10H), 4.36 (s, 2H).

^{13}C NMR (101 MHz, $CDCl_3$) δ_C 195.8 (*C*=O), 165.9 (d, $^1J_{CF}$ = 254.7 Hz, *C*), 137.6 (*C*), 136.7 (*C*), 134.9 (*C*H), 134.0 (*C*), 133.3 (d, $^4J_{CF}$ = 3.0 Hz, *C*), 131.4 (*C*H), 131.2 (d, $^3J_{CF}$ = 9.3 Hz, 2 x *C*H), 129.3 (2 x *C*H), 129.3 (2 x *C*H), 128.7 (*C*H), 128.4 (*C*H), 126.5 (*C*H), 115.8 (d, $^2J_{CF}$ = 21.9 Hz, 2 x *C*H), 43.8 (*C*H_2).

HRMS calculated for $C_{20}H_{15}FOSNa+$ [M + Na^+] 345.0720; found 345.0719

The data are in accordance with those reported previously.[3]

1-(4-fluorophenyl)-2-(5-methyl-2-(p-tolylthio)phenyl)ethan-1-one 4d

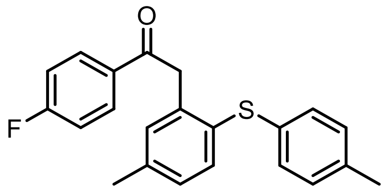

Following General procedure A2 using 4-fluoroacetophenone (24.4 µL, 0.2 mmol), 2,6-di-*tert*-butyl-4-methylpyridine (0.0452 g, 0.22 mmol), trifluoromethanesulfonic acid anhydride (37 µL, 0.22 mmol) and 4,4'-sulfinylbis(methylbenzene) (0.0921 g, 0.4 mmol) gave after purification by flash column chromatography (SiO_2, DCM-heptane 1:1, then 3:1, then 4:1) target compound **4d** as a yellow solid (0.0470 g, 67%).

R_f 0.62 (heptane:EtOAc 7:3).

^{1}H NMR (400 MHz, $CDCl_3$) δ 8.06 – 7.90 (m, 2H), 7.36 (d, *J* = 7.9 Hz, 1H), 7.13 – 6.99 (m, 8H), 4.40 (s, 2H, C*H_2*), 2.33 (s, 3H), 2.29 (s, 3H).

^{13}C NMR (101 MHz, $CDCl_3$) δ_C 196.0 (*C*O), 165.8 (d, *J* = 254.6 Hz, *C*), 138.7 (*C*), 137.4 (*C*), 136.3 (*C*), 134.9 (*C*H), 133.5 (*C*), 133.3 (d, *J* = 3.0 Hz, *C*), 132.0 (*C*H), 131.2 (d, *J* = 9.3 Hz, 2 x *C*H), 130.8 (*C*), 130.0 (2 x CH) 129.2 (2 x *C*H), 129.2 (C*H*), 115.7 (d, *J* = 21.9 Hz, 2 x *C*H), 43.7 (*C*H_2), 21.3 (*C*H_3), 21.1 (*C*H_3).

^{19}F NMR (659 MHz, $CDCl_3$) δ -105.4.

FTIR (neat) ν_{max}, cm^{-1}: 3055, 2924,1689, 1597, 1209, 1156, 805.

HRMS calculated for $C_{22}H_{20}FOS$ [M + H^+] 351.1213; found 351.1219.

2-(5-chloro-2-((4-chlorophenyl)thio)phenyl)-1-(4-fluorophenyl)ethan-1-one 4e

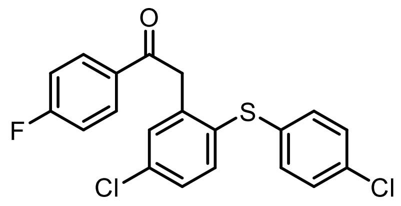

Following General procedure A2 using 4-fluoroacetophenone (24.4 µL, 0.2 mmol), 2,6-di-*tert*-butyl-4-methylpyridine (0.0452 g, 0.22 mmol), trifluoromethanesulfonic acid anhydride (37 µL, 0.22 mmol) and 4,4'-sulfinylbis(chlorobenzene) (0.108 g, 0.4 mmol) gave after purification by flash column chromatography (SiO_2, DCM-heptane 1:1, then 3:1, then 4:1) target compound **4e** as a yellow oil (0.0407 g, 52%).

R_f 0.65 (heptane:EtOAc 7:3).

^{1}H NMR (400 MHz, $CDCl_3$) δ_H 8.06 – 7.75 (m, 2H), 7.31 – 7.16 (m, 3H), 7.12 – 7.01 (m, 4H), 6.98 – 6.93 (m, 2H), 4.33 (s, 2H, CH_2).

^{13}C NMR (101 MHz, $CDCl_3$) δ_C 194.7 (*C*O), 166.1 (d, *J* = 255.4 Hz, *C*), 139.3 (*C*), 135.9 (*C*H), 135.0 (*C*), 134.9 (*C*), 133.0 (d, *J* = 3.0 Hz, *C*), 132.9 (*C*), 132.4 (*C*), 131.8 (*C*H), 131.1 (d, *J* = 9.4 Hz, 2 x *C*H), 130.6 (2 x *C*H), 129.5 (2 x *C*H), 128.7 (*C*H), 115.9 (d, *J* = 21.9 Hz, 2 x *C*H), 43.6 (*C*H_2).

^{19}F NMR (659 MHz, $CDCl_3$) δ -104.50.

HRMS calculated for $C_{20}H_{13}Cl_2FOSNa^+$ [M + Na^+] 412.9940; found 412.9937.

FTIR (neat) ν_{max}, cm^{-1}: 3050, 2918,1689,1597, 1474, 1215, 1156, 1092, 834, 816.

1-(4-chlorophenyl)-2-(2-(phenylthio)phenyl)ethan-1-one 4f

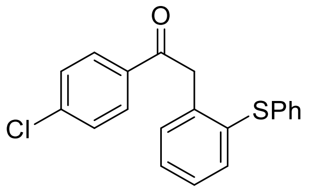

Following General procedure A2 using 4-chloroacetophenone (26 µL, 0.2 mmol), 2,6-di-*tert*-butyl-4-methylpyridine (0.0452 g, 0.22 mmol), trifluoromethanesulfonic acid anhydride (37 µL, 0.22 mmol) and diphenyl sulfoxide (0.121 g, 0.6 mmol) gave after purification by

flash column chromatography (SiO_2, heptane-EtOAc 19:1, then 14:1, then 9:1) target compound **4f** as a yellow oil (0.0509 g, 75%).
The data are in accordance with those reported previously.[3]

2-(5-chloro-2-((4-chlorophenyl)thio)phenyl)-1-(4-chlorophenyl)ethan-1-one 4g

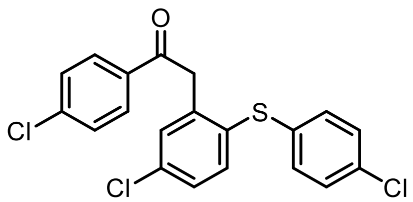

Following General procedure A2 using 4-chloroacetophenone (26 µL, 0.2 mmol), 2,6-di-*tert*-butyl-4-methylpyridine (0.0452 g, 0.22 mmol), trifluoromethanesulfonic acid anhydride (37 µL, 0.22 mmol) and 4,4'-sulfinylbis(chlorobenzene) (0.108 g, 0.4 mmol) gave after purification by flash column chromatography (SiO_2, DCM-heptane 1:1, then 3:1, then 4:1) target compound **4g** as a yellow oil (0.0424 g, 52%).
$\mathbf{R_f}$ 0.73 (heptane:EtOAc 7:3).

^{1}H NMR (400 MHz, $CDCl_3$) δ 7.90 – 7.84 (m, 2H), 7.46 – 7.40 (m, 2H), 7.39 – 7.34 (m, 1H), 7.31 (d, *J* = 2.2 Hz, 1H), 7.28 – 7-24 (m, 1H), 7.21 – 7.15 (m, 2H), 7.05 – 7.00 (m, 2H), 4.39 (s, 2H).

^{13}C NMR (101 MHz, $CDCl_3$) δ 195.1 (*C*O), 140.0 (*C*), 139.2 (*C*), 135.9 (*C*H), 134.9 (*C*), 134.8 (*C*), 134.0 (*C*), 132.9 (*C*), 132.4 (*C*), 131.8 (*C*H), 130.5 (2 x *C*H), 129.8 (2 x *C*H), 129.5 (*C*H), 129.1 (2 x *C*H), 128.8 (2 x *C*H), 43.7 (*C*H_2).

FTIR (neat) ν_{max}, cm^{-1}: 3049, 2922, 1688, 1588, 1474, 1213, 1091, 993, 814.

HRMS calculated for $C_{20}H_{13}Cl_3OSNa$+ [M + Na^+] 428.9645; found 428.9639

1-phenyl-2-(2-(phenylthio)phenyl)ethan-1-one 4h

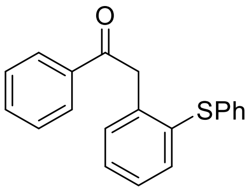

Following General procedure A2 using acetophenone (23.3 µL, 0.2 mmol), 2,6-di-*tert*-butyl-4-methylpyridine (0.0452 g, 0.22 mmol), trifluoromethanesulfonic acid anhydride (37 µL, 0.22 mmol) and diphenyl sulfoxide (0.121 g, 0.6 mmol) gave after purification by flash

column chromatography (SiO_2, heptane-EtOAc 19:1, then 14:1, then 9:1) target compound **4h** as a yellow oil (0.0516 g, 85%).

R$_f$ 0.47 (heptane:EtOAc 7:3).

^{1}H NMR (400 MHz, $CDCl_3$) δ 7.96 – 7.83 (m, 2H), 7.52 – 7.41 (m, 1H), 7.41 – 7.31 (m, 3H), 7.27 – 7.01 (m, 8H), 4.40 (s, 2H).

^{13}C NMR (101 MHz, $CDCl_3$) δ 197.3 (C=O), 137.8 (*C*), 136.9 (*C*), 136.8 (*C*), 134.8 (*C*H), 134.2 (*C*), 133.2 (*C*H), 131.4 (*C*H), 129.4 (2 x *C*H), 129.2 (2 x *C*H), 128.7 (2 x *C*H), 128.6 (CH), 128.5 (2 x *C*H), 128.3 (CH), 126.5 (CH), 44.0 (CH_2).

The data are in accordance with those reported in the literature.[3]

2-(5-methyl-2-(p-tolylthio)phenyl)-1-phenylethan-1-one 4i

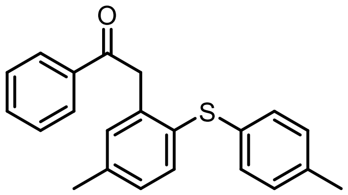

Following General procedure A2 using acetophenone (23.3 µL, 0.2 mmol), 2,6-di-*tert*-butyl-4-methylpyridine (0.0452 g, 0.22 mmol), trifluoromethanesulfonic acid anhydride (37 µL, 0.22 mmol) and 4,4'-sulfinylbis(methylbenzene) (0.0921 g, 0.4 mmol) gave after purification by flash column chromatography (SiO_2, DCM-heptane 1:1, then 3:1, then 4:1) target compound **4i** as a yellow solid (0.0399 g, 60%).

R$_f$ 0.63 (heptane:EtOAc 7:3).

^{1}H NMR (400 MHz, $CDCl_3$) δ 8.01 – 7.93 (m, 2H), 7.54 (ddd, *J* = 8.7, 4.7, 1.9 Hz, 1H), 7.45 – 7.40 (m, 2H), 7.35 (d, *J* = 7.9 Hz, 1H), 7.12 – 6.99 (m, 6H), 4.44 (s, 2H), 2.33 (s, 3H), 2.28 (s, 3H).

^{13}C NMR (101 MHz, $CDCl_3$) δ_C 197.6 (*C*O), 138.6 (*C*), 137.6 (*C*), 137.0 (*C*), 136.2 (*C*), 134.78 (Ar*C*H), 133.6 (*C*), 133.2 (*C*H), 132.1 (*C*H), 131.0 (Ar*C*), 130.0 (2 x *C*H), 129.3 (2 x *C*H), 129.1 (*C*H), 128.7 (2 x *C*H), 128.6 (2 x Ar*C*H), 43.9 (*C*H_2), 21.3 (*C*H_3), 21.1 (*C*H_3).

HRMS calculated for $C_{22}H_{20}OSNa^+$ [M + Na$^+$] 355.1127; found 355.1126.

The data are in accordance with those reported in the literature.[3]

2-(5-chloro-2-((4-chlorophenyl)thio)phenyl)-1-phenylethan-1-one 4j

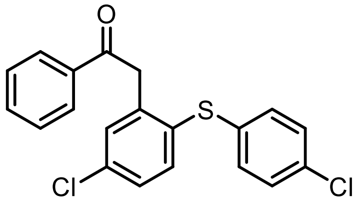

Following General procedure A2 using acetophenone (23.3 µL, 0.2 mmol), 2,6-di-*tert*-butyl-4-methylpyridine (0.0452 g, 0.22 mmol), trifluoromethanesulfonic acid anhydride (37 µL, 0.22 mmol) and 4,4'-sulfinylbis(chlorobenzene) (0.108 g, 0.4 mmol) gave after purification by flash column chromatography (SiO_2, DCM-heptane 1:1, then 3:1, then 4:1) target compound **4j** as a yellow oil (0.0299 g, 40%).

R_f 0.66 (heptane:EtOAc 7:3).

^{1}H NMR (400 MHz, $CDCl_3$) δ 7.98 – 7.91 (m, 2H), 7.61 – 7.55 (m, 1H), 7.49 – 7.43 (m, 2H), 7.38 – 7.30 (m, 2H), 7.27 – 7.23 (m, 1H), 7.20 – 7.15 (m, 2H), 7.07 – 7.02 (m, 2H), 4.44 (s, 2H).

^{13}C NMR (101 MHz, $CDCl_3$) δ_C 196.3 (*C*=O), 139.6 (*C*), 136.6 (*C*), 135.9 (*C*H), 135.0 (*C*), 134.9 (*C*), 133.5 (*C*H), 132.8 (*C*), 132.5 (*C*), 131.8 (*C*H), 130.6 (2 x *C*H), 129.5 (2 x *C*H), 128.8 (2 x *C*H), 128.6 (*C*H), 128.4 (2 x *C*H), 43.7 (*C*H_2).

HRMS calculated for $C_{20}H_{15}Cl_2OS$ [M + H^+] 373.0215; found 373.0198

The data are in accordance with those reported in the literature.[3]

1-(4-methoxyphenyl)-2-(2-(phenylthio)phenyl)propan-1-one 7a

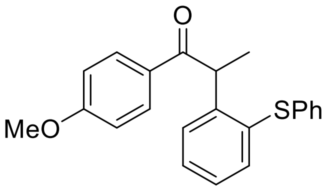

Following General procedure B1 using 1-(4-methoxyphenyl)propan-1-one (30.7 µL, 0.2 mmol), 2,6-di-*tert*-butyl-4-methylpyridine (0.0452 g, 0.22 mmol), trifluoromethanesulfonic acid anhydride (37 µL, 0.22 mmol) and diphenyl sulfoxide (0.0809 g, 0.4 mmol) gave after purification by flash column chromatography (SiO_2, toluene-DCM 4:1, then 2:1) target compound **7a** as a yellow solid (0.0290 g, 40%).

R_f 0.54 (heptane:EtOAc 7:3).

^{1}H NMR (400 MHz, $CDCl_3$) δ 7.87 – 7.77 (m, 2H), 7.36 – 7.29 (m, 1H), 7.24 – 7.04 (m, 8H), 6.72 – 6.64 (m, 2H), 5.23 (q, *J* = 6.8 Hz, 1H), 3.72 (s, 3H), 1.31 (d, *J* = 6.8 Hz, 3H).

^{13}C NMR (101 MHz, $CDCl_3$) δ 199.3 (*C*O), 163.3 (*C*), 144.2 (*C*), 136.5 (*C*), 134.8 (*C*H), 132.7 (*C*), 131.2 (2 x *C*H), 130.0 (2 x *C*H), 129.5 (*C*), 129.4 (2 x *C*H), 129.0 (*C*H), 128.3 (*C*H), 127.8 (*C*H), 126.8 (*C*H), 113.8 (2 x *C*H), 55.5 (O*C*H_3), 44.6 (*C*H), 18.9 (*C*H_3).

FTIR (neat) ν_{max}, cm^{-1}: 2963, 2929, 1673, 1598, 1509, 1467, 1242, 1168, 1025, 844, 731, 690.

HRMS calculated for $C_{22}H_{20}O_2SNa^+$ [M + Na$^+$] 371.1076; found 371.1074

1-(4-methoxyphenyl)-2-(2-(methylthio)phenyl)propan-1-one 7b

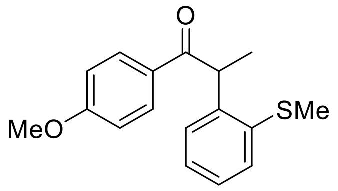

Following General procedure B1 using 1-(4-methoxyphenyl)propan-1-one (30.7 µL, 0.2 mmol), 2,6-di-*tert*-butyl-4-methylpyridine (0.0452 g, 0.22 mmol), trifluoromethanesulfonic acid anhydride (37 µL, 0.22 mmol) and (methylsulfinyl)benzene (0.0841 g, 0.6 mmol) gave after purification by flash column chromatography (SiO_2, toluene-DCM 4:1, then 2:1) target compound **7b** a yellow solid (0.020 g, 35%).

R_f 0.49 (heptane:EtOAc 7:3).

^{1}H NMR (400 MHz, $CDCl_3$) δ 7.91 – 7.81 (m, 2H), 7.23 – 7.15 (m, 1H), 7.10 (ddd, *J* = 7.9, 6.7, 2.1 Hz, 1H), 7.01 – 6.93 (m, *J* = 7.6, 1.6 Hz, 2H), 6.79 – 6.72 (m, 2H), 4.98 (q, *J* = 6.8 Hz, 1H), 3.72 (s, 3H), 2.47 (s, 3H), 1.38 (d, *J* = 6.8 Hz, 3H).

^{13}C NMR (101 MHz, $CDCl_3$) δ 199.2 (*C*O), 163.4 (*C*), 140.5 (*C*), 136.1 (*C*), 131.1 (2 x *C*H), 129.5 (*C*), 127.6 (*C*H), 127.4 (*C*H), 126.6 (*C*H), 125.9 (*C*H), 113.8 (2 x *C*H), 55.5 (O*C*H_3), 44.1 (C*H*), 18.2 (*C*H_3), 16.4 (*C*H_3).

FTIR (neat) ν_{max}, cm^{-1}: 2970, 2927, 1672, 1598, 1509, 1246, 1226, 1168, 1029, 949. 844, 749.

HRMS calculated for $C_{17}H_{18}O_2SNa^+$ [M + Na$^+$] 309.0920; found 309.0911.

1-phenyl-2-(2-(phenylthio)phenyl)propan-1-one 8a

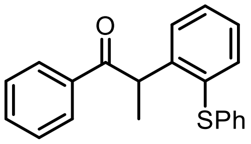

Following General procedure B2 using propiophenone (66.5 µL, 0.5 mmol), 2,6-di-*tert*-butyl-4-methylpyridine (0.108 g, 0.525 mmol), trifluoromethanesulfonic acid anhydride (92.5 µL, 0.55 mmol) and diphenyl sulfoxide (0.202 g, 1.0 mmol) gave after purification by flash column chromatography (SiO_2, toluene-DCM 4:1, then 2:1) target compound **8a** as a yellow oil (0.072 g, 45%).

$\mathbf{R_f}$ 0.68 (heptane:EtOAc 7:3).

^{1}H NMR (400 MHz, $CDCl_3$) δ 7.85 – 7.78 (m, 2H), 7.37 – 7.30 (m, 2H), 7.23 – 7.03 (m, 10H), 5.27 (q, *J* = 6.8 Hz, 1H), 1.32 (d, *J* = 6.8 Hz, 3H).

^{13}C NMR (101 MHz, $CDCl_3$) δ 200.7 (*C*O), 143.7 (*C*), 136.4 (*C*), 136.4 (*C*), 134.9 (*C*H), 132.9 (*C*), 132.9 (*C*H), 129.9 (2 x *C*H), 129.4 (2 x *C*H), 129.0 (*C*H), 128.8 (2 x *C*H), 128.5 (2 x *C*H), 128.3 (*C*H), 127.9 (*C*H), 126.9 (*C*H), 44.9 (*C*H), 18.7 (*C*H_3).

FTIR (neat) ν_{max}, cm^{-1}: 3058, 2929, 1681, 1580, 1470, 1438, 1219, 950, 737, 687.

HRMS calculated for $C_{21}H_{18}OSNa^+$ [M + Na^+] 341.0971; found 341.0974.

1-phenyl-2-(2-(phenylthio)phenyl)pentan-1-one 8b

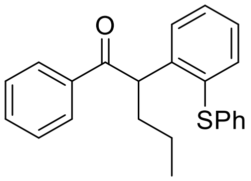

Following General procedure B2 using 1-phenylpentan-1-one (82.8 µL, 0.5 mmol), 2,6-di-*tert*-butyl-4-methylpyridine (0.108 g, 0.525 mmol), trifluoromethanesulfonic acid anhydride (92.5 µL, 0.55 mmol) and diphenyl sulfoxide (0.202 g, 1.0 mmol) gave after purification by flash column chromatography (SiO_2, toluene-DCM 4:1, then 2:1) target compound **8b** as a yellow solid (0.103 g, 60%).

$\mathbf{R_f}$ 0.76 (heptane:EtOAc 7:3).

^{1}H NMR (400 MHz, $CDCl_3$) δ 7.86 (d, *J* = 7.8 Hz, 2H), 7.34 – 7.25 (m, 2H), 7.23 – 6.99 (m, 10H), 5.25 (t, *J* = 6.9 Hz, 1H), 2.13 – 1.96 (m, 1H, CH_AH_B), 1.57 – 1.41 (m, 1H, CH_AH_B), 1.32 – 1.18 (m, 1H, CH_AH_B), 1.18 – 1.00 (m, 1H, CH_AH_B), 0.75 (t, *J* = 7.3 Hz, 3H, CH_3).

^{13}C NMR (101 MHz, $CDCl_3$) δ 200.3 (*C*O), 142.1 (*C*), 137.0 (*C*), 136.5 (*C*), 134.7 (*C*H), 133.5 (*C*), 132.9 (*C*H), 129.9 (2 x *C*H), 129.3 (2 x *C*H), 128.8 (2 x *C*H), 128.7 (*C*H), 128.6 (2 x *C*H), 128.5 (*C*H), 127.8 (*C*H), 126.8 (*C*H), 49.7 (*C*H), 36.4 (CH_2), 21.2 (CH_2), 14.2 (CH_3).

FTIR (neat) ν_{max}, cm^{-1}: 3058, 2957, 2929, 2870, 1680, 1580, 1465, 1439, 1208, 1055, 736, 687.

HRMS calculated for $C_{23}H_{23}OS^+$ [M + H^+] 347.1464; found 347.1450.

1.7.2 Alpha-aminoxylation of ketones

1-(4-methoxyphenyl)-2-((2,2,6,6-tetramethylpiperidin-1-yl)oxy)ethan-1-one 10a

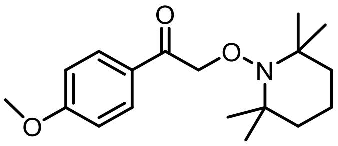

Following General procedure C using 4-methoxyacetophenone (30.0 mg, 0.2 mmol), 2,6-di-*tert*-butyl-4-methylpyridine (0.0431 g, 0.21 mmol), trifluoromethanesulfonic acid anhydride (37 µL, 0.22 mmol) and TEMPO (0.0957 g, 0.6 mmol) gave after 30 min of activation (addition of triflic anhydride at 0 °C) and purification by flash column chromatography (SiO_2, heptane-EtOAc 19:1, then 9:1) target compound **10a** as a white solid (0.034 g, 55%).

R_f 0.36 (heptane:EtOAc 4:1).

^{1}H NMR (400 MHz, $CDCl_3$) δ 8.05 – 7.85 (m, 2H), 7.00 – 6.87 (m, 2H), 5.04 (s, 2H, CH_2), 3.87 (s, 3H, OCH_3), 1.69 – 1.26 (m, 6H, 3 x CH_2), 1.18 (s, 12H, 4 x CH_3).

^{13}C NMR (101 MHz, $CDCl_3$) δ 194.5 (*C*=O), 163.7 (*C*), 130.5 (2 x *C*H), 128.7 (*C*), 113.8 (2 x *C*H), 81.4 (CH_2), 60.2 (2 x *C*), 55.5 (CH_3), 39.8 (2 x CH_2), 32.9 (2 x CH_3), 20.4 (2 x CH_3), 17.2 (CH_2).

FTIR (neat) ν_{max}, cm^{-1}: 3003, 2973, 2933, 1686, 1599, 1514, 1241, 1171, 1093, 972, 840.

HRMS calculated for $C_{18}H_{28}NO_3^+$ [M + H^+] 306.2064; found 306.3063.

1-(3-chloro-4-methoxyphenyl)-2-((2,2,6,6-tetramethylpiperidin-1-yl)oxy)ethan-1-one 10b

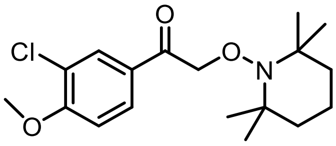

Following General procedure C using 3-chloro-4-methoxyacetophenone (36.9 mg, 0.2 mmol), 2,6-di-*tert*-butyl-4-methylpyridine (0.0431 g, 0.21 mmol), trifluoromethanesulfonic acid anhydride (37 µL, 0.22 mmol) and TEMPO (0.0957 g, 0.6 mmol) gave after activation for 115 min (addition of triflic anhydride at 0 °C) and purification by flash column chromatography (SiO_2, heptane-EtOAc 19:1, then 9:1) target compound **1-b** as a yellow solid (0.0347 g, 51%).

R_f 0.28 (heptane:EtOAc 4:1).

^{1}H NMR (400 MHz, $CDCl_3$) δ 8.02 (d, *J* = 2.1 Hz, 1H), 7.90 (dd, *J* = 8.6, 2.1 Hz, 1H), 7.04 – 6.92 (m, 1H), 5.00 (s, 2H, CH_2), 3.97 (s, 3H, OCH_3), 1.62 – 1.30 (m, 6H, 3 x CH_2), 1.17 (s, 12H, 4 x CH_3).

^{13}C NMR (101 MHz, $CDCl_3$) δ 193.8 (*C*=O), 159.0 (*C*), 130.7 (*C*H), 129.2 (*C*), 128.8 (*C*H), 122.9 (*C*), 111.4 (*C*H), 81.6 (CH_2), 60.3 (2 x *C*), 56.5 (OCH_3), 39.8 (2 x CH_2), 33.0 (2 x CH_3), 20.4 (2 x CH_3), 17.1 (CH_2).

FTIR (neat) v_{max}, cm^{-1}: 2971, 2933, 1681, 1595, 1499, 1263, 1217, 1132, 1059, 1011, 811, 707.

HRMS calculated for $C_{18}H_{27}ClNO_3^+$ [M + H^+] 340.1674; found 340.1658.

1-(3-fluoro-4-methoxyphenyl)-2-((2,2,6,6-tetramethylpiperidin-1-yl)oxy)ethan-1-one 10c

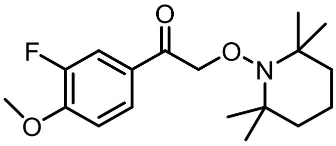

Following General procedure C using 3-fluoro-4-methoxyacetophenone (33.6 mg, 0.2 mmol), 2,6-di-*tert*-butyl-4-methylpyridine (0.0431 g, 0.21 mmol), trifluoromethanesulfonic acid anhydride (37 µL, 0.22 mmol) and TEMPO (0.0957 g, 0.6 mmol) gave after activation for 115 min (addition of triflic anhydride at 0 °C) and purification by flash column

chromatography (SiO_2, heptane-EtOAc 19:1, then 9:1) target compound **10c**as a white solid (0.0304 g, 47%).

R_f 0.24 (heptane:EtOAc 4:1).

^{1}H NMR (400 MHz, $CDCl_3$) δ 7.81 – 7.66 (m, 2H, Ar*H*), 7.04 – 6.95 (m, 1H, Ar*H*), 5.00 (s, 2H, C*H*$_2$), 3.95 (s, 3H, OC*H*$_3$), 1.67 – 1.26 (m, 6H, 3 x CH_2), 1.16 (s, 12H, 4 x C*H*$_3$).

^{13}C NMR (101 MHz, $CDCl_3$) δ 193.9 (d, $^4J_{CF}$ = 1.8 Hz, *C*=O), 152.1 (2 signals, d, $^1J_{CF}$ = 247.8 Hz, d, $^2J_{CF}$ = 11.3 Hz, 2 x *C*), 128.9 (d, $^3J_{CF}$ = 5.1 Hz, *C*), 125.6 (d, $^3J_{CF}$ = 3.4 Hz, *C*H), 116.1 (d, $^2J_{CF}$ = 19.2 Hz, *C*H), 112.5 (d, $^4J_{CF}$ = 1.6 Hz, *C*H), 81.6 (*C*H$_2$), 60.3 (2 x *C*), 56.4 (*C*H$_3$), 39.9 (2 x *C*H$_2$), 33.0 (2 x *C*H$_3$), 20.4 (2 x *C*H$_3$), 17.2 (*C*H$_2$).

^{19}F NMR (565 MHz, $CDCl_3$) δ -134.25.

FTIR (neat) ν_{max}, cm^{-1}: 2973, 2930, 1696, 1611, 1582, 1517, 1434, 1280, 1131, 814, 734.

HRMS calculated for $C_{18}H_{27}FNO_3^+$ [M + H$^+$] 324.1969; found 324.1975.

1-(2,3-dihydrobenzo[b][1,4]dioxin-6-yl)-2-((2,2,6,6-tetramethylpiperidin-1-yl)oxy)ethan-1-one 10d

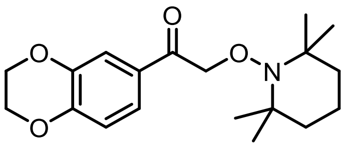

Following General procedure C using 1-(2,3-dihydrobenzo[b][1,4]dioxin-6-yl)ethan-1-one (35.6 mg, 0.2 mmol), 2,6-di-*tert*-butyl-4-methylpyridine (0.0431 g, 0.21 mmol), trifluoromethanesulfonic acid anhydride (37 µL, 0.22 mmol) and TEMPO (0.0957 g, 0.6 mmol) gave after activation for 8 min (addition of triflic anhydride at 0 °C) and purification by flash column chromatography (SiO_2, heptane-EtOAc 19:1, then 9:1) target compound **10d** as a white solid (0.0340 g, 51%).

R_f 0.28 (heptane:EtOAc 4:1).

^{1}H NMR (400 MHz, $CDCl_3$) δ 7.56 – 7.45 (m, 2H), 6.98 – 6.81 (m, 1H), 5.02 (s, 2H, C*H*$_2$), 4.33 – 4.25 (m, 4H, 2 x C*H*$_2$), 1.67 – 1.24 (m, 6H, 3 x CH_2), 1.17 (s, 12H, 4 x C*H*$_3$).

^{13}C NMR (101 MHz, $CDCl_3$) δ 194.3 (*C*=O), 148.2 (*C*), 143.5 (*C*), 129.4 (*C*), 122.3 (*C*H), 117.7 (*C*H), 117.4 (*C*H), 81.4 (*C*H$_2$), 64.8 (*C*H$_2$), 64.3 (*C*H$_2$), 60.3 (2 x *C*), 39.9 (2 x *C*H$_2$) , 33.0 (2 x *C*H$_3$), 20.4 (2 x *C*H$_3$), 17.2 (*C*H$_2$).

FTIR (neat) v_{max}, cm^{-1}: 2974. 2930, 1693, 1607, 1581, 1461, 1431, 1287, 1260, 1066.

HRMS calculated for $C_{19}H_{28}NO_4^+$ [M + H^+] 334.2013; found 334.2011.

1-(2,3-dihydrobenzofuran-5-yl)-2-((2,2,6,6-tetramethylpiperidin-1-yl)oxy)ethan-1-one 10e

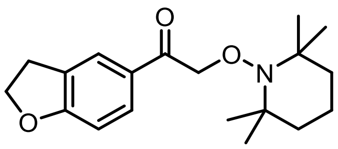

Following General procedure C using 1-(2,3-dihydrobenzofuran-5-yl)ethan-1-one (32.4 mg, 0.2 mmol), 2,6-di-*tert*-butyl-4-methylpyridine (0.0431 g, 0.21 mmol), trifluoromethanesulfonic acid (37 µL, 0.22 mmol) and TEMPO (0.0957 g, 0.6 mmol) gave after activation for 2 min (addition of triflic anhydride at 0 °C) and purification by flash column chromatography (SiO_2, heptane-EtOAc 19:1, then 9:1) target compound **10e**a white solid (0.0095 g, 15%).

R_f 0.30 (heptane:EtOAc 4:1).

^{1}H NMR (600 MHz, $CDCl_3$) δ 7.87 – 7.83 (m, 1H), 7.82 – 7.78 (m, 1H), 6.80 (d, *J* = 8.4 Hz, 1H), 5.03 (s, 2H, CH_2), 4.66 (t, *J* = 8.8 Hz, 2H, CH_2), 3.25 (t, *J* = 8.7 Hz, 2H, CH_2), 1.65 – 1.25 (m, 6H, 3 x CH_2), 1.17 (s, 12H, 4 x CH_3).

^{13}C NMR (151 MHz, $CDCl_3$) δ 194.4 (*C*=O), 164.6 (*C*), 130.1 (*C*H), 129.0 (*C*), 127.8 (*C*), 125.5 (*C*H), 109.2 (*C*H), 81.4 (*C*H_2), 72.3 (*C*H_2), 60.2 (2 x *C*), 39.9 (2 x *C*H_2), 33.0 (2 x *C*H_3), 29.2 (*C*H_2), 20.4 (2 x *C*H_3), 17.2 (*C*H_2).

FTIR (neat) v_{max}, cm^{-1}: 2967, 2927, 1690, 1607, 1591, 1493, 1441, 1084, 982, 942.

HRMS calculated for $C_{19}H_{28}NO_3$ [M + H^+] 318.2064; found 318.2054.

2-((2,2,6,6-tetramethylpiperidin-1-yl)oxy)-1-(3,4,5-trimethoxyphenyl)ethan-1-one 10f

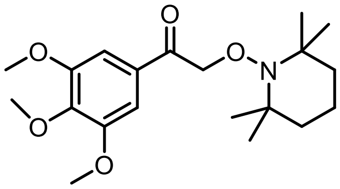

Following General procedure C using 1-(3,4,5-trimethoxyphenyl)ethan-1-one (42.0 mg, 0.2 mmol), 2,6-di-*tert*-butyl-4-methylpyridine (0.0431 g, 0.21 mmol), trifluoromethanesulfonic

acid anhydride (37 µL, 0.22 mmol) and TEMPO (0.0957 g, 0.6 mmol) gave after activation for 15 min (addition of triflic anhydride at 0 °C) and purification by flash column chromatography (SiO_2, heptane-EtOAc 19:1, then 9:1) target compound **10f** as a white solid (0.0146 g, 20%).

R$_f$ 0.33 (heptane:EtOAc 4:1).

^{1}H NMR (400 MHz, $CDCl_3$) δ 7.19 (s, 2H), 4.95 (s, 2H, CH_2), 3.85 (2 x s, 9H, 3 x OCH_3), 1.64 – 1.20 (m, 6H, 3 x CH_2), 1.16 – 1.08 (m, 12H, 4 x CH_3).

^{13}C NMR (101 MHz, $CDCl_3$) δ 194.8 (*C*=O), 153.2 (2 x *C*), 142.9 (*C*), 130.8 (*C*), 106.0 (2 x *C*H), 81.8 (*C*H_2), 60.4 (2 x *C*), 56.5 (3 x *C*H_3), 39.9 (2 x *C*H_2), 33.0 (2 x *C*H_3), 20.5 (2 x *C*H_3), 17.2 (*C*H_2).

FTIR (neat) ν_{max}, cm^{-1}: 2973, 2933, 1696, 1679, 1585, 1504, 1461, 1414, 1321, 1234, 1128, 1005

HRMS calculated for $C_{20}H_{32}NO_5$ [M + H$^+$] 366.2275; found 366.2264.

1.8 Formation of vinylsulfonium salts

1-(1-(4-methoxyphenyl)vinyl)tetrahydro-1*H*-thiophen-1-ium triflate 11

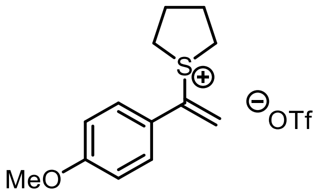

To a solution of 4-methoxyacetophenone (0.751 g, 5.0 mmol), 2,6-di-tert-butyl-4-methylpyridine (1.078 g, 5.25 mmol) and in anhydrous DCM (50 mL) was added dropwise at 0 °C trifluoromethanesulfonic acid anhydride (0.93 mL, 5.5 mmol). The reaction mixture was stirred at rt for 30 min. Then the reaction mixture was filtered over pentane (approx. 25 mL). Tetrahydrothiophene (0.44 mL, 5 mmol) was added and reaction mixture was stirred for 1.5 h at rt. Pentane and DCM were evaporated *in vacuo*. The residue was dissolved in Et_2O, and the organic phase was extracted with distilled water 3x. The aqueous layer was then extracted with DCM 3x. The organic layer filtered over sodium sulfate and evaporated *in vacuo* to give the product as a brown oil (1.19 g, 64%).

^{1}H NMR (600 MHz, $CDCl_3$) δ 7.43 – 7.34 (m, 2H, 2 x Ar*H*), 7.03 – 6.92 (m, 2H, 2 x Ar*H*), 6.14 (d, *J* = 3.3 Hz, 1H, C=CH_AH_B), 6.10 (d, *J* = 3.3 Hz, 1H, C=CH_AH_B), 4.04 – 3.94 (m, 2H, CH_2), 3.85 (s, 3H, OCH_3), 3.55 – 3.48 (m, 2H, CH_2), 2.46 – 2.37 (m, 2H, CH_2), 2.24 – 2.17 (m, 2H, CH_2).

^{13}C NMR (151 MHz, $CDCl_3$) δ 161.9 (Ar*C*), 135.4 (Ar*C*), 129.7 (2 x Ar*C*H), 125.1 (C=*C*H_2), 124.2 (Ar-*C*=CH_2), 120.8 (q, *J* = 320.3 Hz, *C*F_3), 115.3 (2 x Ar*C*H), 55.7 (O*C*H_3), 45.2 (2 x *C*H_2), 28.5 (2 x *C*H_2).

^{19}F NMR (565 MHz, $CDCl_3$) δ -78.27.

FTIR (neat) ν_{max}, cm^{-1}: 2973, 2933, 1696, 1679, 1585, 1504, 1461, 1414, 1321, 1234, 1128, 1005.

HRMS calculated for $C_{13}H_{17}OS^+$ [M^+] 221.0995; found 221.0997.

***N*-(2-hydroxyethyl)-4-methylbenzenesulfonamide 12**

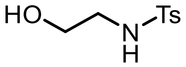

Literature procedure was followed.[4] *para*-Toluenesulfonyl chloride (1.0 g, 5.5 mmol) was taken up in dichloromethane (12 mL). Ethanolamine (0.30 mL, 5.0 mmol) was added and the mixture was cooled to 0 °C. Triethylamine (0.77 mL, 5.5 mmol) was added dropwise and the reaction was stirred at RT (18 h). The reaction mixture was diluted with dichloromethane and poured onto water. The aqueous phase was extracted with dichloromethane and the organic extracts were combined and dried over Na_2SO_4. The solvent was removed and the crude yellow oil of target compound **12** (1.07 g, 100%) was deemed to be of sufficient purity to be used without further purification.

The data are in accordance with the literature data.[4]

2-(4-methoxyphenyl)-4-tosylmorpholine 13

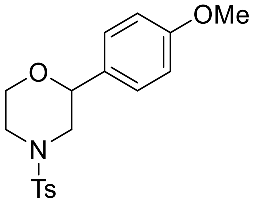

Modified literature procedure was applied.[5] A suspension of *N*-(2-hydroxyethyl)-4-methylbenzenesulfonamide **12** (0.0431 g, 0.2 mmol) and cesium carbonate (0.228 g, 0.7

mmol) in DCM (1 mL) was stirred at room temperature for 15 min. Then a solution of 1-(1-(4-methoxyphenyl)vinyl)tetrahydro-1*H*-thiophen-1-ium triflate (0.148 g, 0.4 mmol) in DCM (1 mL) was added dropwise to the solution. The reaction mixture was stirred at room temperature for 24 h. Then distilled water was added and the reaction mixture was extracted with DCM (3x), dried over sodium sulfate and evaporated *in vacuo*. Purification by flash column chromatography (SiO_2, heptane-EtOAc 19:1, then 9:1, then 4:1) gave the target compound **13** as a yellow oil (0.036 g, 52%).

^{1}H NMR (400 MHz, $CDCl_3$) δ 7.62 (d, *J* = 8.3 Hz, 2H), 7.32 (d, *J* = 8.0 Hz, 2H), 7.24 – 7.19 (m, 2H), 6.90 – 6.82 (m, 2H), 4.53 (dd, *J* = 10.3, 2.5 Hz, 1H), 4.05 (ddd, *J* = 11.5, 3.2, 1.2 Hz, 1H), 3.87 – 3.80 (m, 1H), 3.79 (s, 3H), 3.74 – 3.69 (m, 1H), 3.64 – 3.58 (m, 1H), 2.49 (tt, *J* = 7.7, 3.8 Hz, 1H), 2.43 (s, 3H), 2.24 (dd, *J* = 11.5, 10.4 Hz, 1H). The regioselectivity of the reaction was confirmed by ^{13}C DEPT and ^{13}C/HSQC NMR by looking at chemical shifts of the benzylic CH signals (expected shift of N*C*HPh 50-65 ppm vs. O*C*HPh 65-80 ppm).[5]

^{13}C NMR (101 MHz, $CDCl_3$) δ 159.7 (*C*O), 144.1 (*C*), 132.4 (*C*), 131.0 (*C*), 129.9 (2 x *C*H), 128.0 (2 x *C*H), 127.5 (2 x *C*H), 114.0 (2 x *C*H), 77.2 (*C*H, cross peak in ^{13}C/HSQC NMR with ^{1}H at 4.53 ppm), 66.3 (*C*H_2), 55.4 (*C*H_3), 52.1 (*C*H_2), 45.5 (*C*H_2), 21.7 (*C*H_3).

FTIR (neat) v_{max}, cm^{-1}: 2962, 2856, 1613, 1515, 1350, 1249, 1166, 966, 743.

HRMS calculated for $C_{18}H_{21}NO_4SNa^+$ [M + Na^+] 370.1083; found 370.1036.

2 NMR printouts

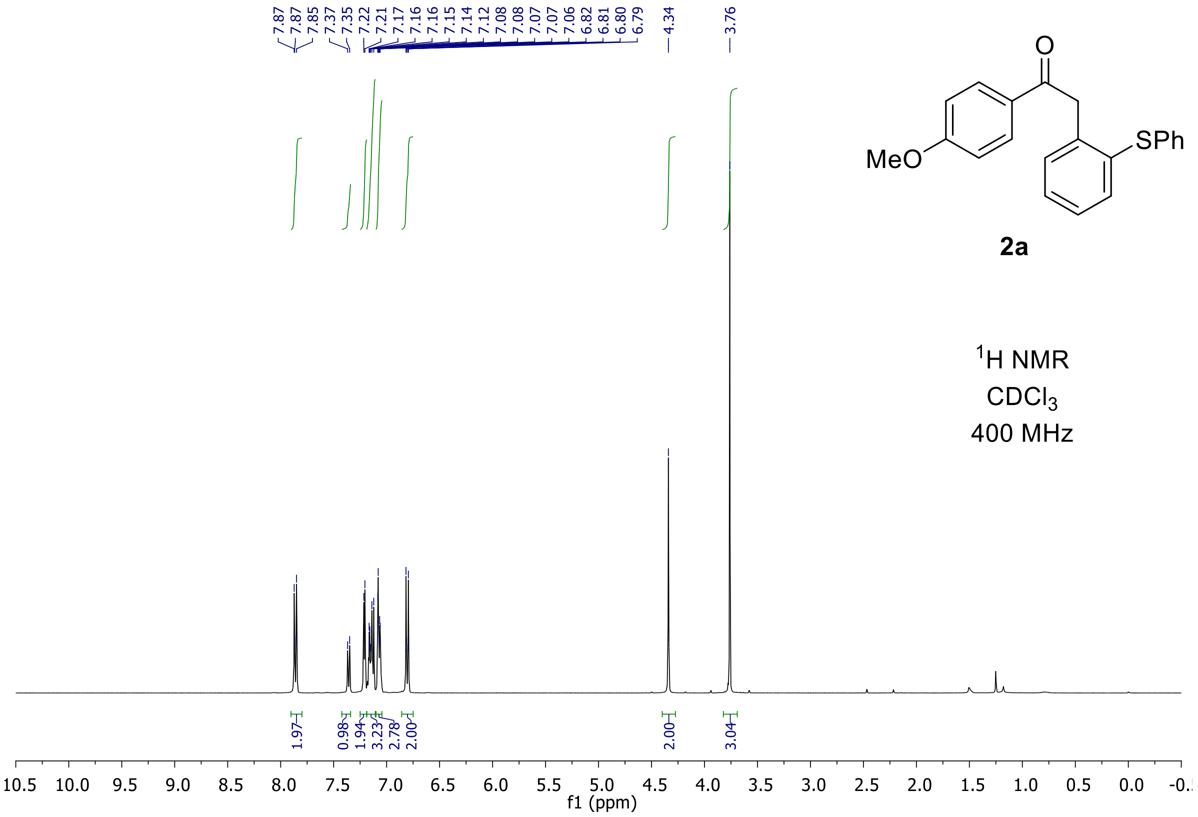

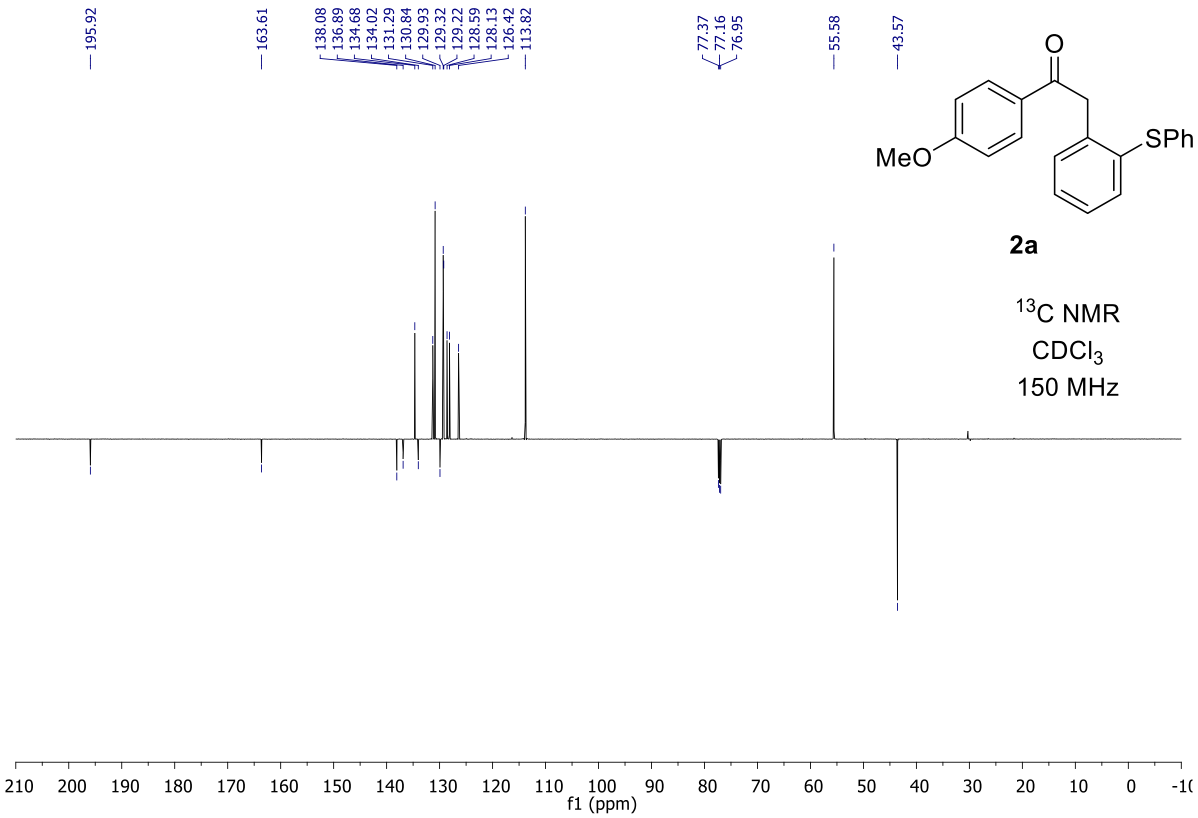

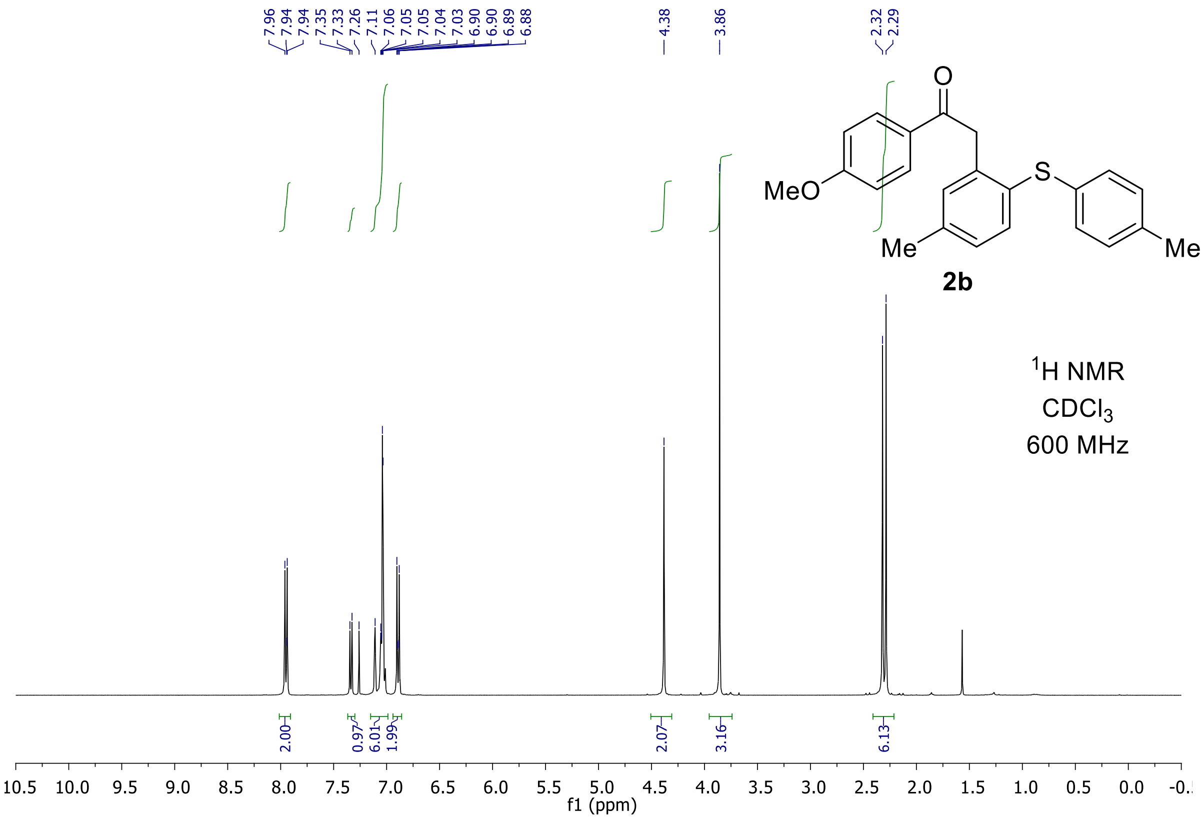
MeO
Me
S
Me
O
2b
^{1}H NMR
$CDCl_3$
600 MHz
f1 (ppm)

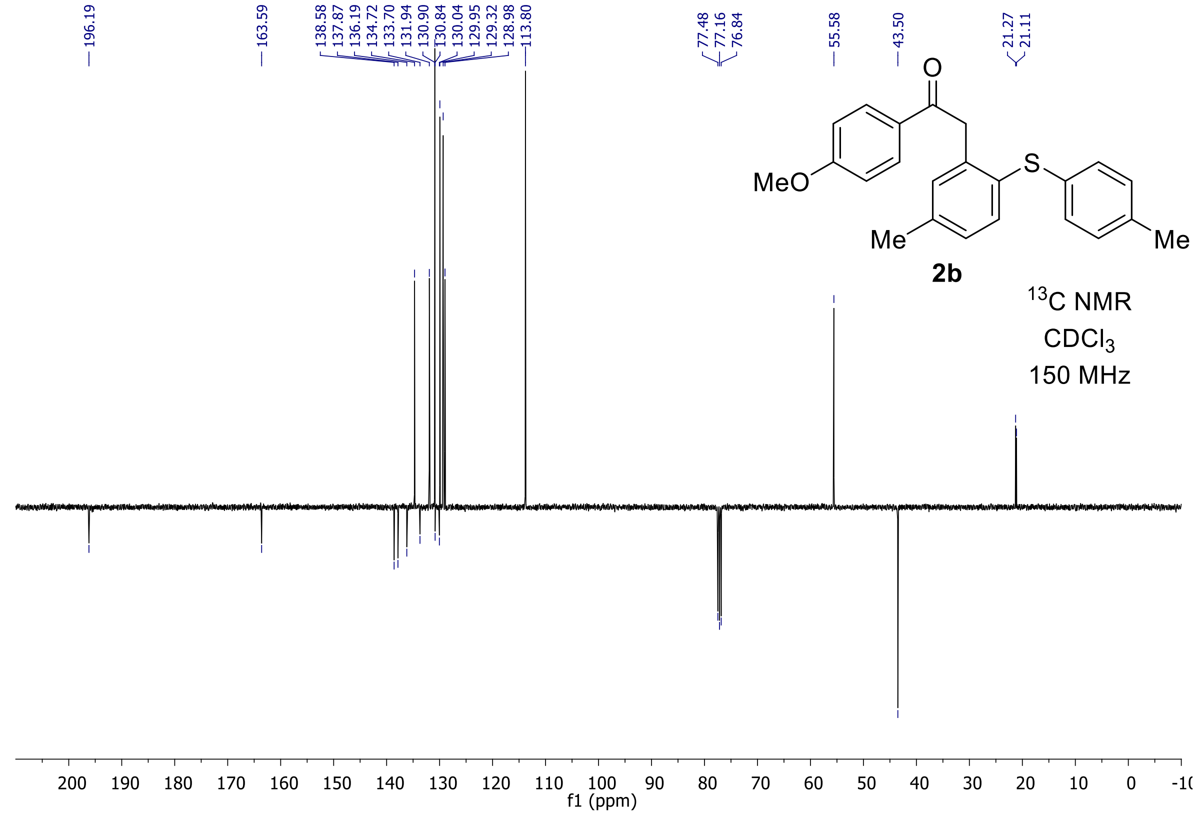
MeO
Me
S
Me
O
2b
^{13}C NMR
$CDCl_3$
150 MHz
f1 (ppm)

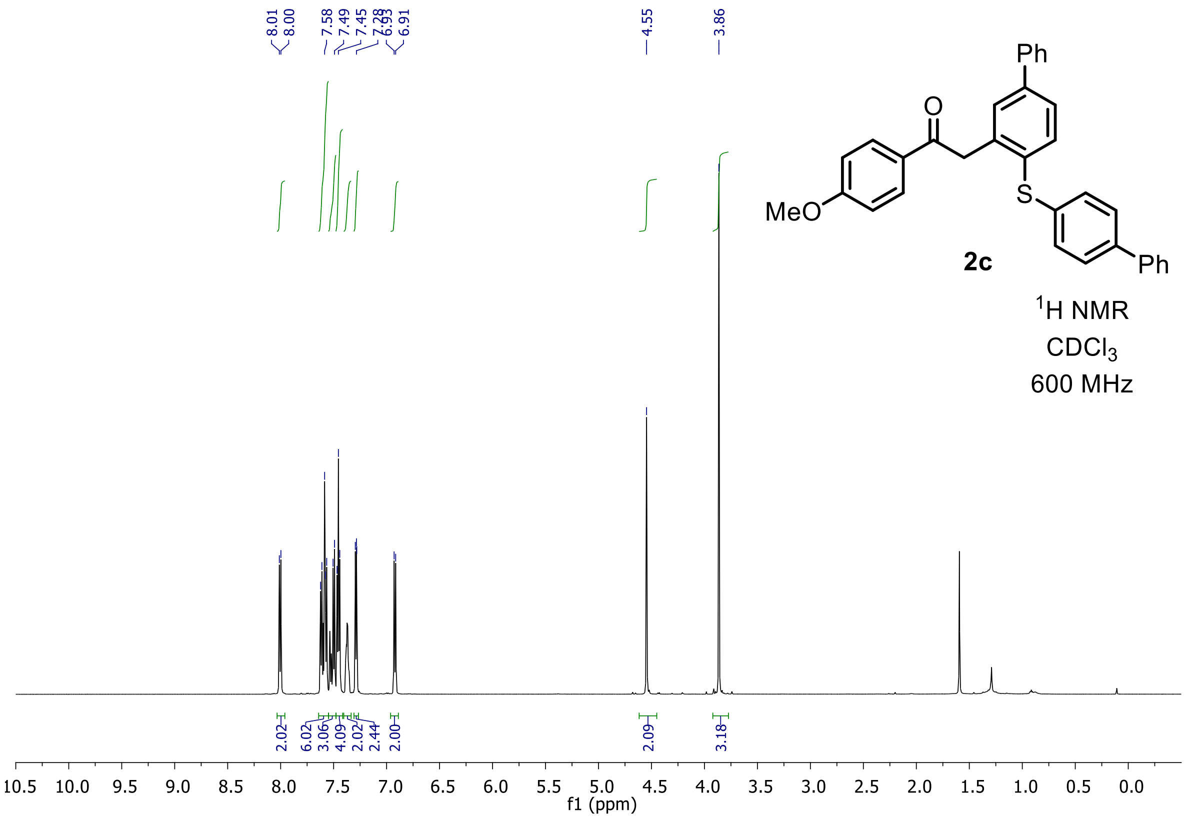
8.01
8.00
7.58
7.49
7.45
7.28
6.93
6.91
4.55
3.86
2.02
6.02
3.06
4.09
2.02
2.44
2.00
2.09
3.18
Ph
O
S
MeO
Ph
2c
^{1}H NMR
CDCl$_3$
600 MHz
f1 (ppm)

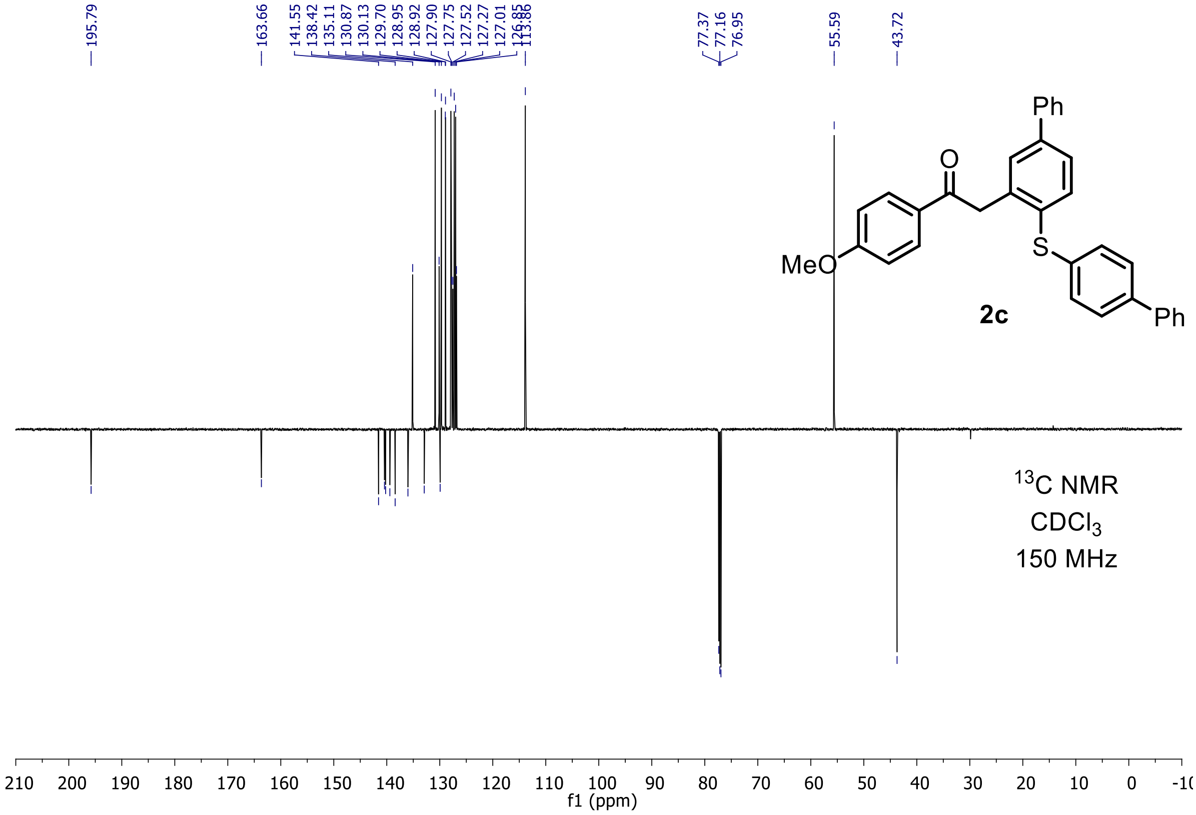
195.79
163.66
141.55
138.42
135.11
130.87
130.13
129.70
128.95
128.92
127.90
127.75
127.52
127.27
127.01
126.85
113.86
77.37
77.16
76.95
55.59
43.72
Ph
O
S
MeO
Ph
2c
^{13}C NMR
CDCl$_3$
150 MHz
f1 (ppm)

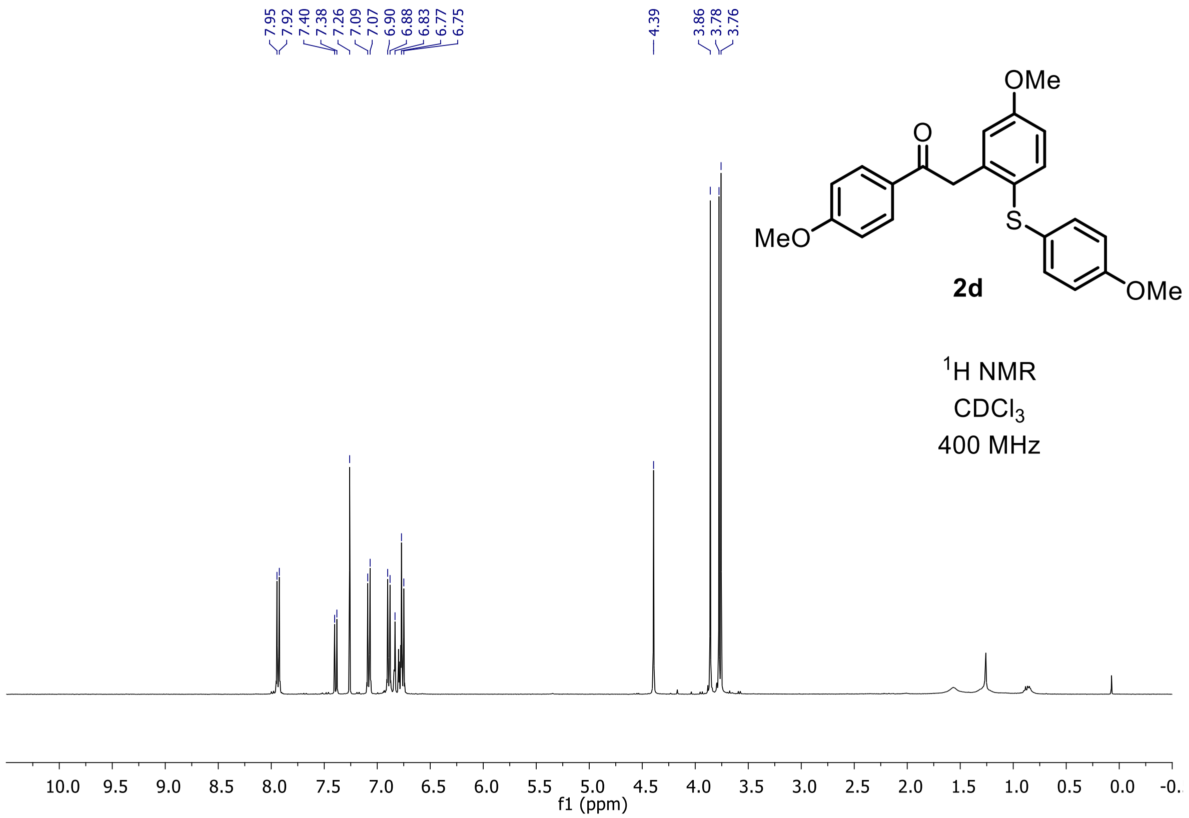
7.95
7.92
7.40
7.38
7.26
7.09
7.07
6.90
6.88
6.83
6.77
6.75
4.39
3.86
3.78
3.76
OMe
O
MeO
S
2d
OMe
1H NMR
CDCl3
400 MHz
10.0 9.5 9.0 8.5 8.0 7.5 7.0 6.5 6.0 5.5 5.0 4.5 4.0 3.5 3.0 2.5 2.0 1.5 1.0 0.5 0.0
f1 (ppm)

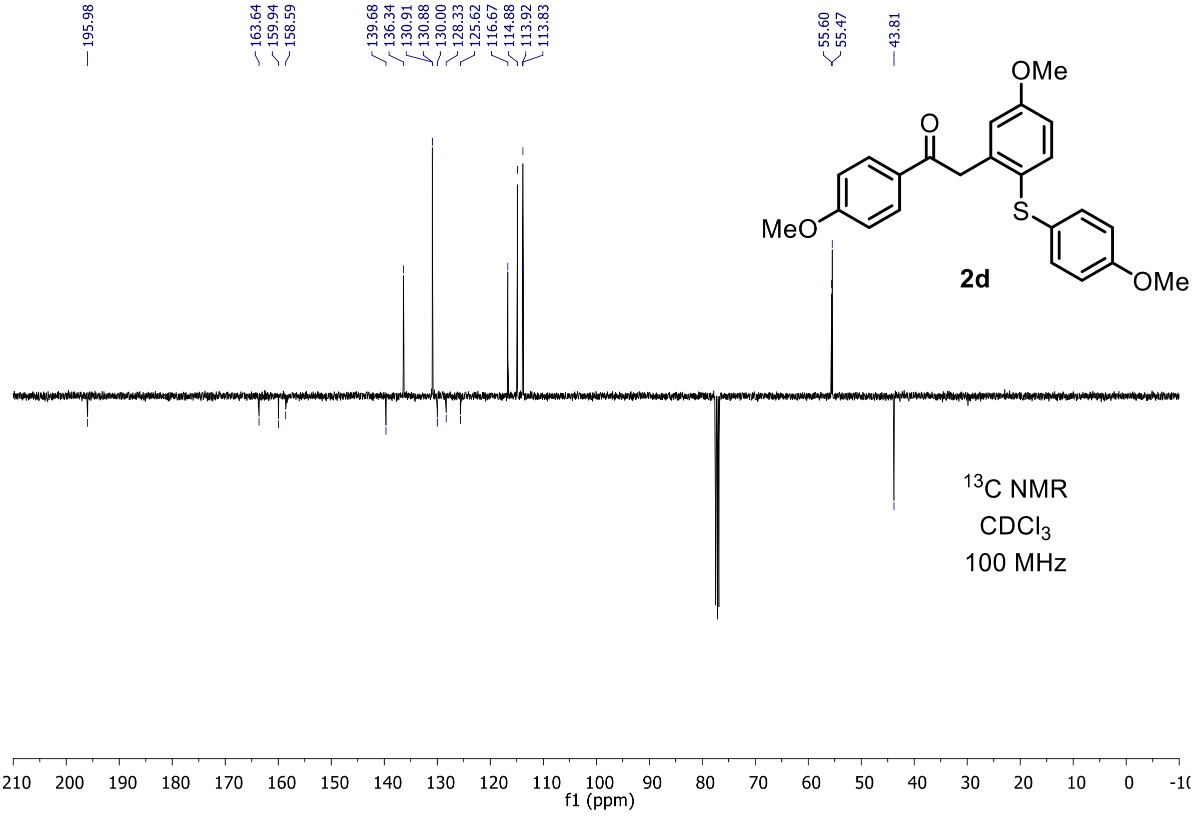
195.98
163.64
159.94
158.59
139.68
136.34
130.91
130.88
130.00
128.33
125.62
116.67
114.88
113.92
113.83
55.60
55.47
43.81
OMe
O
MeO
S
2d
OMe
13C NMR
CDCl3
100 MHz
210 200 190 180 170 160 150 140 130 120 110 100 90 80 70 60 50 40 30 20 10 0
f1 (ppm)

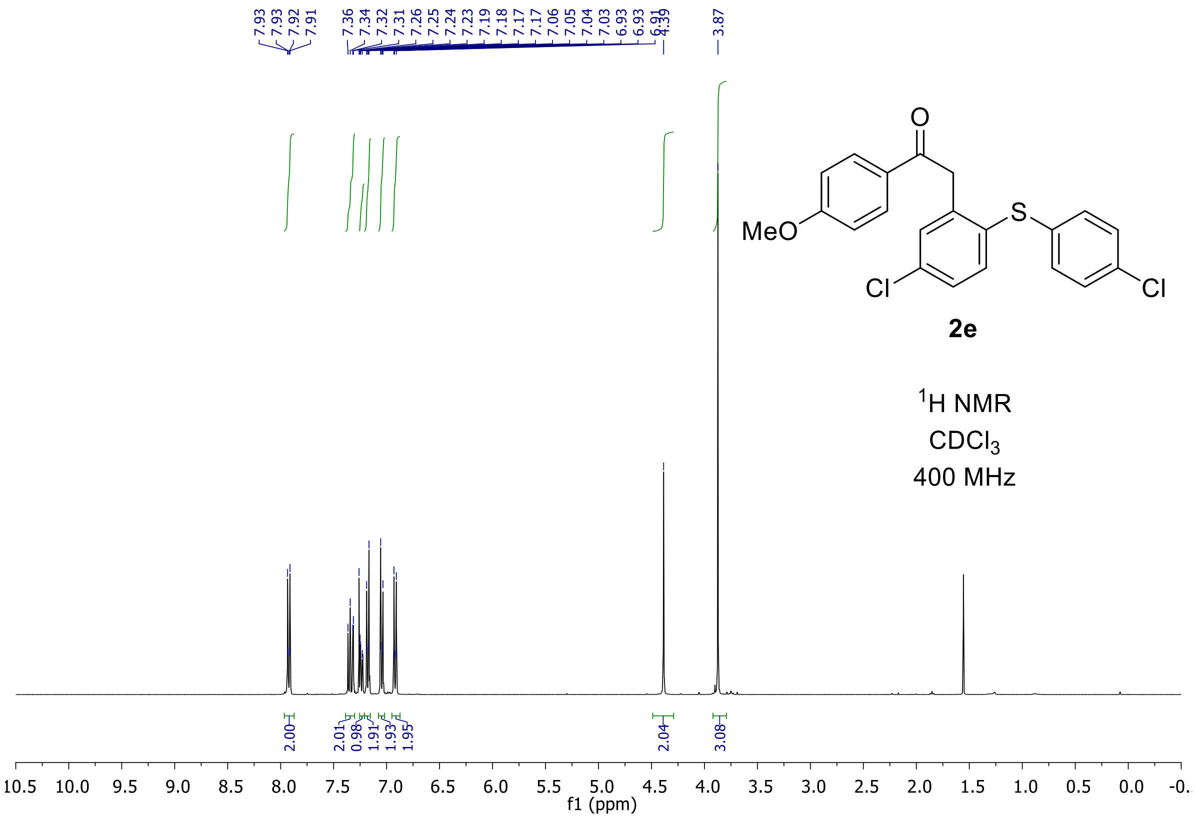
7.93
7.93
7.92
7.91
7.36
7.34
7.32
7.31
7.26
7.25
7.24
7.23
7.19
7.18
7.17
7.06
7.05
7.04
7.03
6.93
6.93
6.91
4.39
3.87
MeO
O
S
Cl
Cl
2e
^{1}H NMR
$CDCl_3$
400 MHz
2.00
2.01
0.98
1.91
1.93
1.95
2.04
3.08
f1 (ppm)

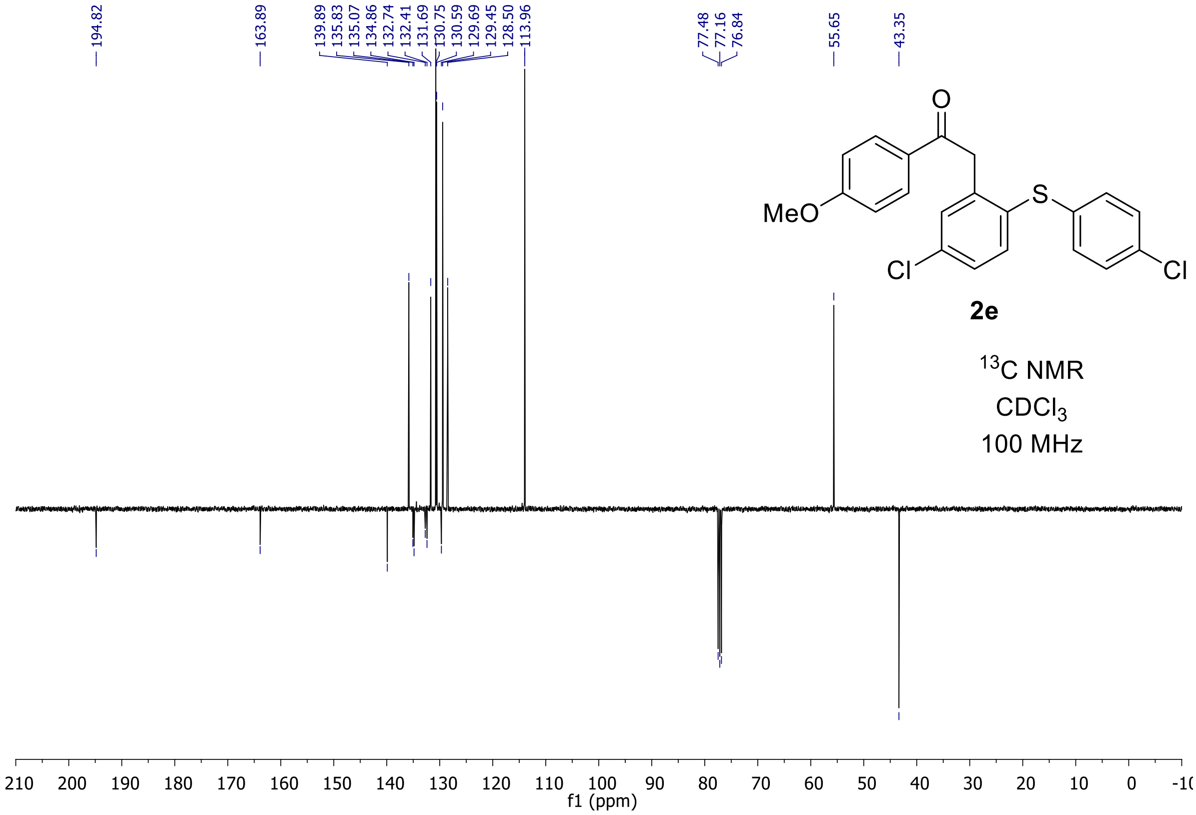
194.82
163.89
139.89
135.83
135.07
134.86
132.74
132.41
131.69
130.75
130.59
129.69
129.45
128.50
113.96
77.48
77.16
76.84
55.65
43.35
MeO
O
S
Cl
Cl
2e
^{13}C NMR
$CDCl_3$
100 MHz
f1 (ppm)

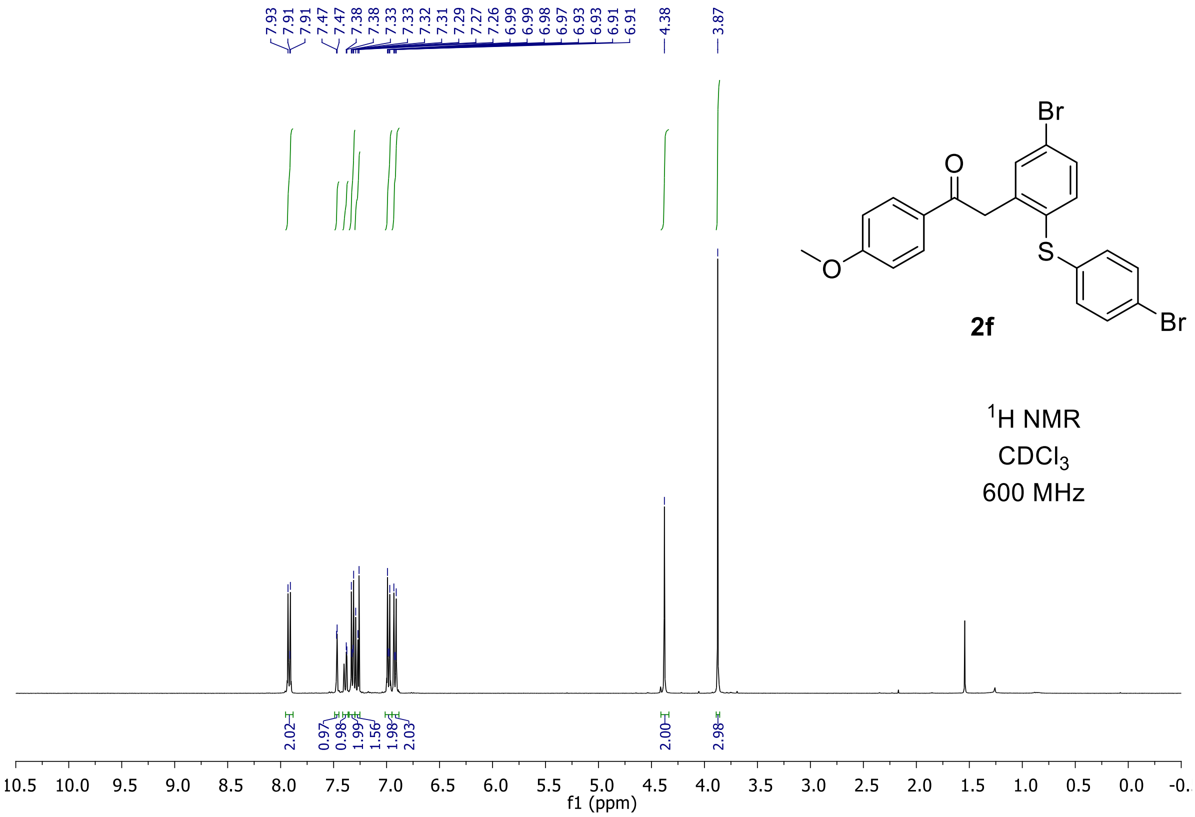
2f
1H NMR
CDCl3
600 MHz
f1 (ppm)

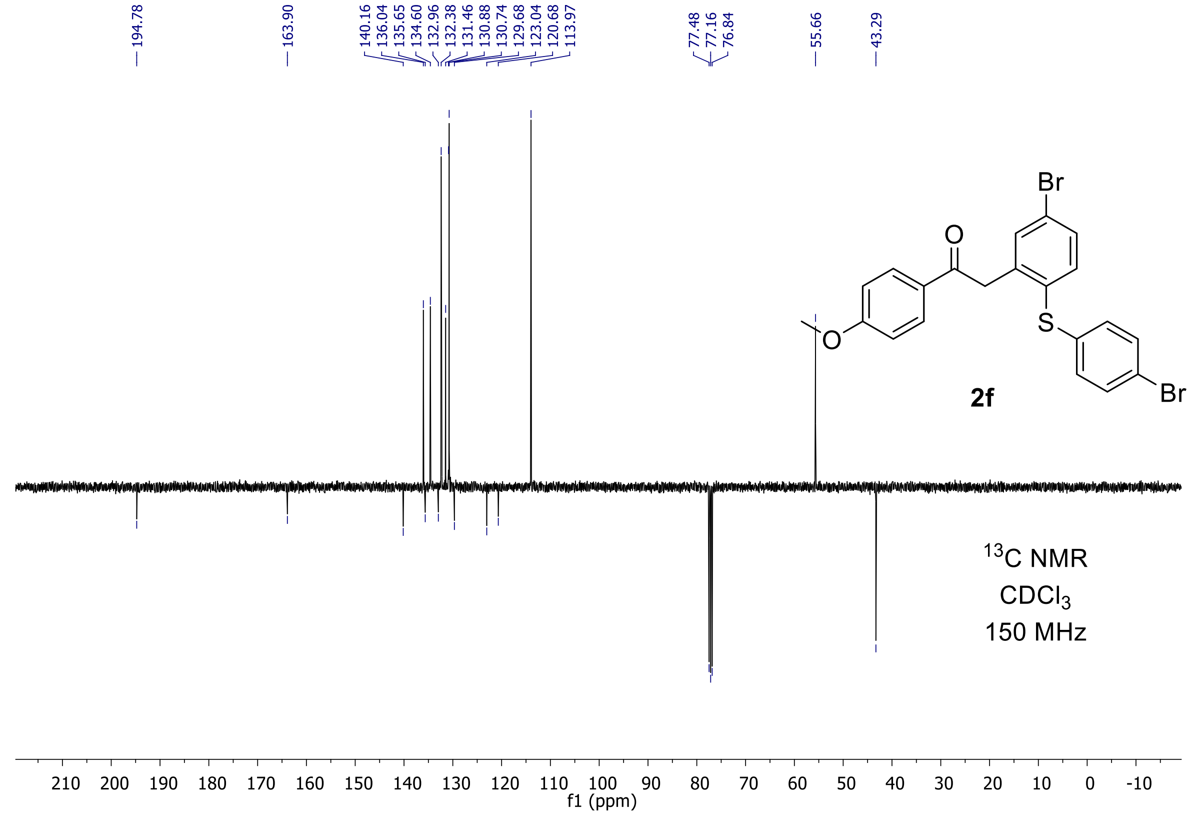
2f
13C NMR
CDCl3
150 MHz
f1 (ppm)

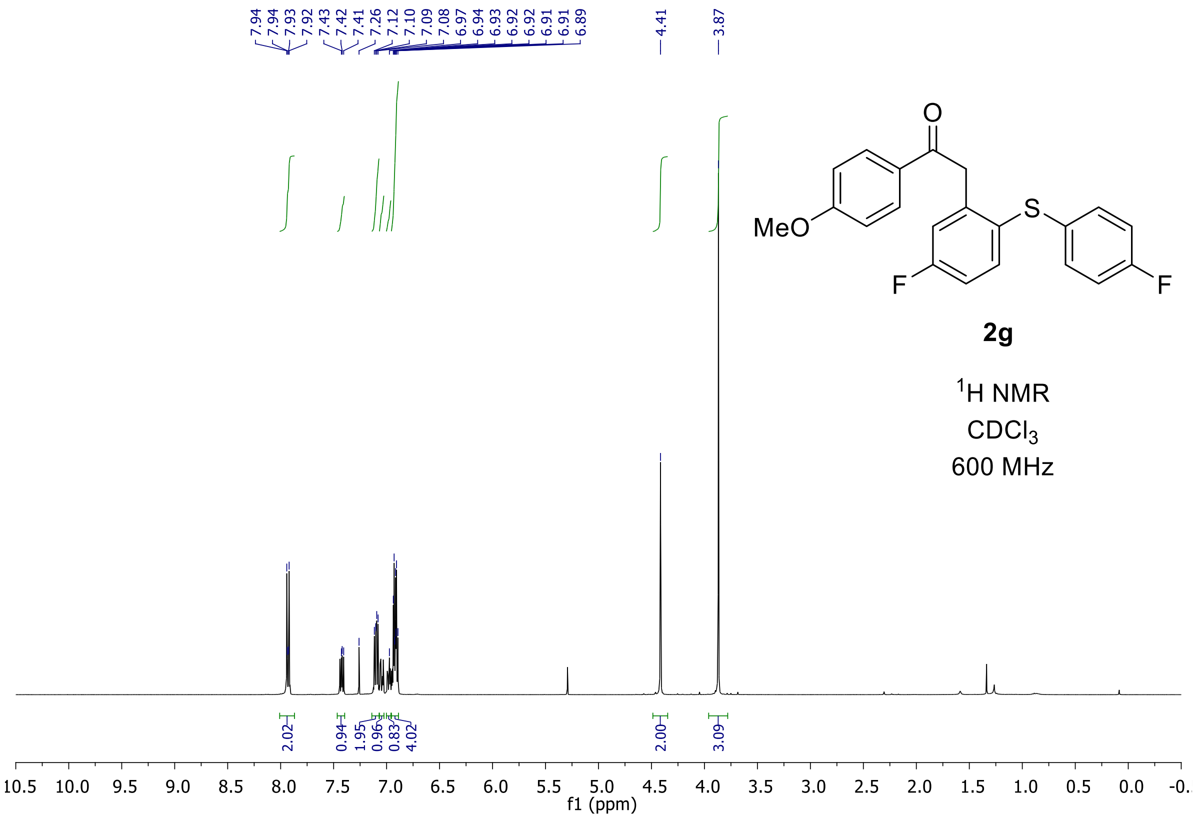
7.94
7.94
7.93
7.92
7.43
7.42
7.41
7.26
7.12
7.10
7.09
7.08
6.97
6.94
6.93
6.92
6.92
6.91
6.89
4.41
3.87
MeO
O
S
F
F
2g
1H NMR
CDCl3
600 MHz
2.02
0.94
1.95
0.96
0.83
4.02
2.00
3.09
f1 (ppm)

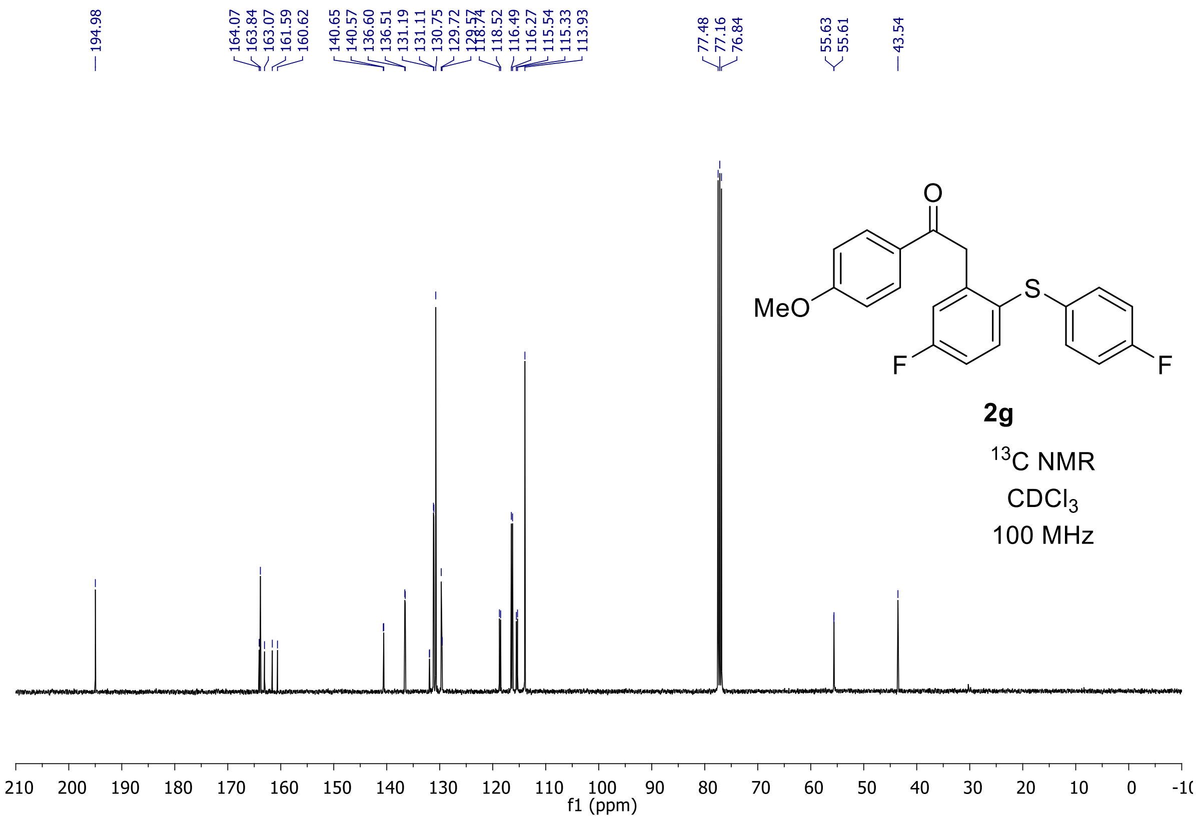
194.98
164.07
163.84
163.07
161.59
160.62
140.65
140.57
136.60
136.51
131.19
131.11
130.75
129.72
129.57
118.74
118.52
116.49
116.27
115.54
115.33
113.93
77.48
77.16
76.84
55.63
55.61
43.54
MeO
O
S
F
F
2g
13C NMR
CDCl3
100 MHz
f1 (ppm)

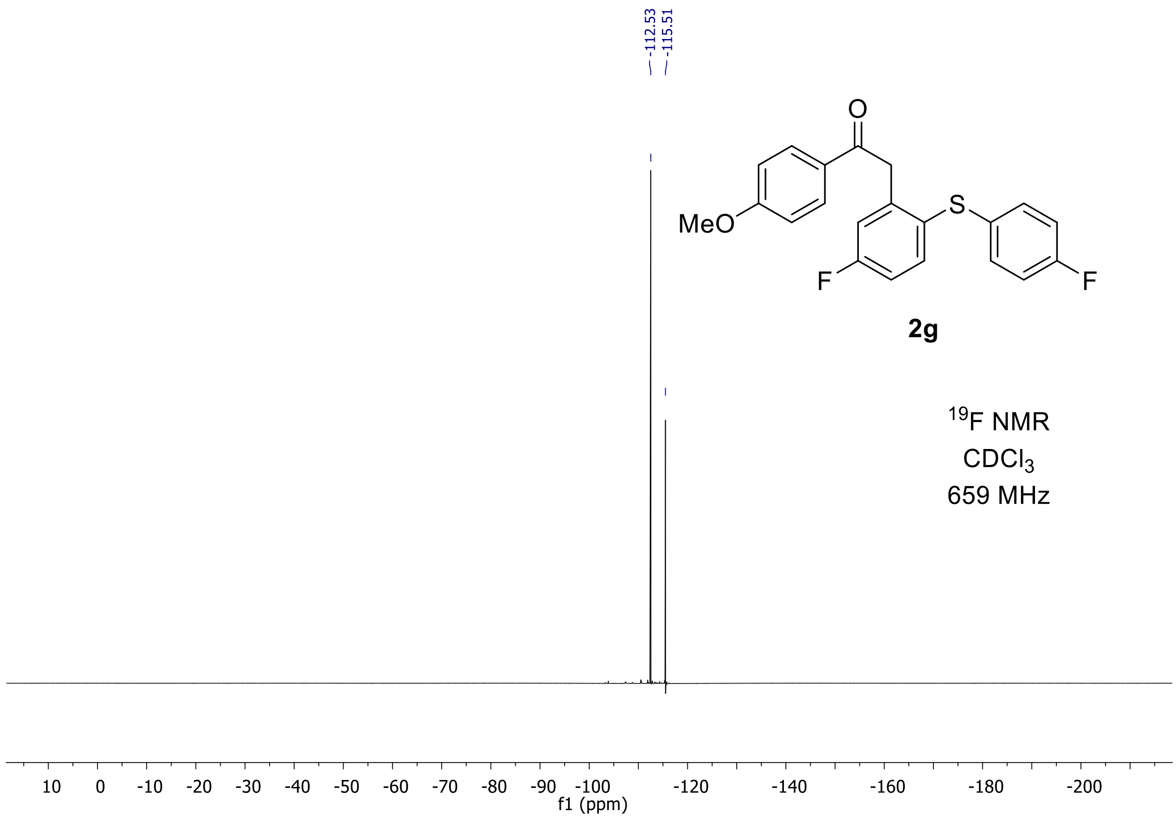

-112.53
-115.51
O
MeO
S
F
F
2g
19F NMR
CDCl3
659 MHz
10 0 -10 -20 -30 -40 -50 -60 -70 -80 -90 -100 -120 -140 -160 -180 -200
f1 (ppm)

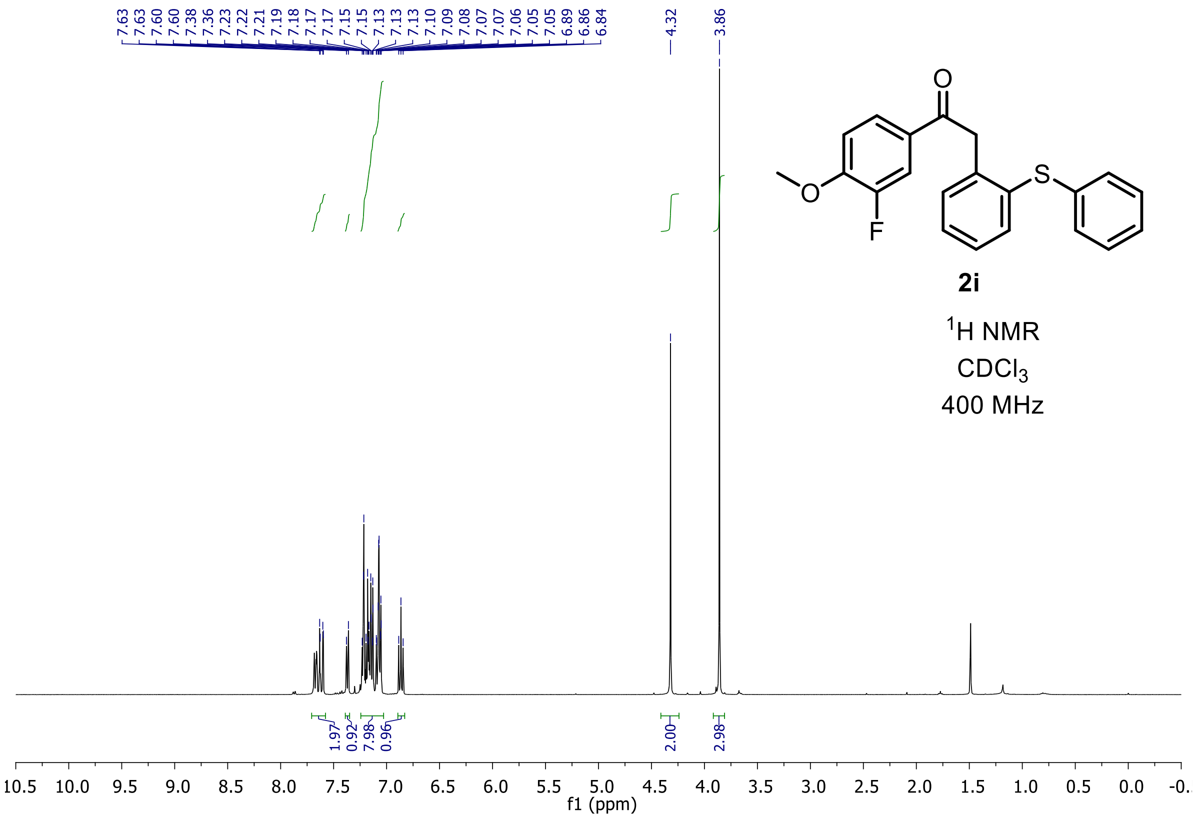
7.63
7.63
7.60
7.60
7.38
7.36
7.23
7.22
7.21
7.19
7.18
7.17
7.17
7.15
7.15
7.13
7.13
7.13
7.10
7.09
7.08
7.07
7.07
7.06
7.05
7.05
6.89
6.86
6.84
4.32
3.86
O
S
O
F
2i
1H NMR
CDCl3
400 MHz
1.97
0.92
7.98
0.96
2.00
2.98
f1 (ppm)

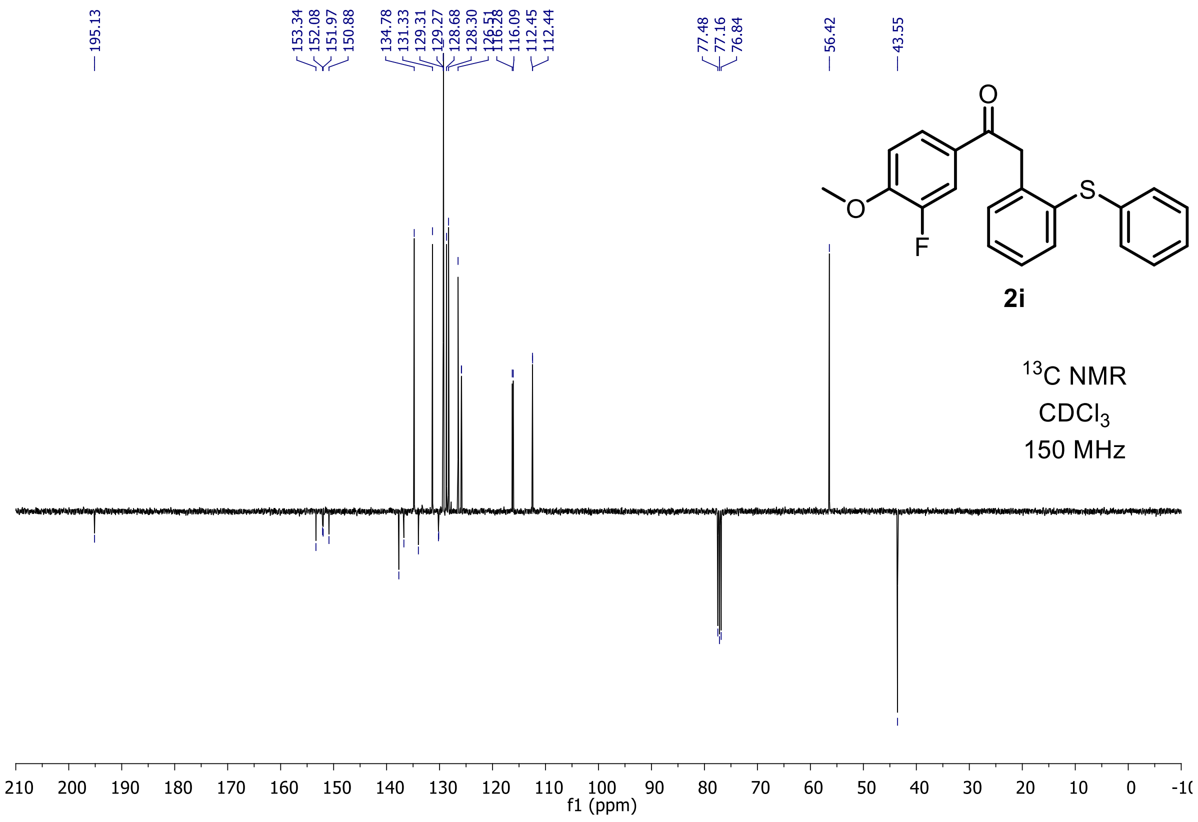
195.13
153.34
152.08
151.97
150.88
134.78
131.33
129.31
129.27
128.68
128.30
126.51
126.28
116.09
112.45
112.44
77.48
77.16
76.84
56.42
43.55
O
S
O
F
2i
13C NMR
CDCl3
150 MHz
f1 (ppm)

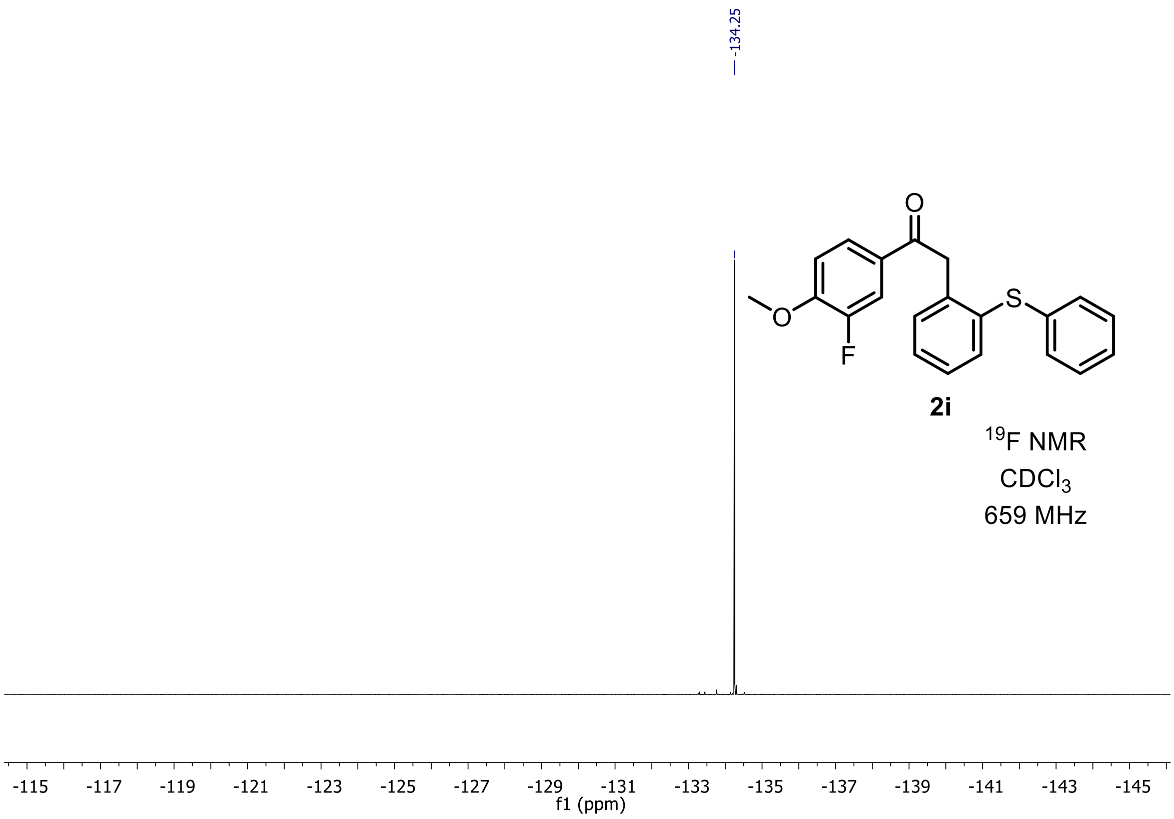
-134.25
O
O
F
S
2i
^{19}F NMR
CDCl$_3$
659 MHz
-115 -117 -119 -121 -123 -125 -127 -129 -131 -133 -135 -137 -139 -141 -143 -145
f1 (ppm)

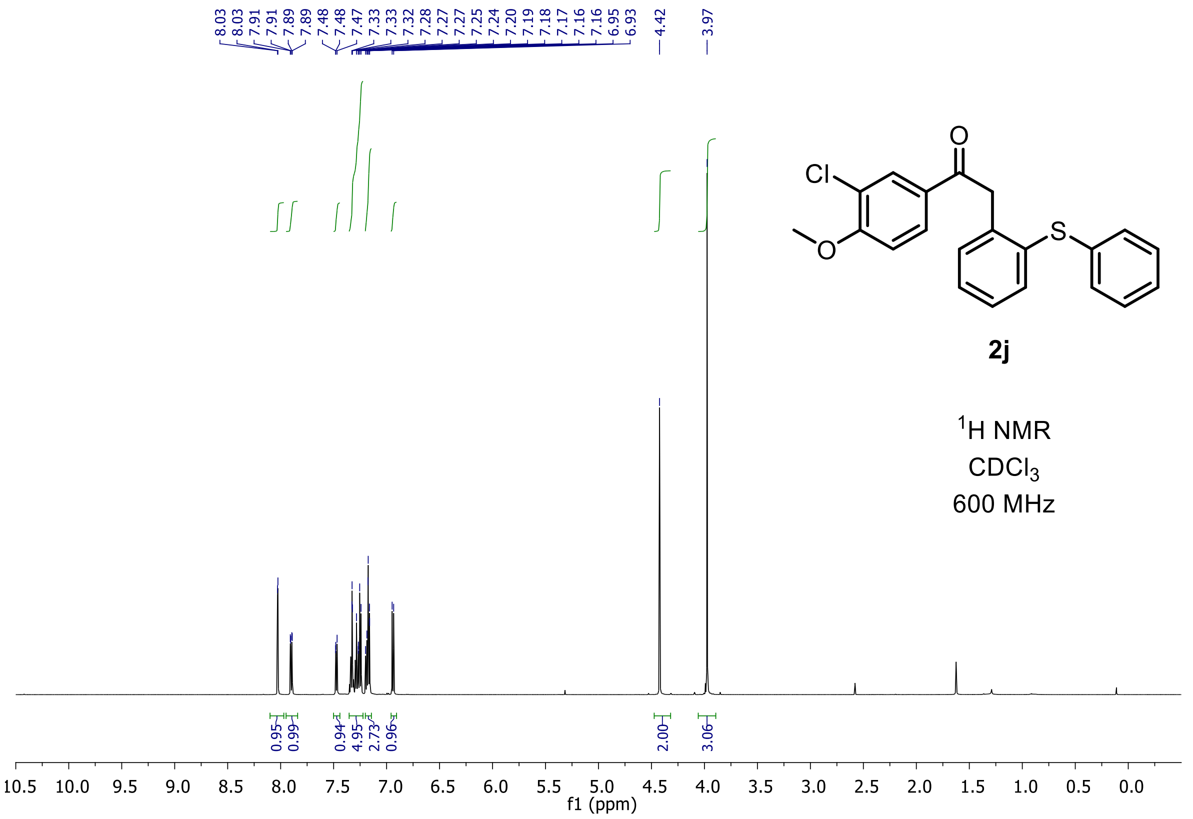
8.03
8.03
7.91
7.89
7.89
7.48
7.48
7.47
7.33
7.33
7.32
7.28
7.27
7.27
7.25
7.24
7.20
7.19
7.18
7.17
7.16
7.16
6.95
6.93
4.42
3.97
0.95
0.99
0.94
4.95
2.73
0.96
2.00
3.06
Cl
O
S
O
2j
^{1}H NMR
$CDCl_3$
600 MHz
10.5 10.0 9.5 9.0 8.5 8.0 7.5 7.0 6.5 6.0 5.5 5.0 4.5 4.0 3.5 3.0 2.5 2.0 1.5 1.0 0.5 0.0
f1 (ppm)

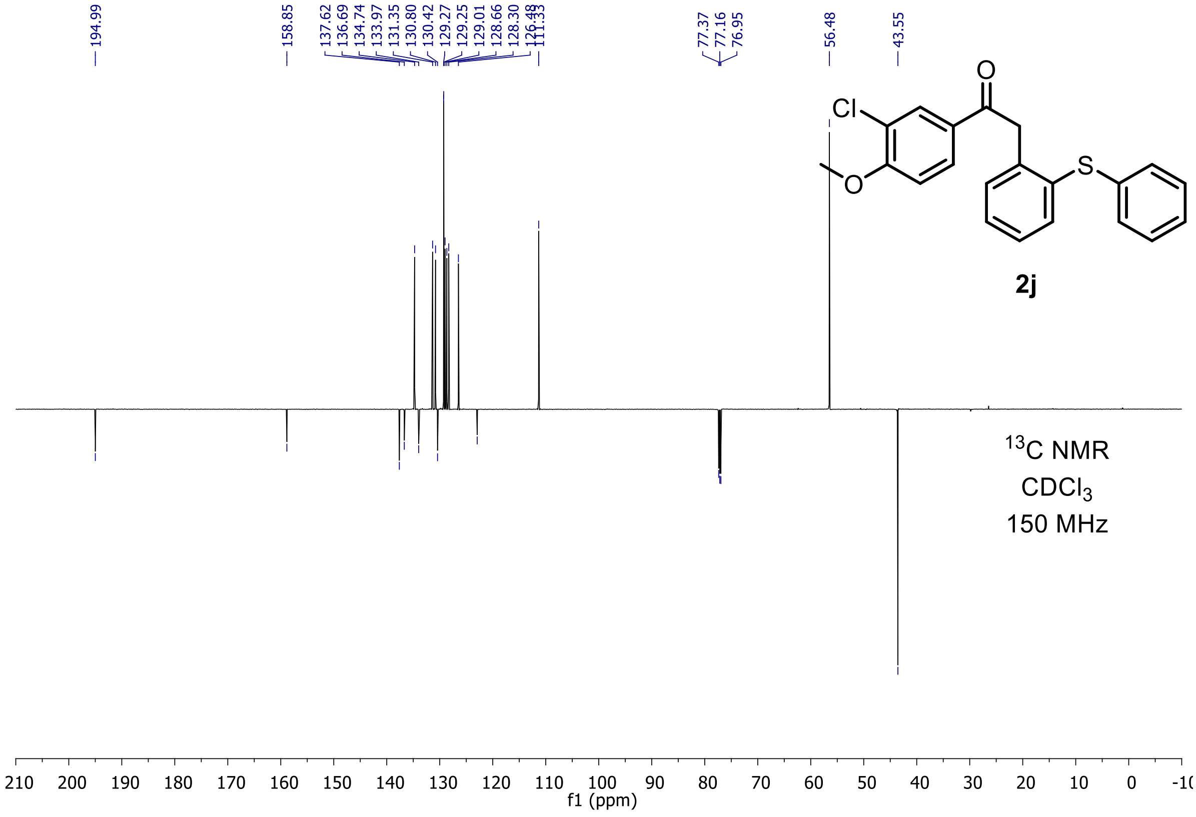
194.99
158.85
137.62
136.69
134.74
133.97
131.35
130.80
130.42
129.27
129.25
129.01
128.66
128.30
126.48
111.33
77.37
77.16
76.95
56.48
43.55
Cl
O
S
O
2j
^{13}C NMR
$CDCl_3$
150 MHz
210 200 190 180 170 160 150 140 130 120 110 100 90 80 70 60 50 40 30 20 10 0 -10
f1 (ppm)

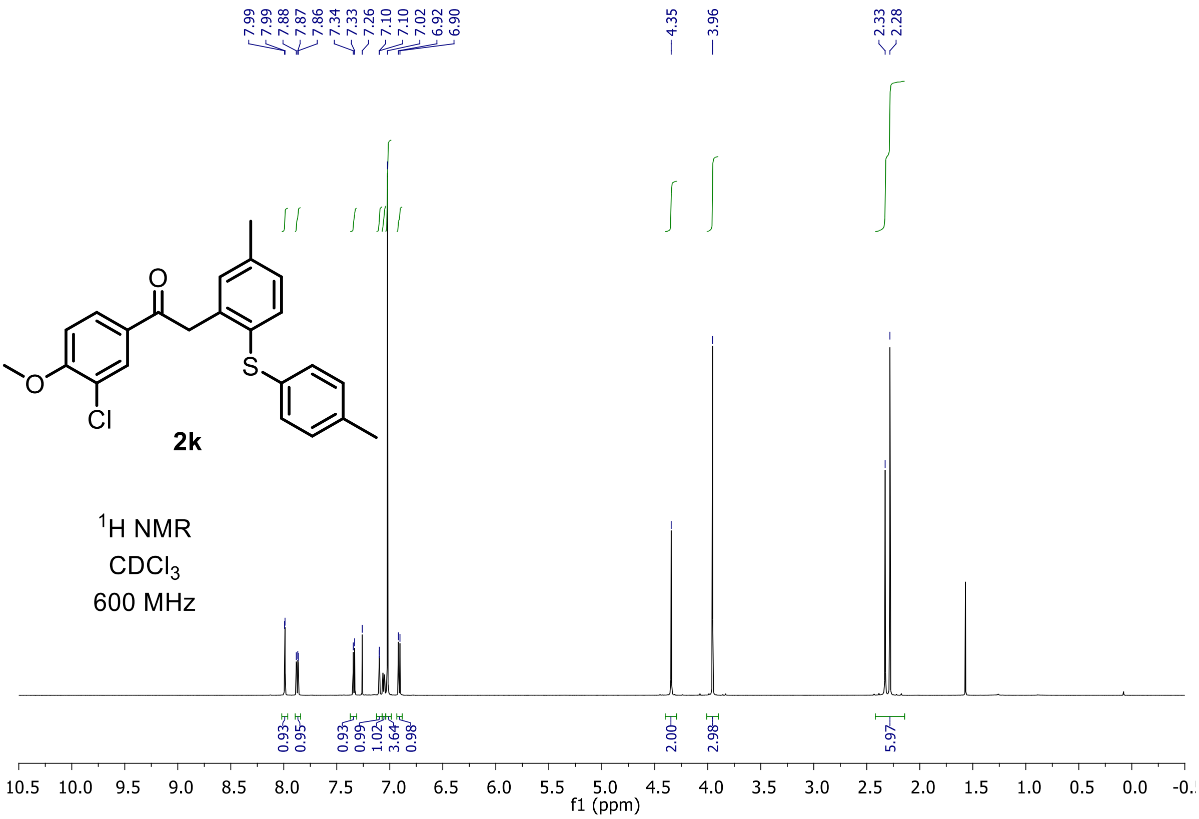
7.99
7.99
7.88
7.87
7.86
7.34
7.33
7.26
7.10
7.10
7.02
6.92
6.90
4.35
3.96
2.33
2.28
O
S
O
Cl
2k
^{1}H NMR
$CDCl_3$
600 MHz
0.93
0.95
0.93
0.99
1.02
3.64
0.98
2.00
2.98
5.97
10.5 10.0 9.5 9.0 8.5 8.0 7.5 7.0 6.5 6.0 5.5 5.0 4.5 4.0 3.5 3.0 2.5 2.0 1.5 1.0 0.5 0.0
f1 (ppm)

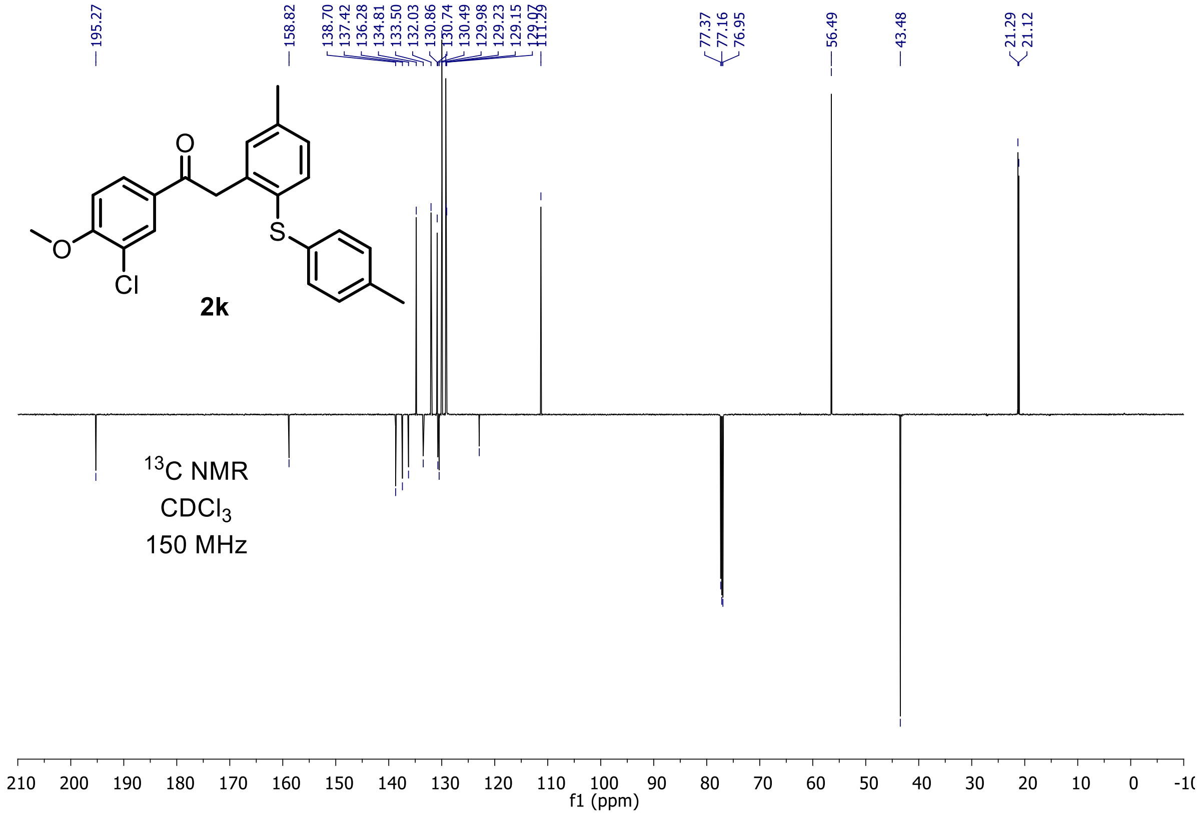
195.27
158.82
138.70
137.42
136.28
134.81
133.50
132.03
130.86
130.74
130.49
129.98
129.23
129.15
129.07
111.29
77.37
77.16
76.95
56.49
43.48
21.29
21.12
O
S
O
Cl
2k
^{13}C NMR
$CDCl_3$
150 MHz
210 200 190 180 170 160 150 140 130 120 110 100 90 80 70 60 50 40 30 20 10 0
f1 (ppm)

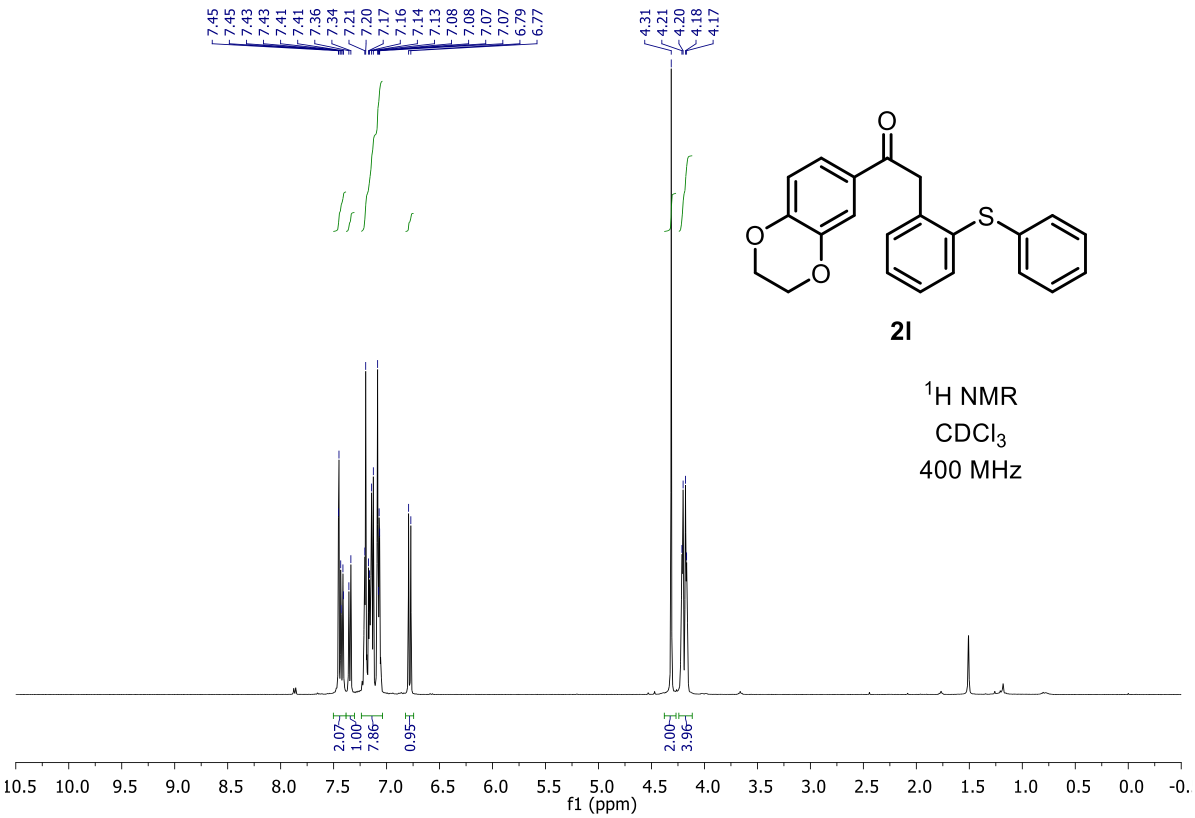
7.45
7.45
7.43
7.43
7.41
7.41
7.36
7.34
7.21
7.20
7.17
7.16
7.14
7.13
7.08
7.08
7.07
7.07
6.79
6.77
4.31
4.21
4.20
4.18
4.17
O
S
O
O
2l
1H NMR
CDCl3
400 MHz
2.07
1.00
7.86
0.95
2.00
3.96
f1 (ppm)

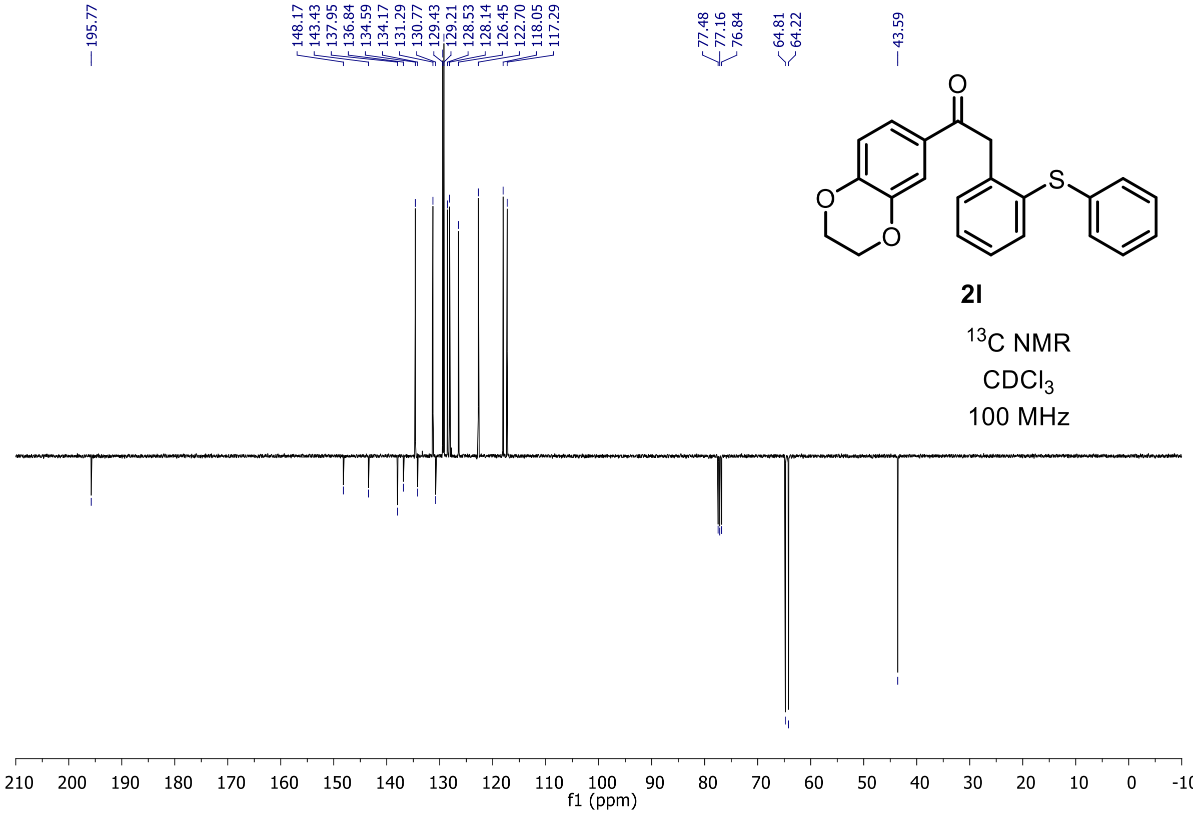
195.77
148.17
143.43
137.95
136.84
134.59
134.17
131.29
130.77
129.43
129.21
128.53
128.14
126.45
122.70
118.05
117.29
77.48
77.16
76.84
64.81
64.22
43.59
O
S
O
O
2l
13C NMR
CDCl3
100 MHz
f1 (ppm)

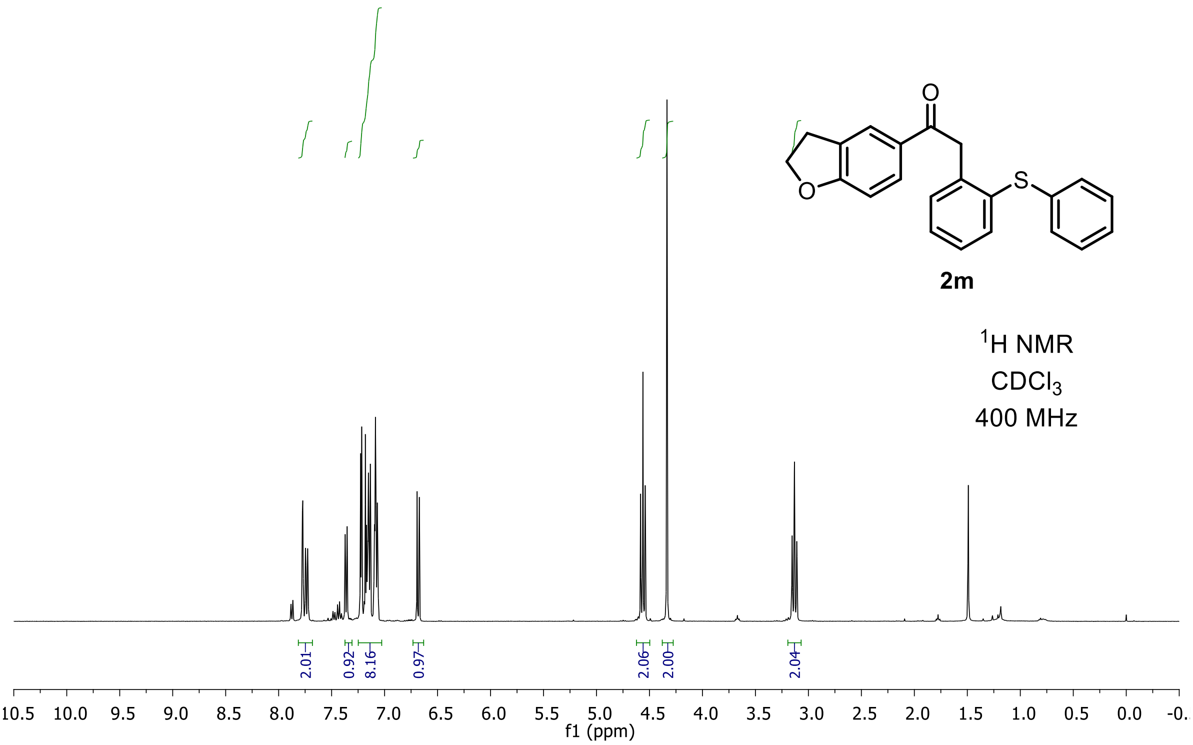
2m
1H NMR
CDCl3
400 MHz
2.01
0.92
8.16
0.97
2.06
2.00
2.04
f1 (ppm)

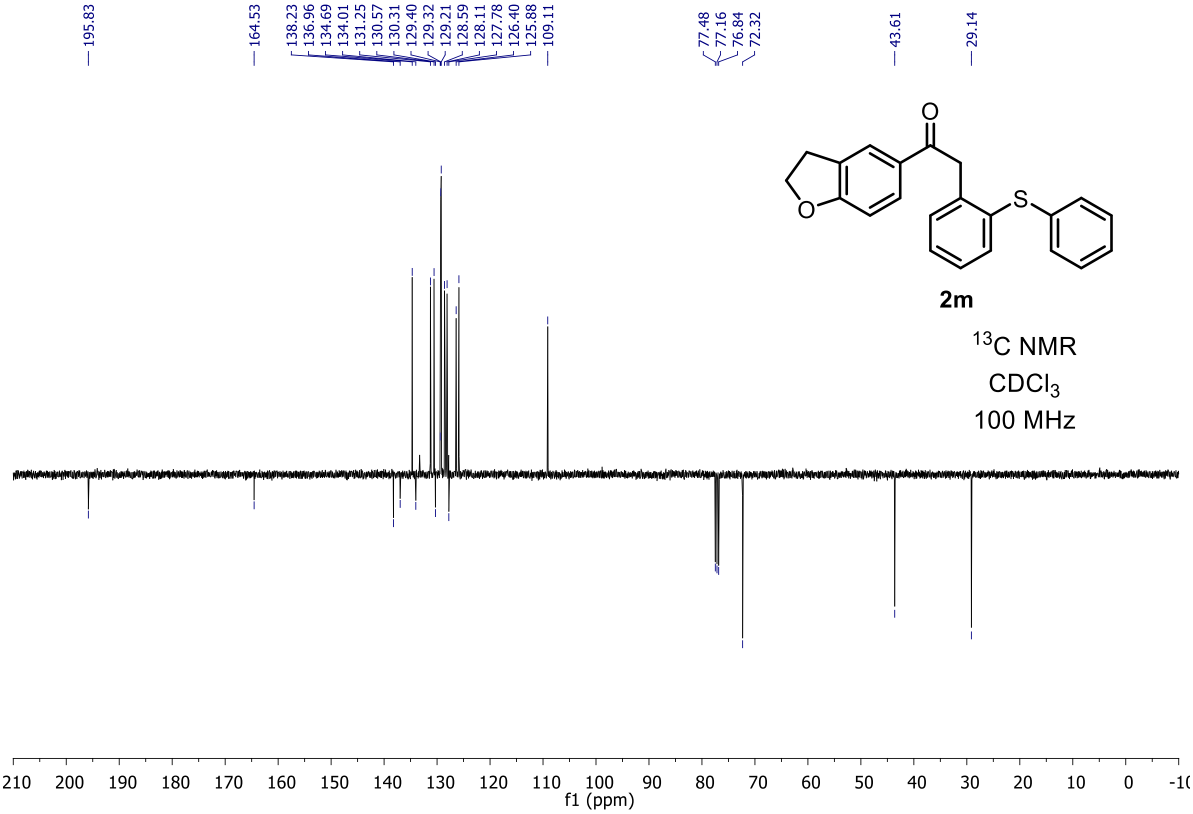
195.83
164.53
138.23
136.96
134.69
134.01
131.25
130.57
130.31
129.40
129.32
129.21
128.59
128.11
127.78
126.40
125.88
109.11
77.48
77.16
76.84
72.32
43.61
29.14
2m
13C NMR
CDCl3
100 MHz
f1 (ppm)

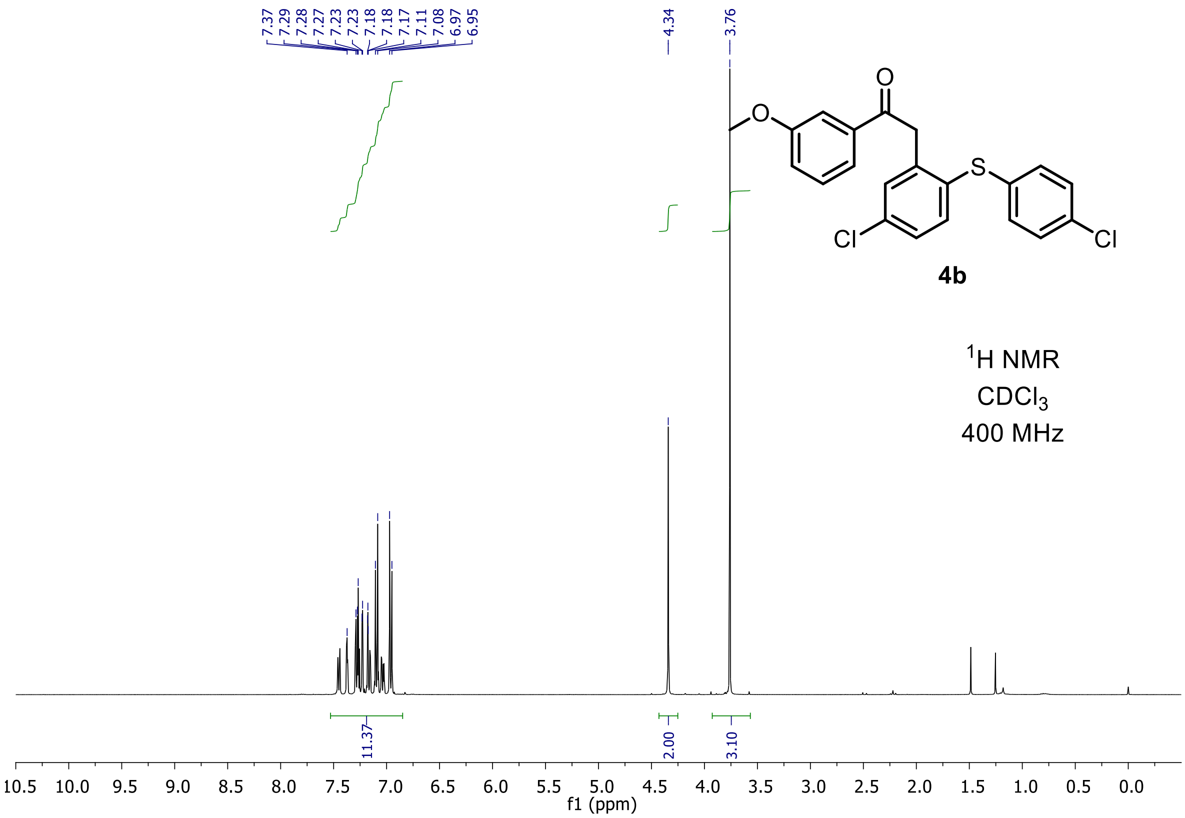

4b
1H NMR
CDCl3
400 MHz
f1 (ppm)

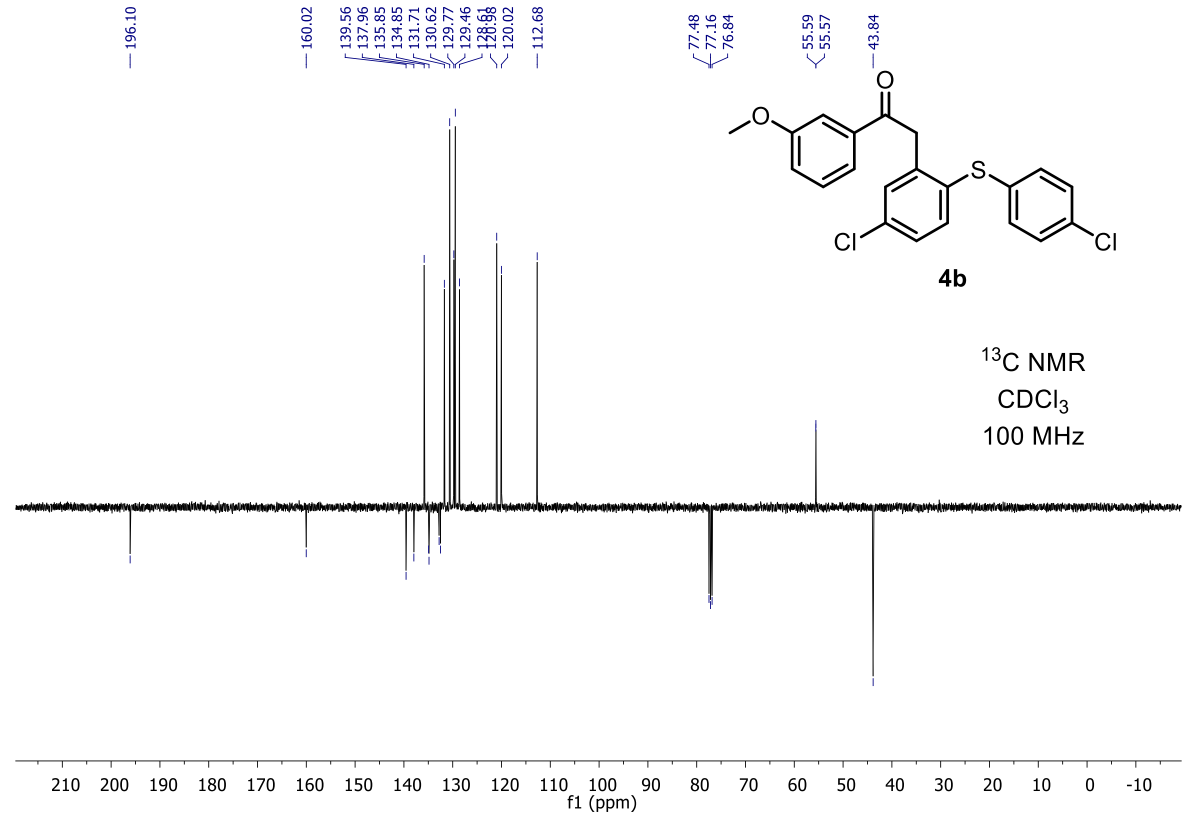

4b
13C NMR
CDCl3
100 MHz
f1 (ppm)

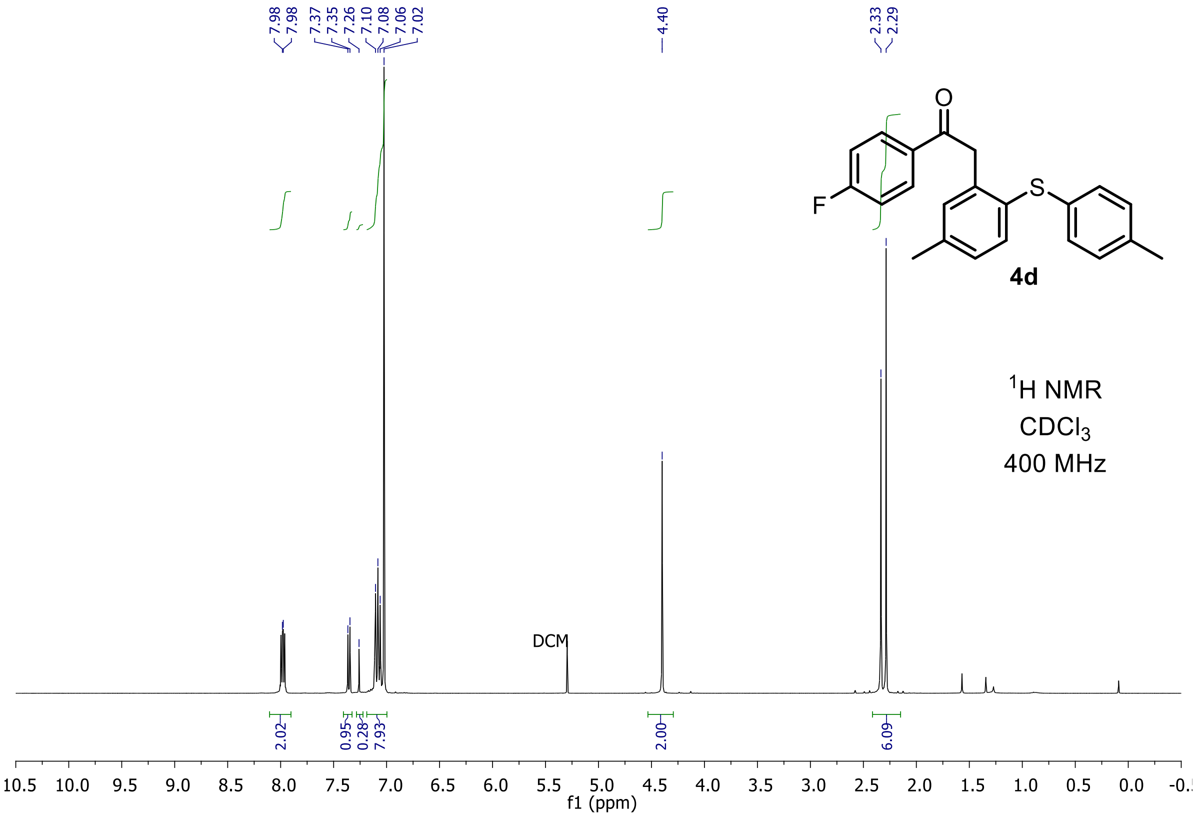
7.98
7.98
7.37
7.35
7.26
7.10
7.08
7.06
7.02
4.40
2.33
2.29
O
S
F
4d
^{1}H NMR
$CDCl_3$
400 MHz
DCM
2.02
0.95
0.28
7.93
2.00
6.09
f1 (ppm)

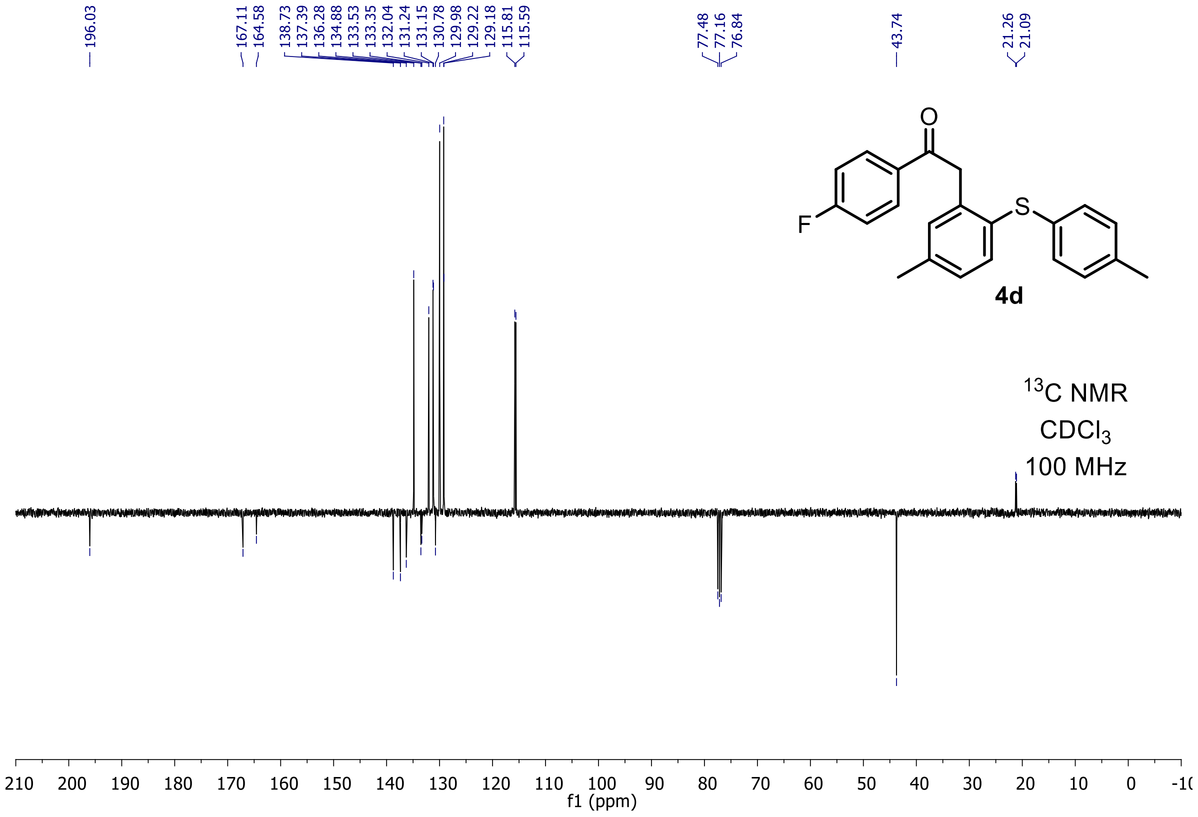
196.03
167.11
164.58
138.73
137.39
136.28
134.88
133.53
133.35
132.04
131.24
131.15
130.78
129.98
129.22
129.18
115.81
115.59
77.48
77.16
76.84
43.74
21.26
21.09
O
S
F
4d
^{13}C NMR
$CDCl_3$
100 MHz
f1 (ppm)

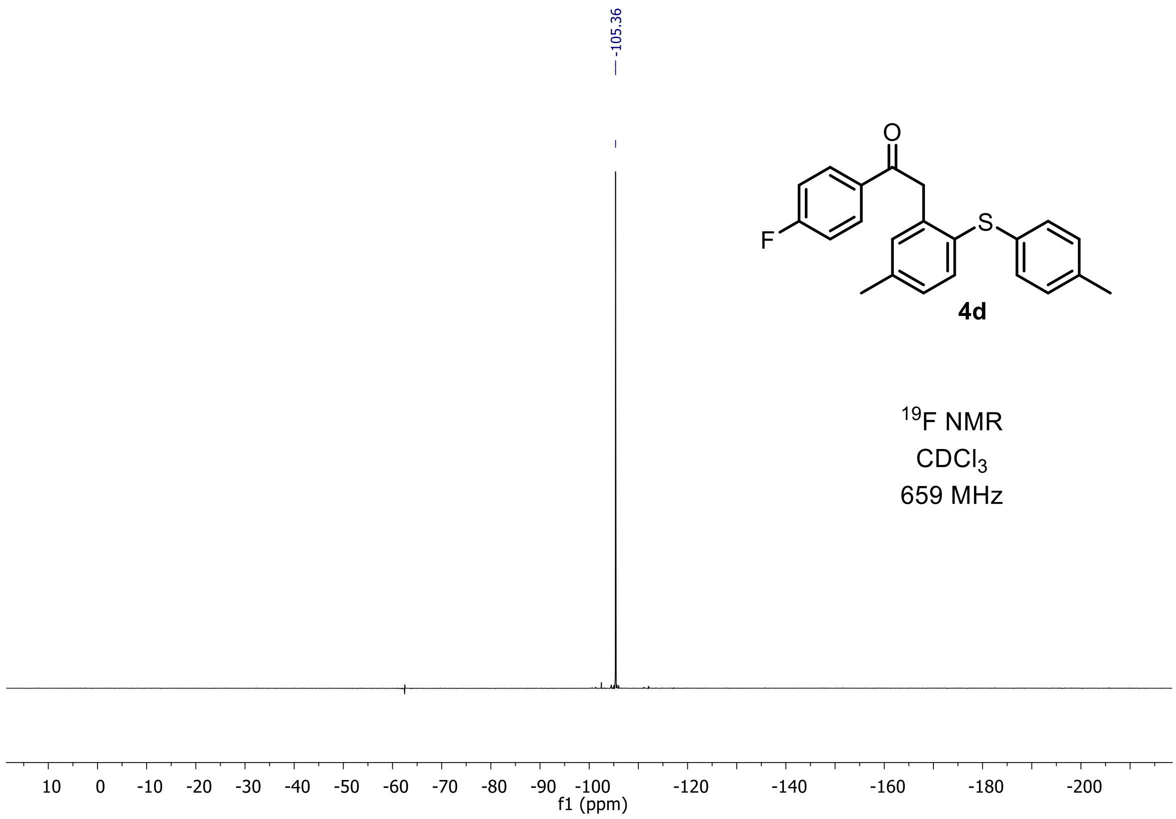
-105.36
O
F
S
4d
^{19}F NMR
$CDCl_3$
659 MHz
10 0 -10 -20 -30 -40 -50 -60 -70 -80 -90 -100 -120 -140 -160 -180 -200
f1 (ppm)

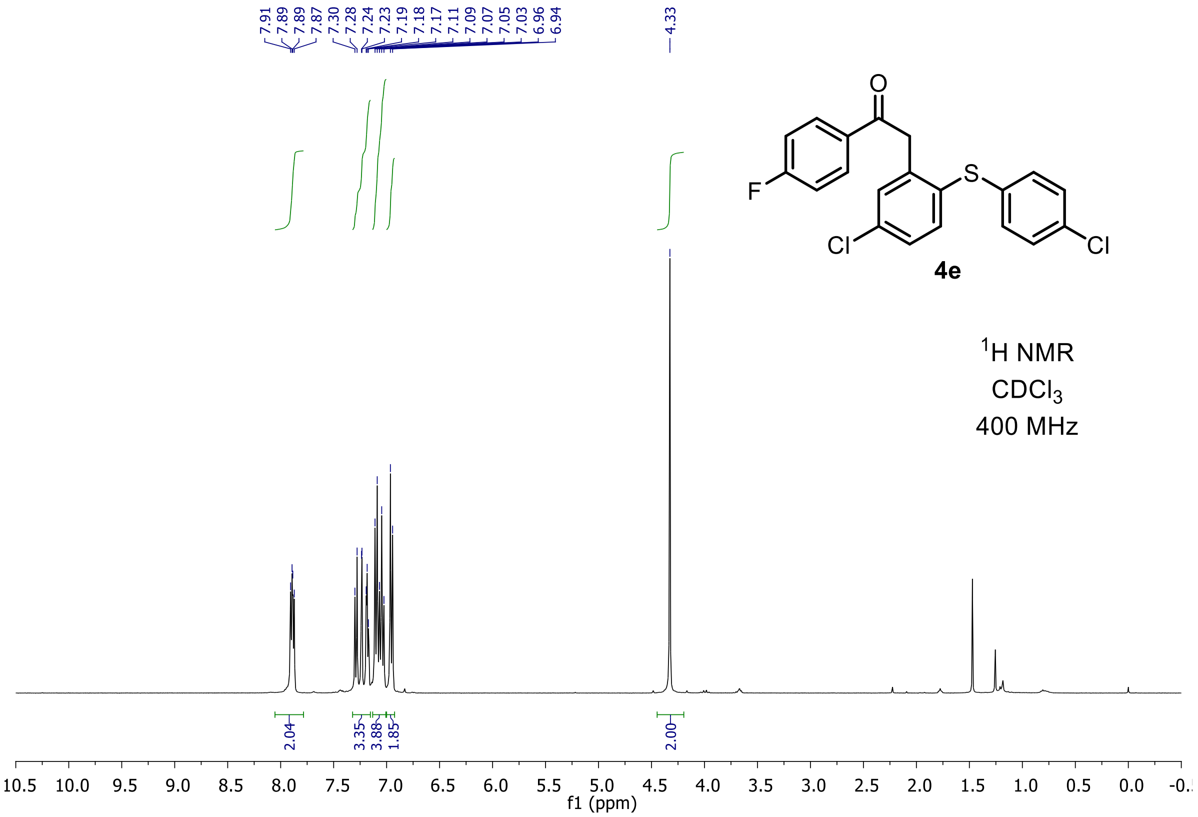
7.91
7.89
7.89
7.87
7.30
7.28
7.24
7.23
7.19
7.18
7.17
7.11
7.09
7.07
7.05
7.03
6.96
6.94
4.33
O
F
S
Cl
Cl
4e
^{1}H NMR
$CDCl_3$
400 MHz
2.04
3.35
3.88
1.85
2.00
f1 (ppm)

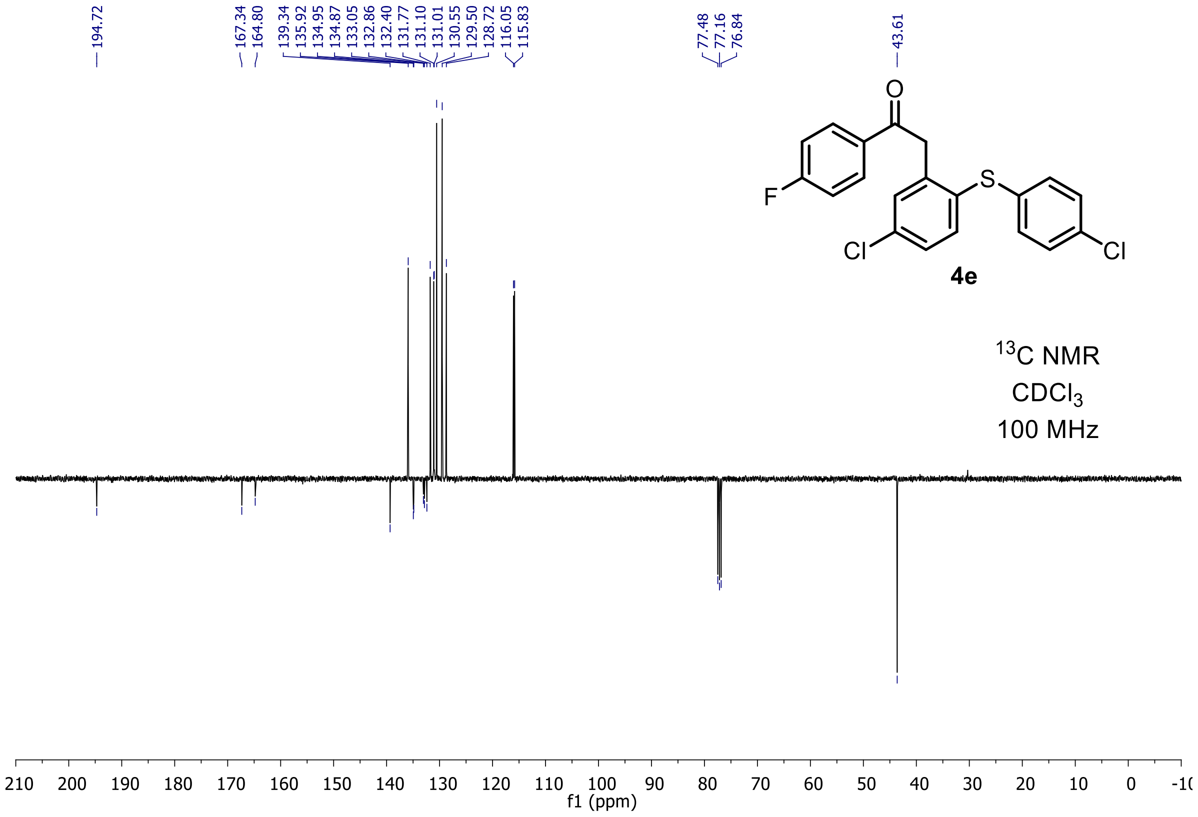
194.72
167.34
164.80
139.34
135.92
134.95
134.87
133.05
132.86
132.40
131.77
131.10
131.01
130.55
129.50
128.72
116.05
115.83
77.48
77.16
76.84
43.61
O
F
S
Cl
Cl
4e
^{13}C NMR
$CDCl_3$
100 MHz
f1 (ppm)

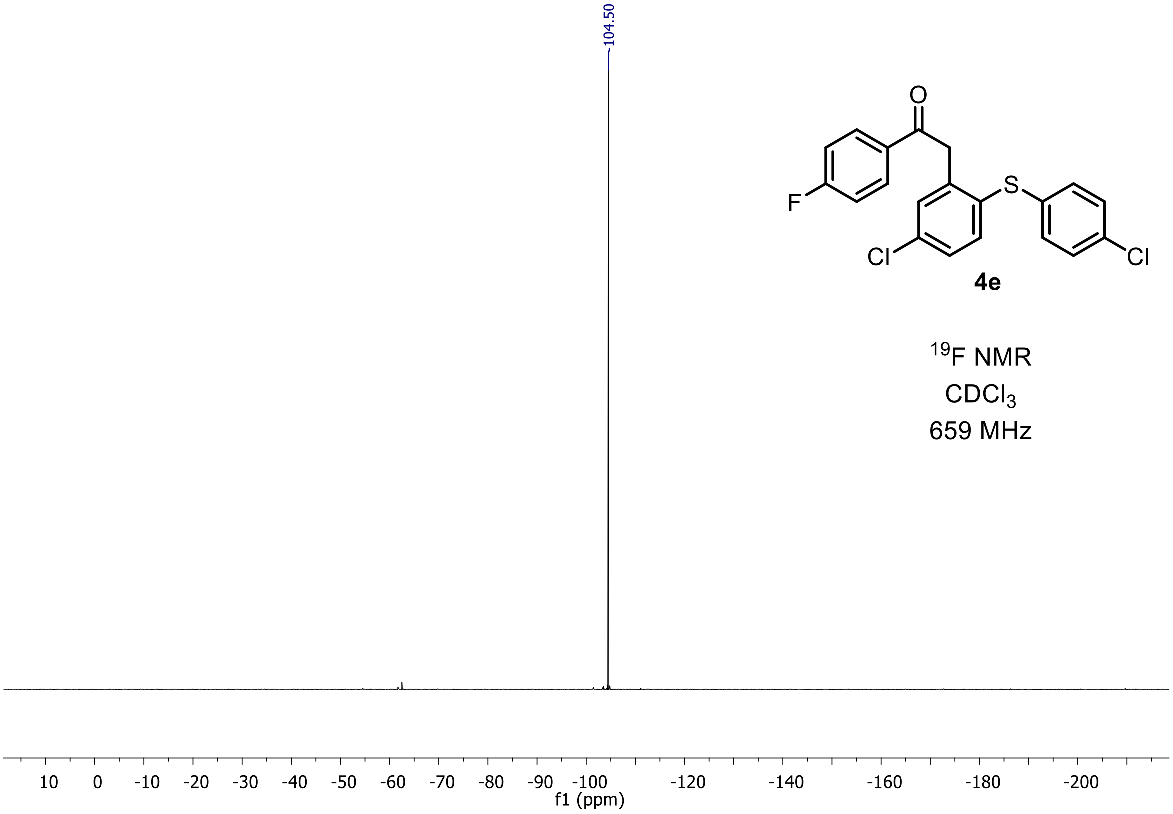
104.50
O
F
S
Cl
Cl
4e
19F NMR
CDCl3
659 MHz
10 0 -10 -20 -30 -40 -50 -60 -70 -80 -90 -100 -120 -140 -160 -180 -200
f1 (ppm)

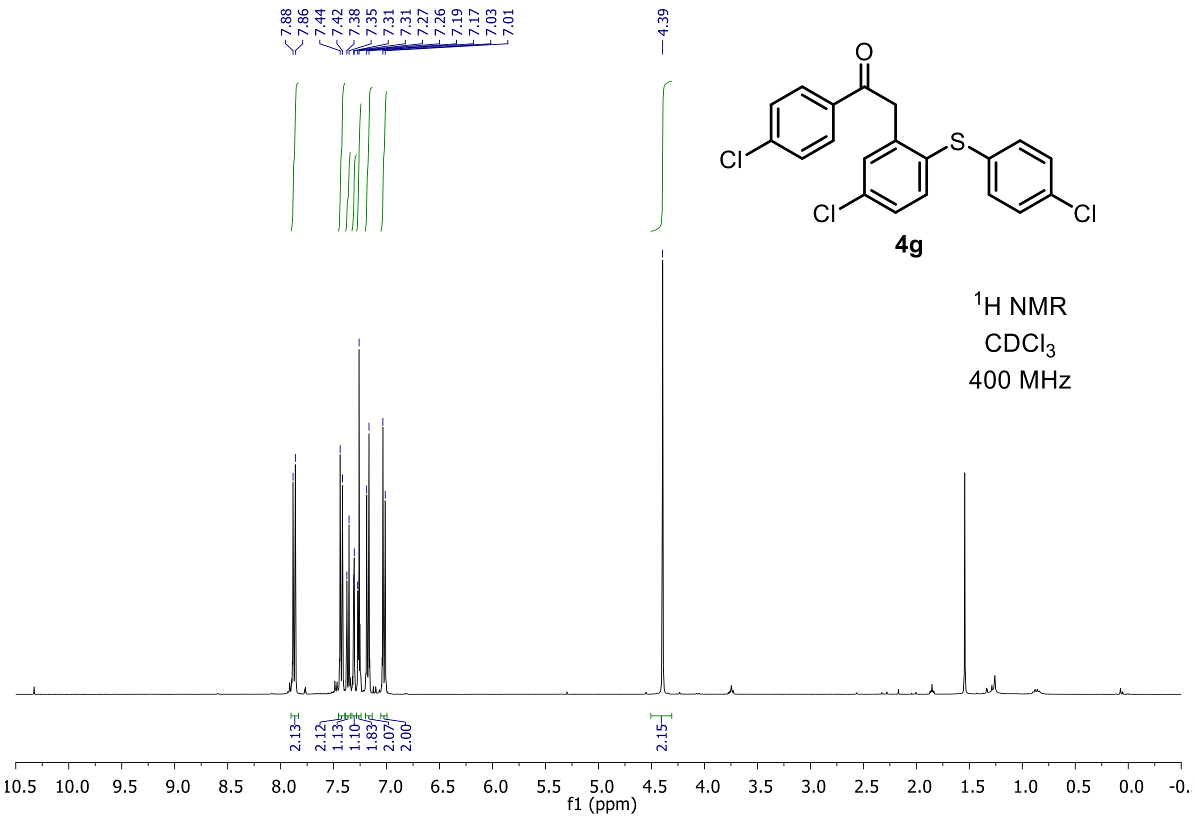
7.88
7.86
7.44
7.42
7.38
7.35
7.31
7.31
7.27
7.26
7.19
7.17
7.03
7.01
4.39
O
S
Cl
Cl
Cl
4g
1H NMR
CDCl3
400 MHz
2.13
2.12
1.13
1.10
1.83
2.07
2.00
2.15
f1 (ppm)

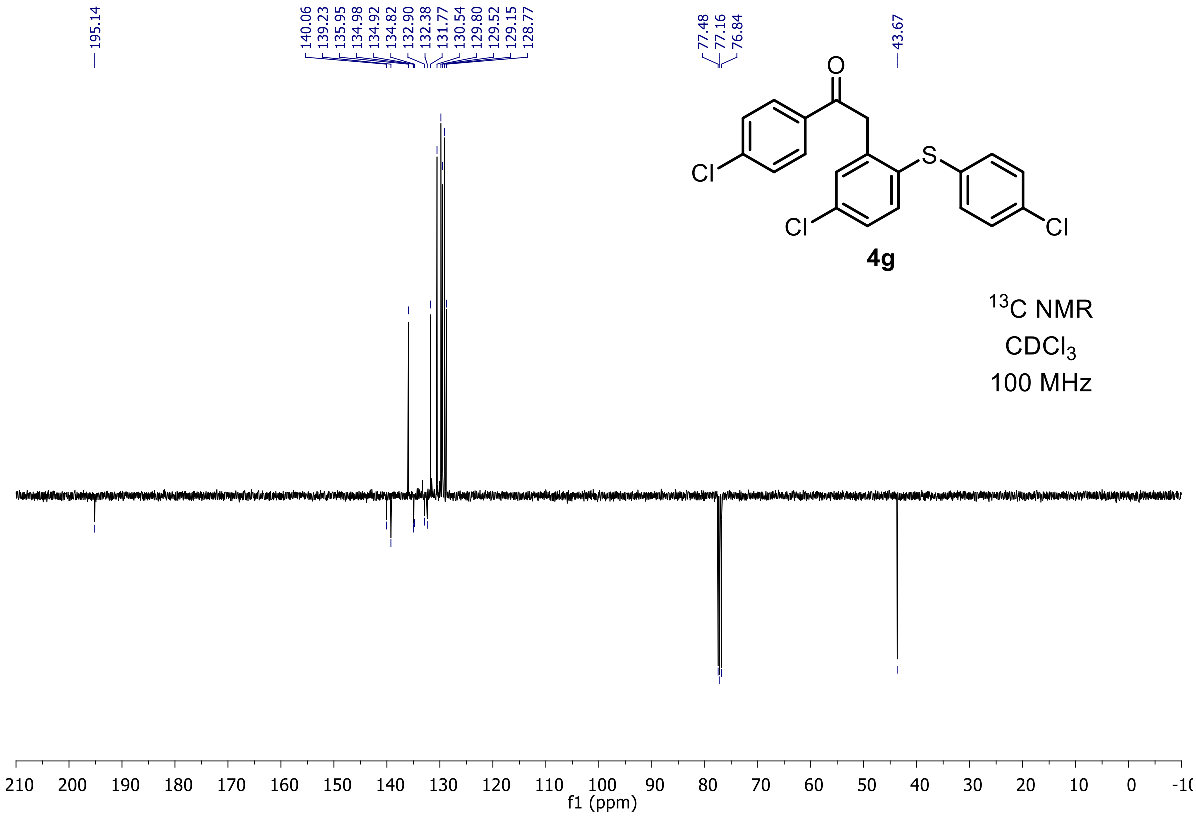
195.14
140.06
139.23
135.95
134.98
134.92
134.82
132.90
132.38
131.77
130.54
129.80
129.52
129.15
128.77
77.48
77.16
76.84
43.67
O
S
Cl
Cl
Cl
4g
13C NMR
CDCl3
100 MHz
f1 (ppm)

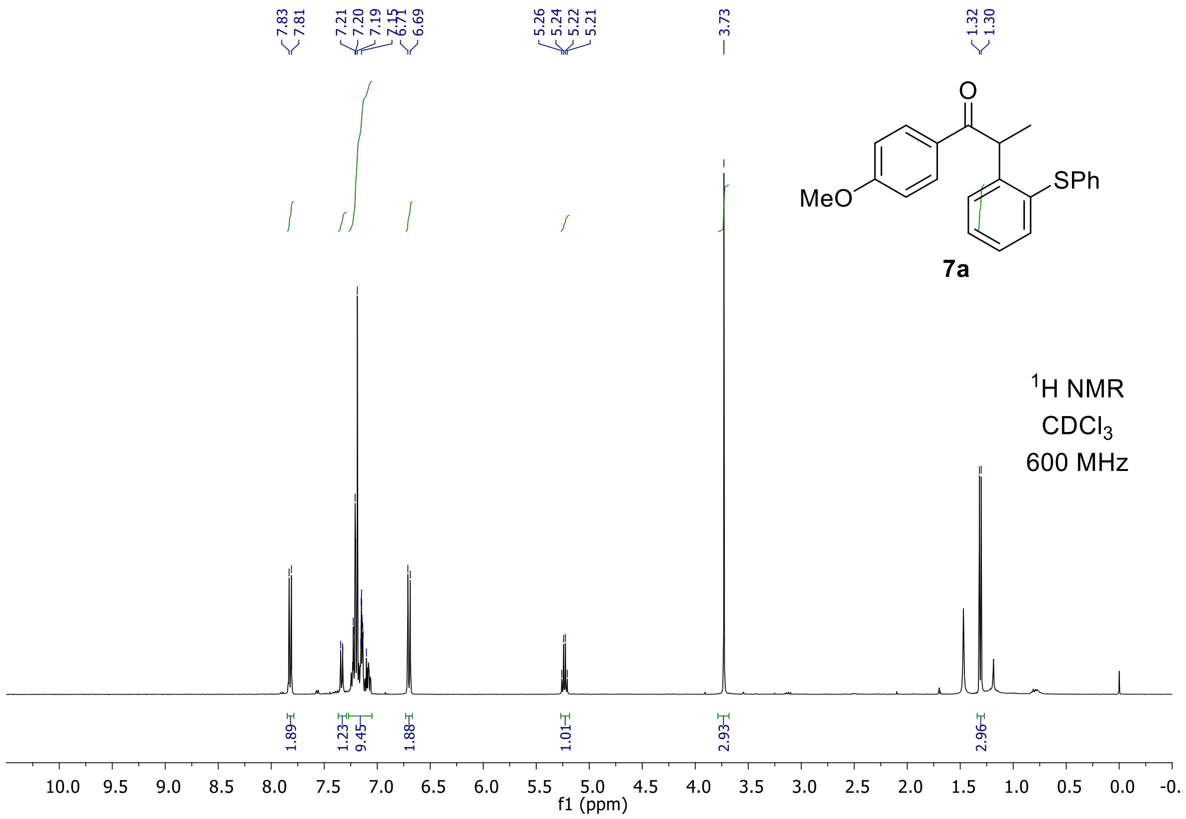
7.83
7.81
7.21
7.20
7.19
7.15
6.71
6.69
5.26
5.24
5.22
5.21
3.73
1.32
1.30
O
MeO
SPh
7a
1H NMR
CDCl3
600 MHz
1.89
1.23
9.45
1.88
1.01
2.93
2.96
f1 (ppm)

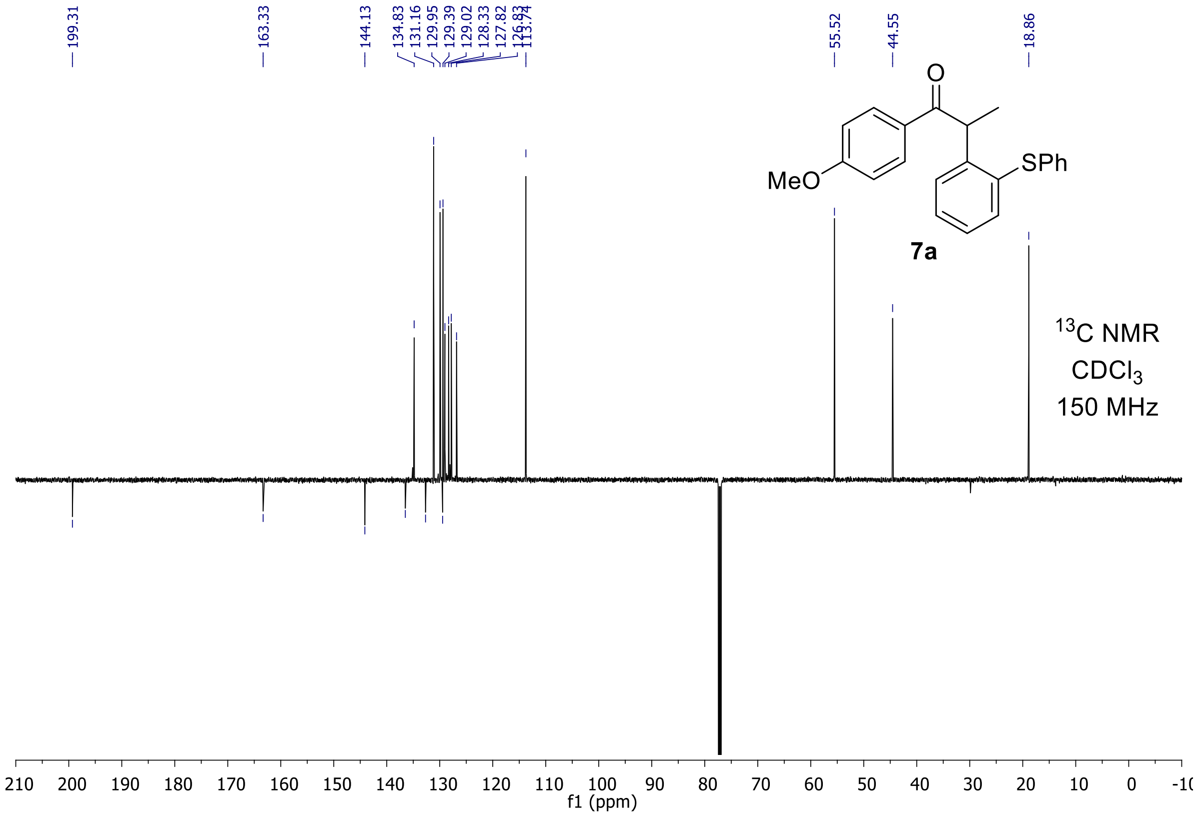
199.31
163.33
144.13
134.83
131.16
129.95
129.39
129.02
128.33
127.82
126.93
113.74
55.52
44.55
18.86
O
MeO
SPh
7a
13C NMR
CDCl3
150 MHz
f1 (ppm)

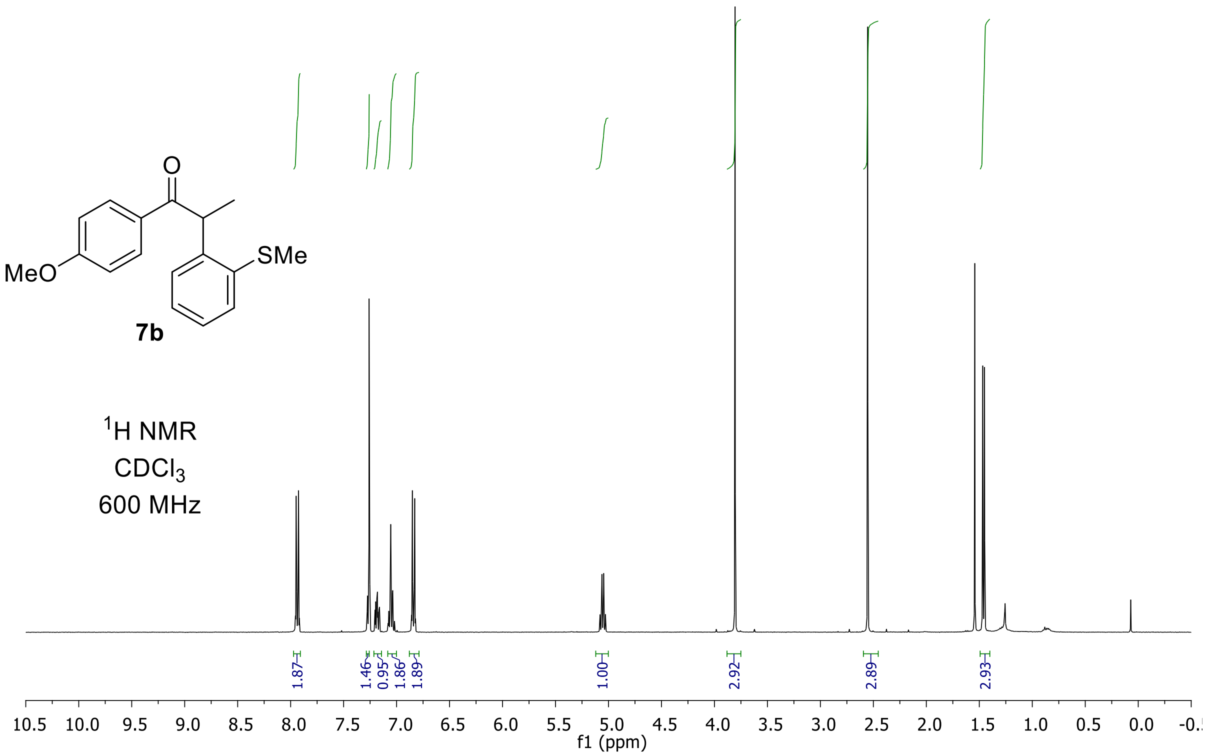
MeO
SMe
O
7b
¹H NMR
CDCl3
600 MHz
1.87
1.46
0.95
1.86
1.89
1.00
2.92
2.89
2.93
10.5
10.0
9.5
9.0
8.5
8.0
7.5
7.0
6.5
6.0
5.5
5.0
4.5
4.0
3.5
3.0
2.5
2.0
1.5
1.0
0.5
0.0
f1 (ppm)

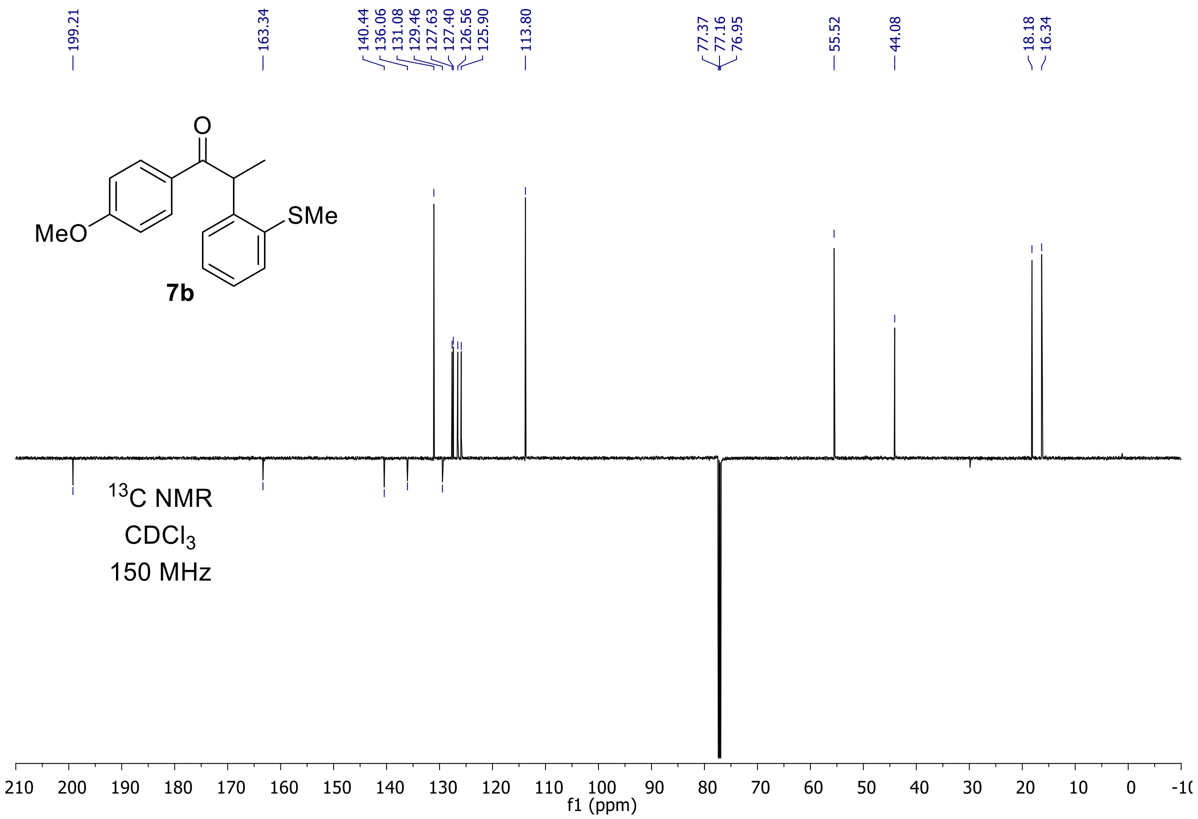
199.21
163.34
140.44
136.06
131.08
129.46
127.63
127.40
126.56
125.90
113.80
77.37
77.16
76.95
55.52
44.08
18.18
16.34
MeO
SMe
O
7b
¹³C NMR
CDCl3
150 MHz
210
200
190
180
170
160
150
140
130
120
110
100
90
80
70
60
50
40
30
20
10
0
f1 (ppm)

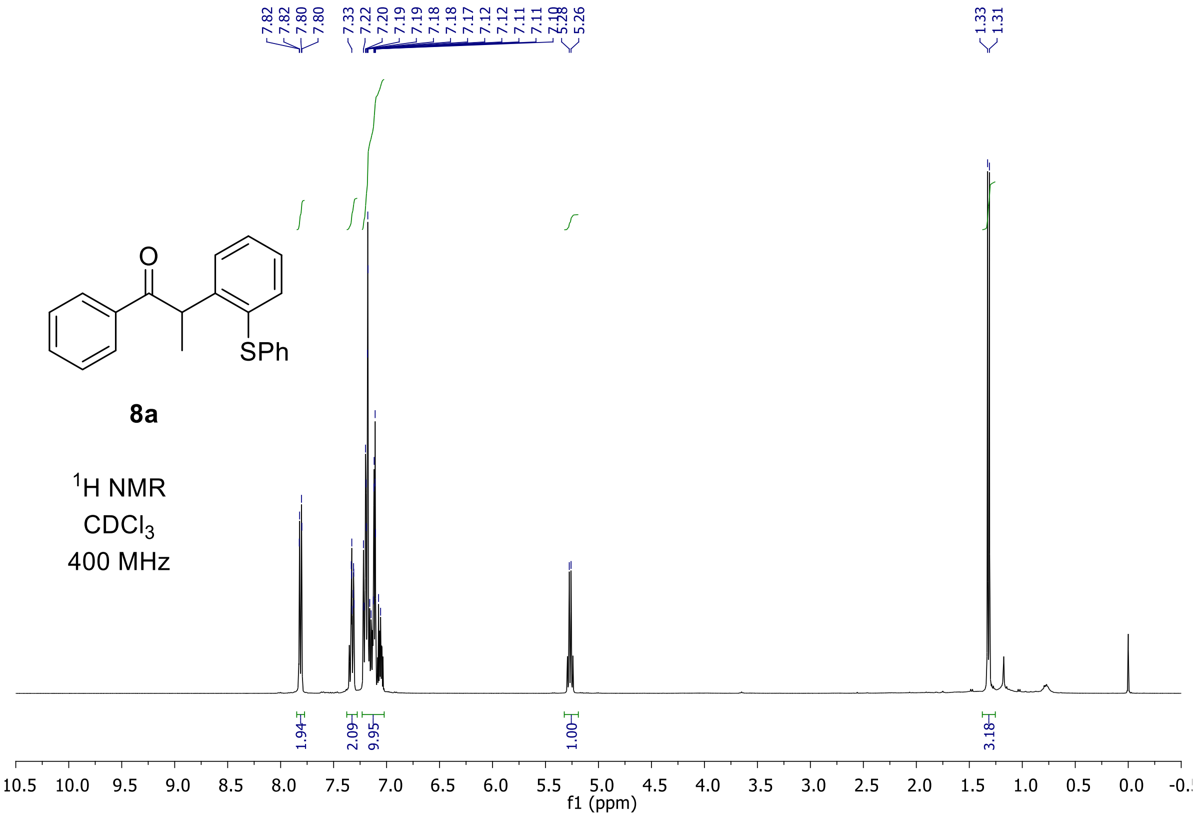
7.82
7.82
7.80
7.80
7.33
7.22
7.20
7.19
7.19
7.18
7.18
7.17
7.12
7.12
7.11
7.11
7.10
5.28
5.26
1.33
1.31
O
SPh
8a
1H NMR
CDCl3
400 MHz
1.94
2.09
9.95
1.00
3.18
10.5 10.0 9.5 9.0 8.5 8.0 7.5 7.0 6.5 6.0 5.5 5.0 4.5 4.0 3.5 3.0 2.5 2.0 1.5 1.0 0.5 0.0
f1 (ppm)

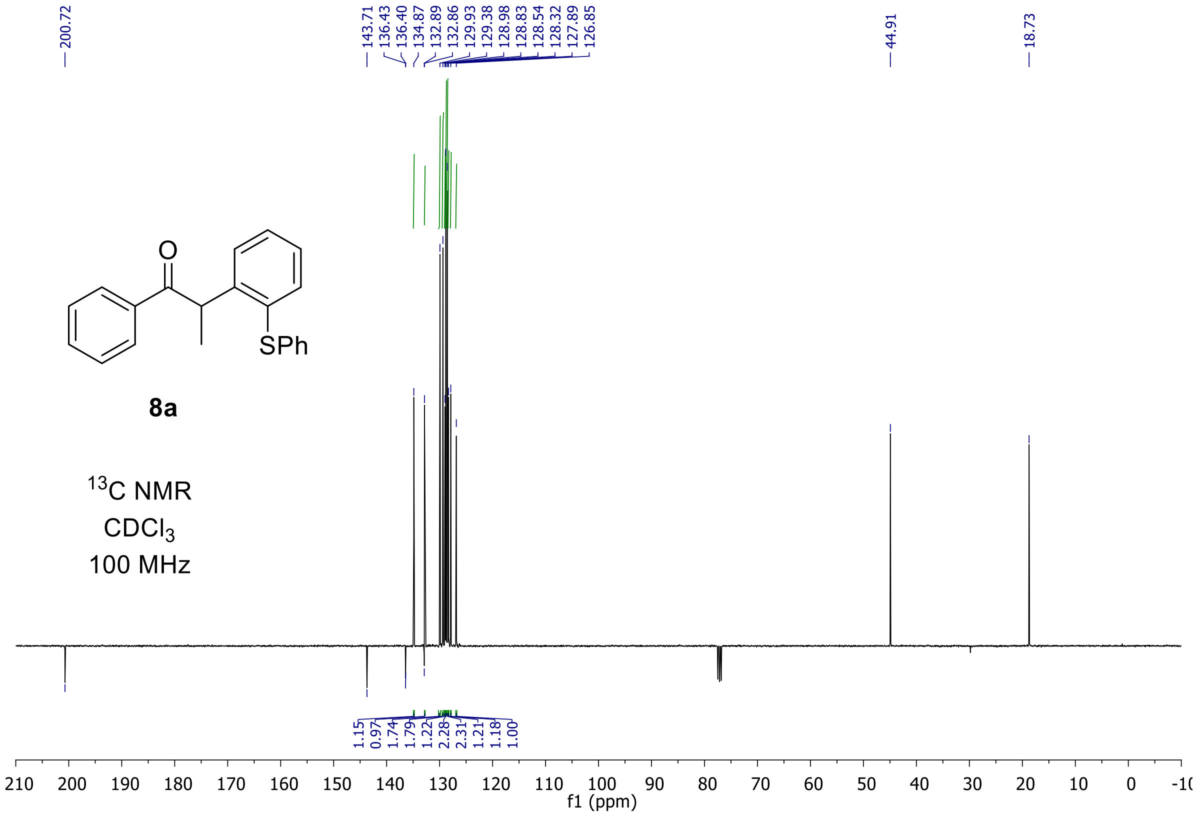
200.72
143.71
136.43
136.40
134.87
132.89
132.86
129.93
129.38
128.98
128.83
128.54
128.32
127.89
126.85
44.91
18.73
O
SPh
8a
13C NMR
CDCl3
100 MHz
1.15
0.97
1.74
1.79
1.22
2.28
2.31
1.21
1.18
1.00
210 200 190 180 170 160 150 140 130 120 110 100 90 80 70 60 50 40 30 20 10 0
f1 (ppm)

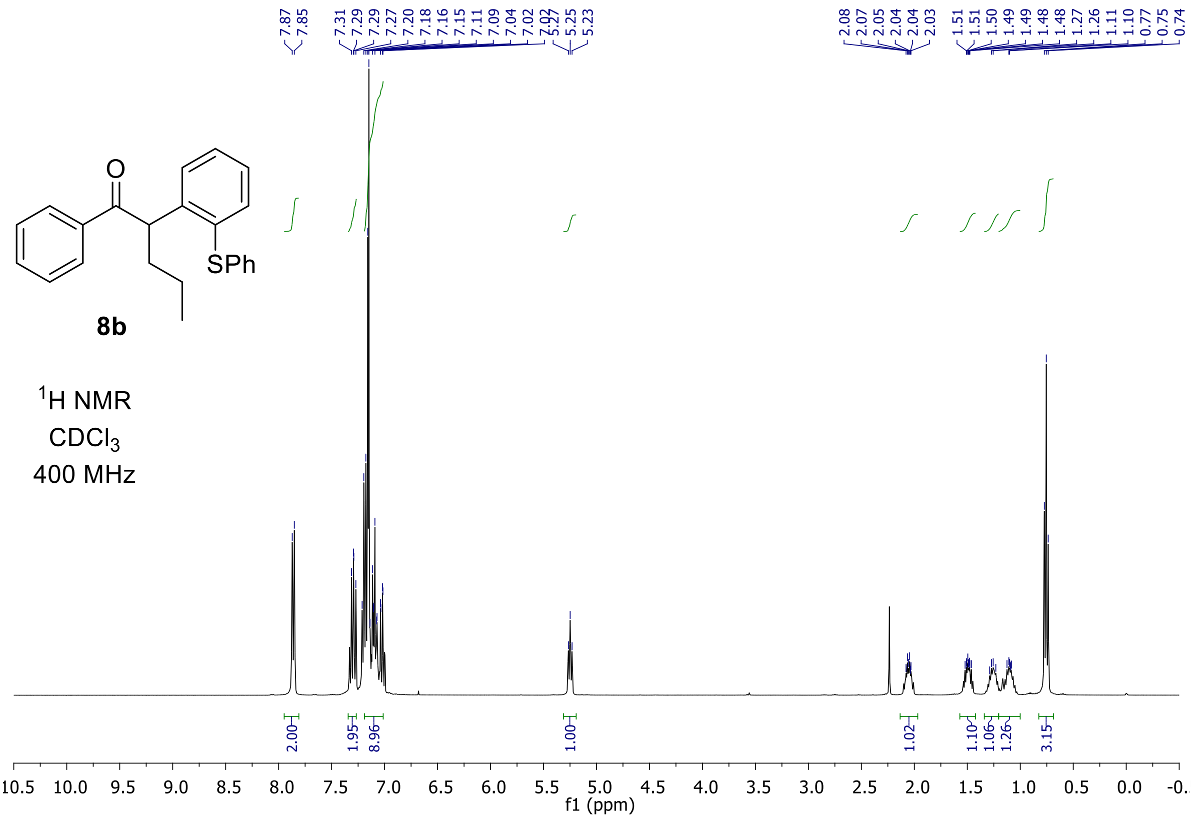
8b
^{1}H NMR
$CDCl_3$
400 MHz
SPh
f1 (ppm)

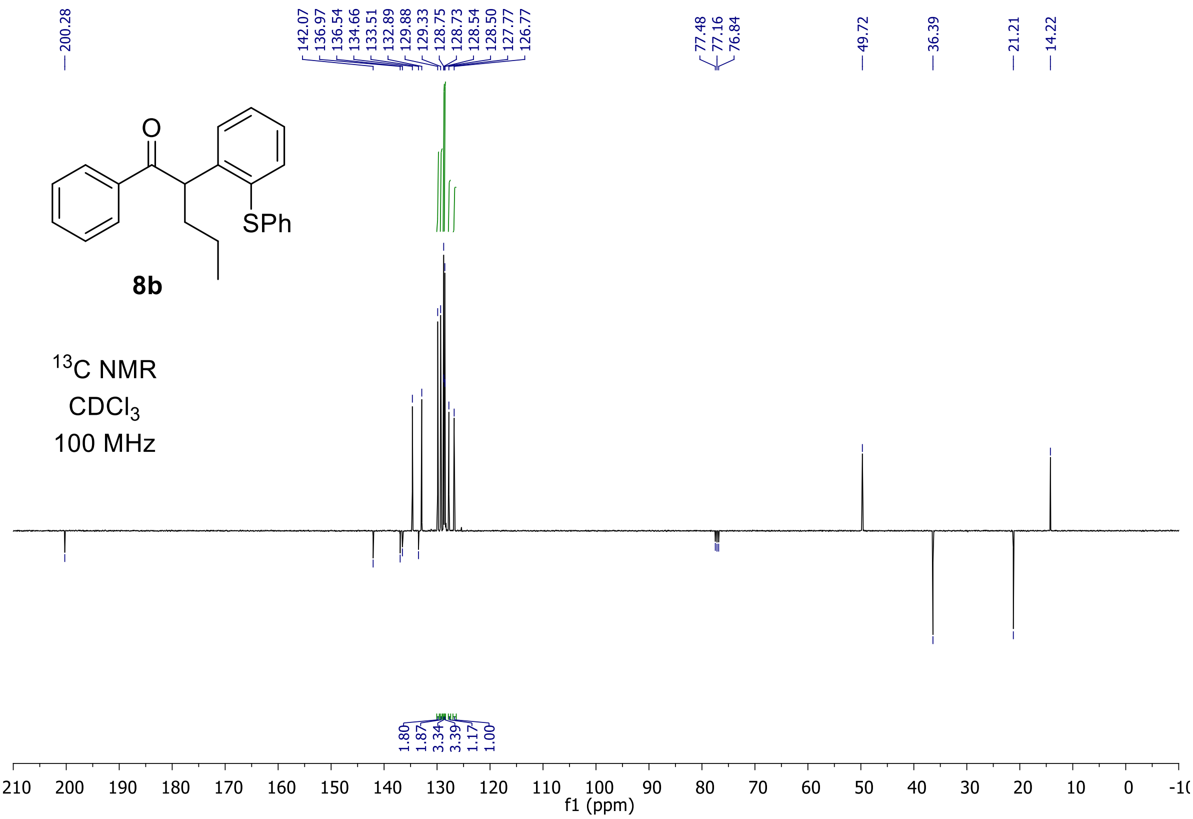
8b
^{13}C NMR
$CDCl_3$
100 MHz
SPh
f1 (ppm)

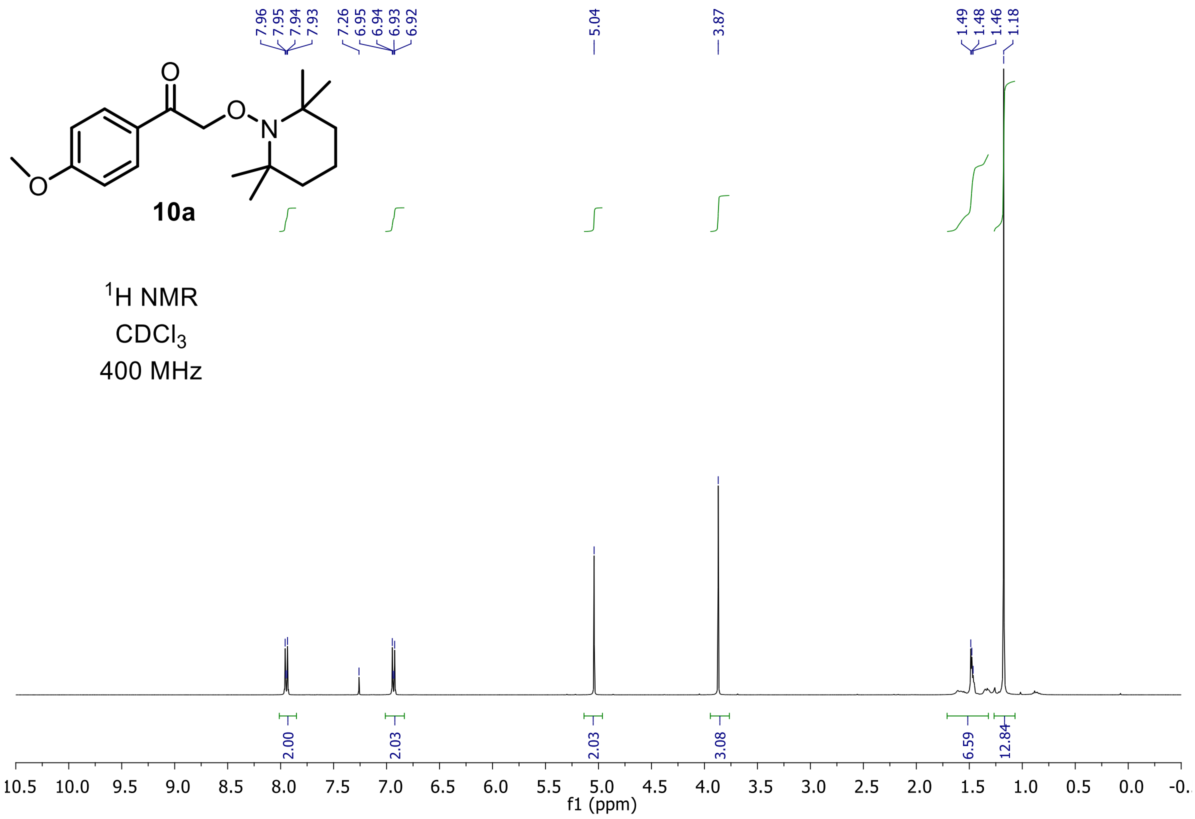
7.96
7.95
7.94
7.93
7.26
6.95
6.94
6.93
6.92
5.04
3.87
1.49
1.48
1.46
1.18
O
O
N
O
10a
^{1}H NMR
$CDCl_3$
400 MHz
2.00
2.03
2.03
3.08
6.59
12.84
10.5 10.0 9.5 9.0 8.5 8.0 7.5 7.0 6.5 6.0 5.5 5.0 4.5 4.0 3.5 3.0 2.5 2.0 1.5 1.0 0.5 0.0
f1 (ppm)

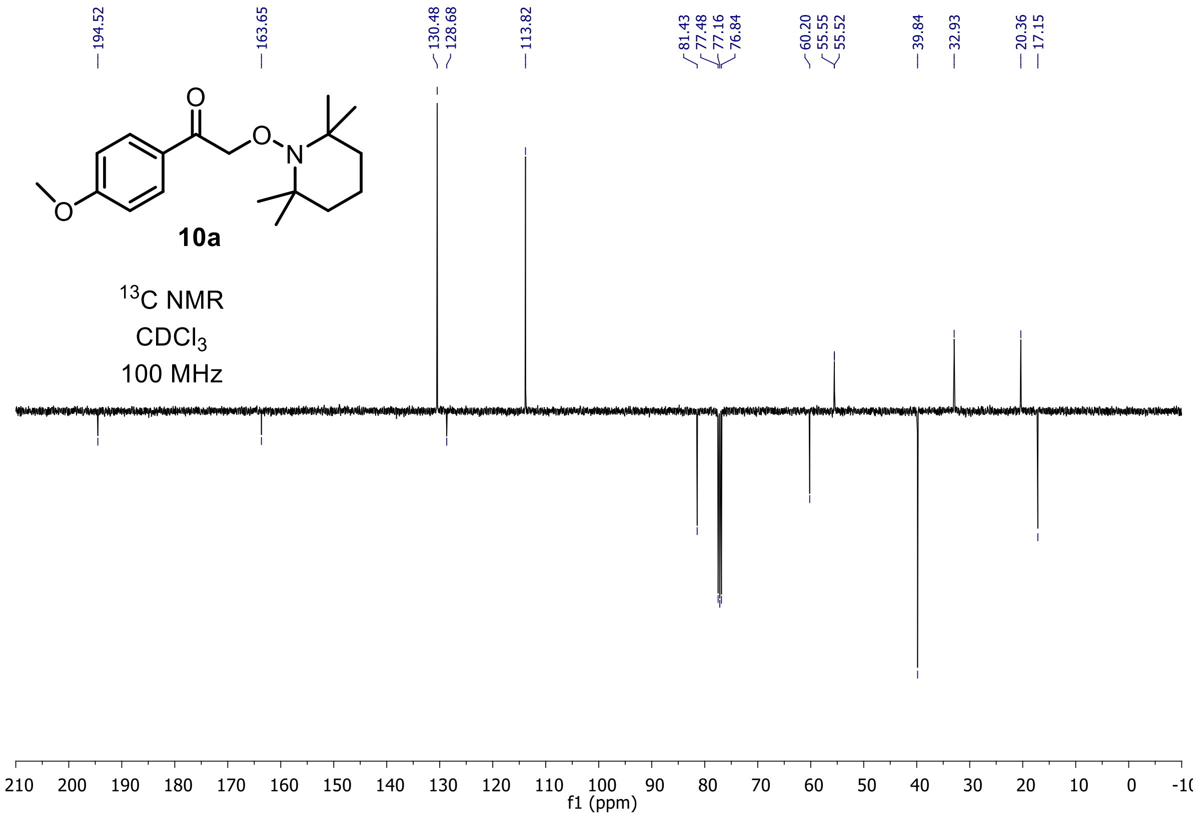
194.52
163.65
130.48
128.68
113.82
81.43
77.48
77.16
76.84
60.20
55.55
55.52
39.84
32.93
20.36
17.15
O
O
N
O
10a
^{13}C NMR
$CDCl_3$
100 MHz
210 200 190 180 170 160 150 140 130 120 110 100 90 80 70 60 50 40 30 20 10 0
f1 (ppm)

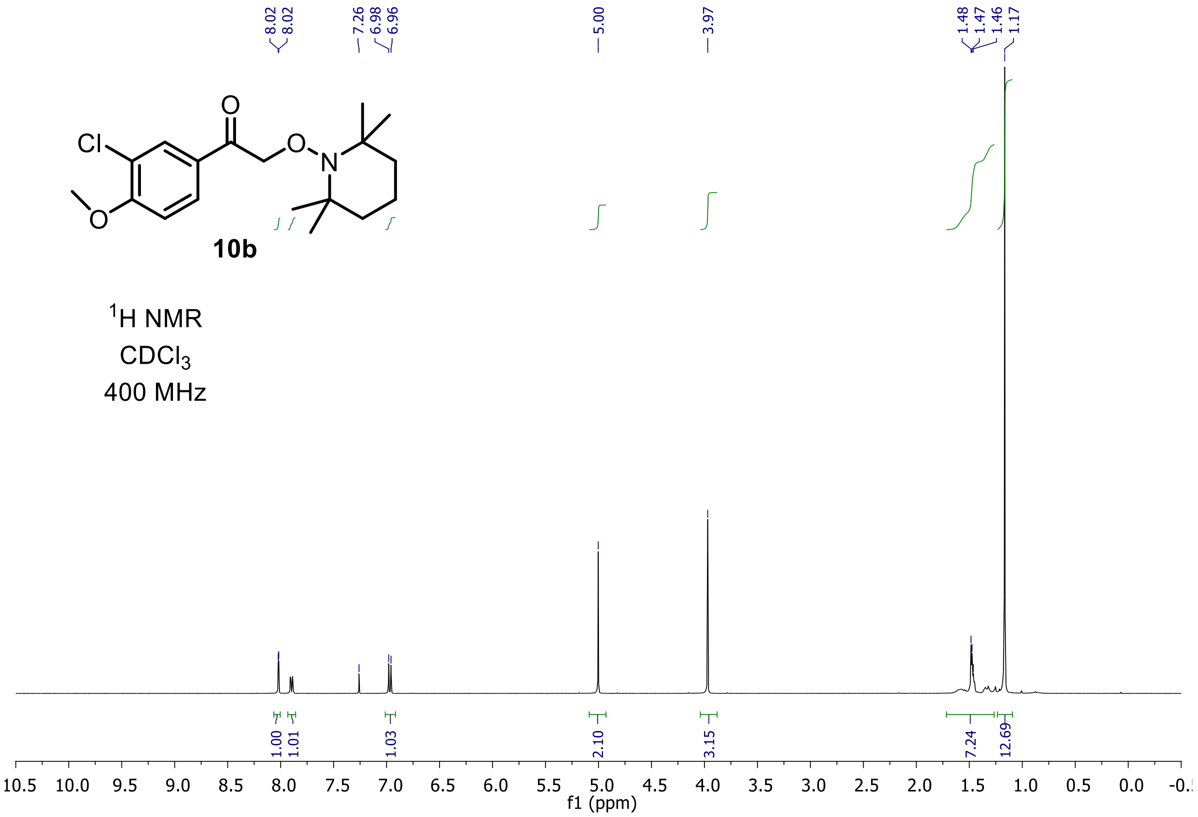
8.02
8.02
7.26
6.98
6.96
5.00
3.97
1.48
1.47
1.46
1.17
Cl
O
O
N
O
10b
1H NMR
CDCl3
400 MHz
1.00
1.01
1.03
2.10
3.15
7.24
12.69
10.5 10.0 9.5 9.0 8.5 8.0 7.5 7.0 6.5 6.0 5.5 5.0 4.5 4.0 3.5 3.0 2.5 2.0 1.5 1.0 0.5 0.0
f1 (ppm)

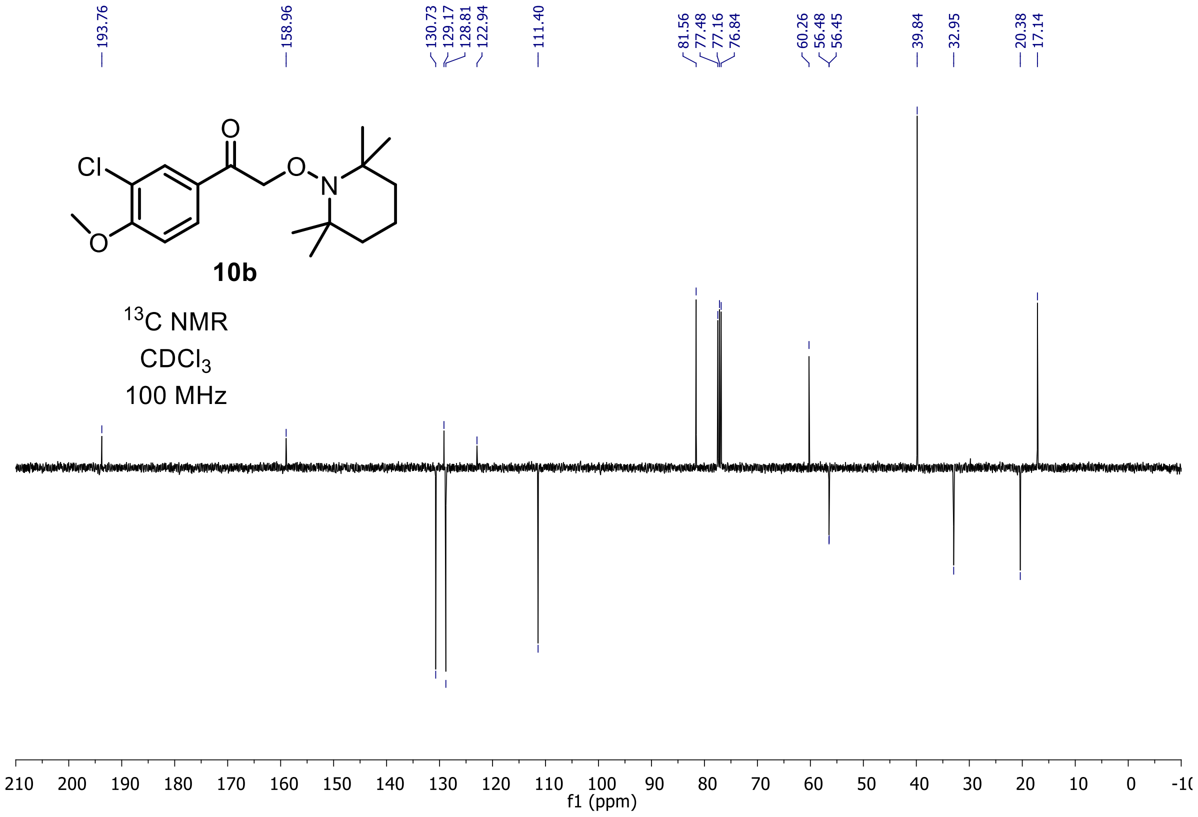
193.76
158.96
130.73
129.17
128.81
122.94
111.40
81.56
77.48
77.16
76.84
60.26
56.48
56.45
39.84
32.95
20.38
17.14
Cl
O
O
N
O
10b
13C NMR
CDCl3
100 MHz
210 200 190 180 170 160 150 140 130 120 110 100 90 80 70 60 50 40 30 20 10 0
f1 (ppm)

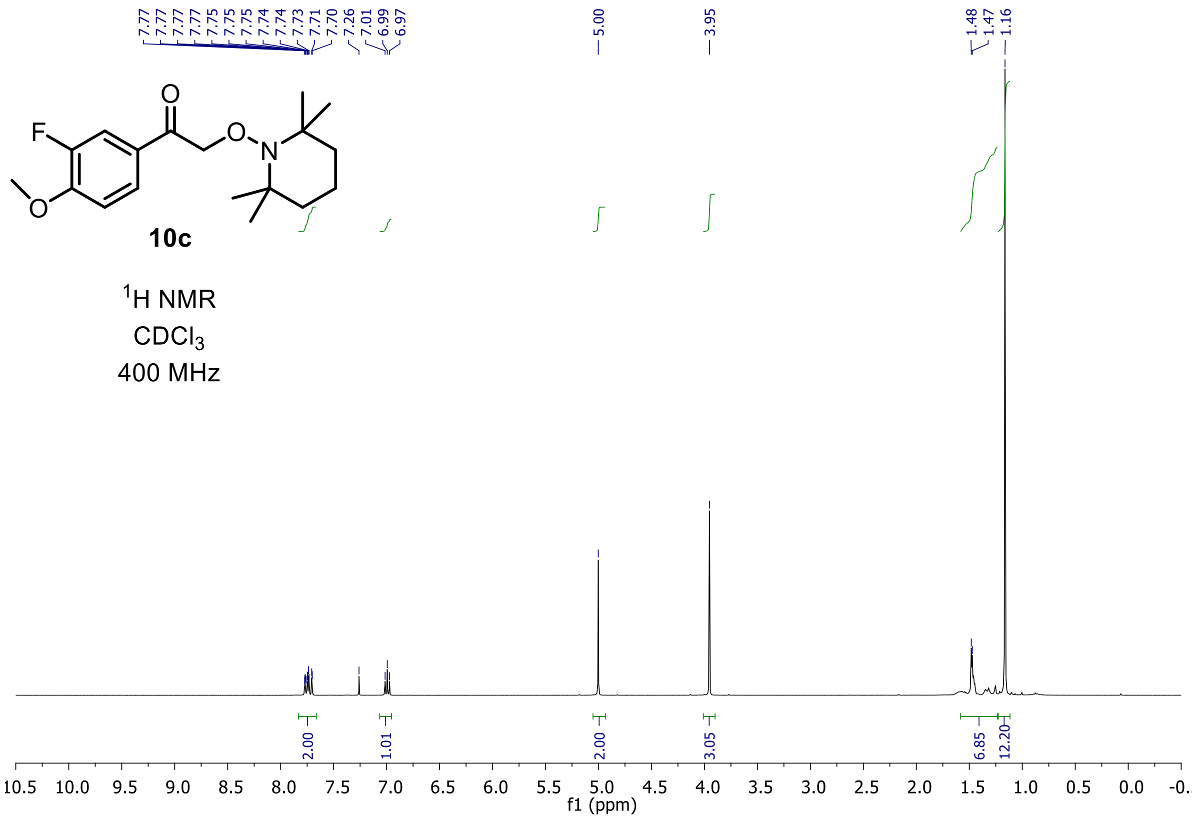
7.77
7.77
7.77
7.77
7.75
7.75
7.75
7.74
7.74
7.73
7.71
7.70
7.26
7.01
6.99
6.97
5.00
3.95
1.48
1.47
1.16
F
O
O
N
10c
^{1}H NMR
$CDCl_3$
400 MHz
2.00
1.01
2.00
3.05
6.85
12.20
f1 (ppm)

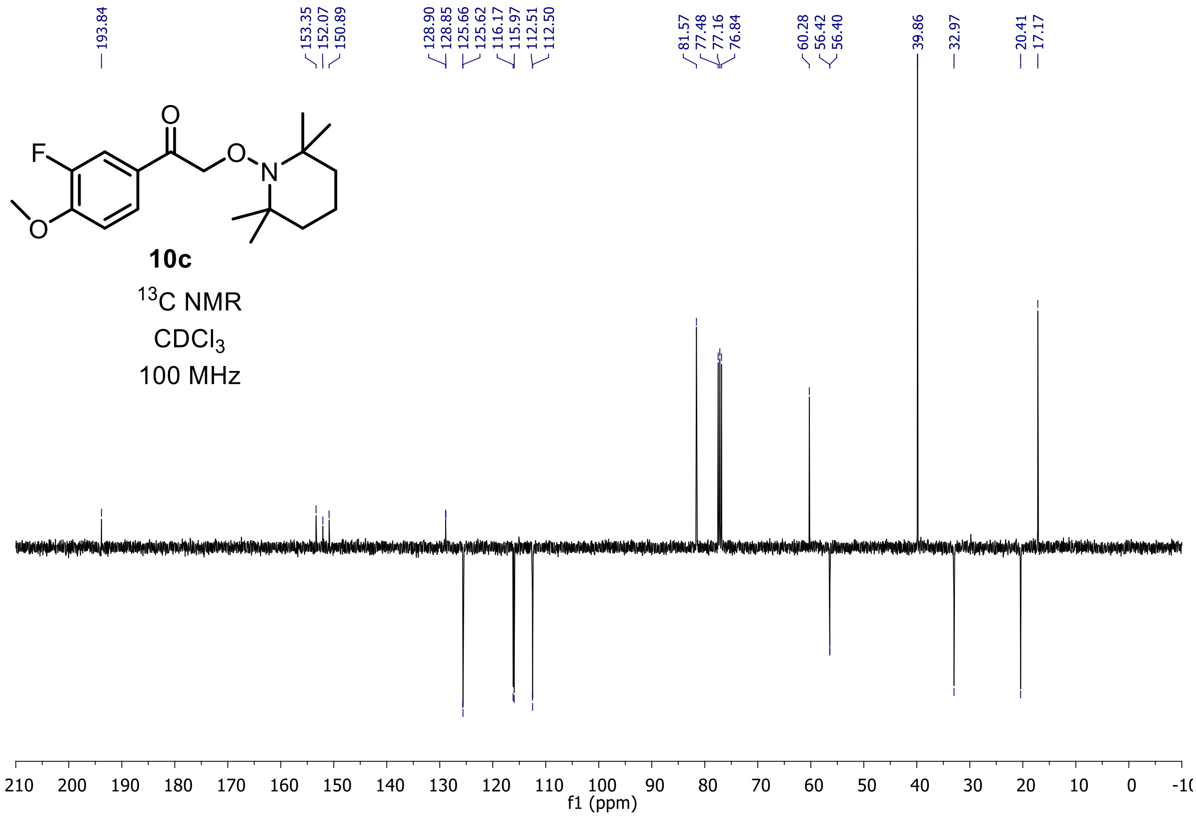
193.84
153.35
152.07
150.89
128.90
128.85
125.66
125.62
116.17
115.97
112.51
112.50
81.57
77.48
77.16
76.84
60.28
56.42
56.40
39.86
32.97
20.41
17.17
F
O
O
N
10c
^{13}C NMR
$CDCl_3$
100 MHz
f1 (ppm)

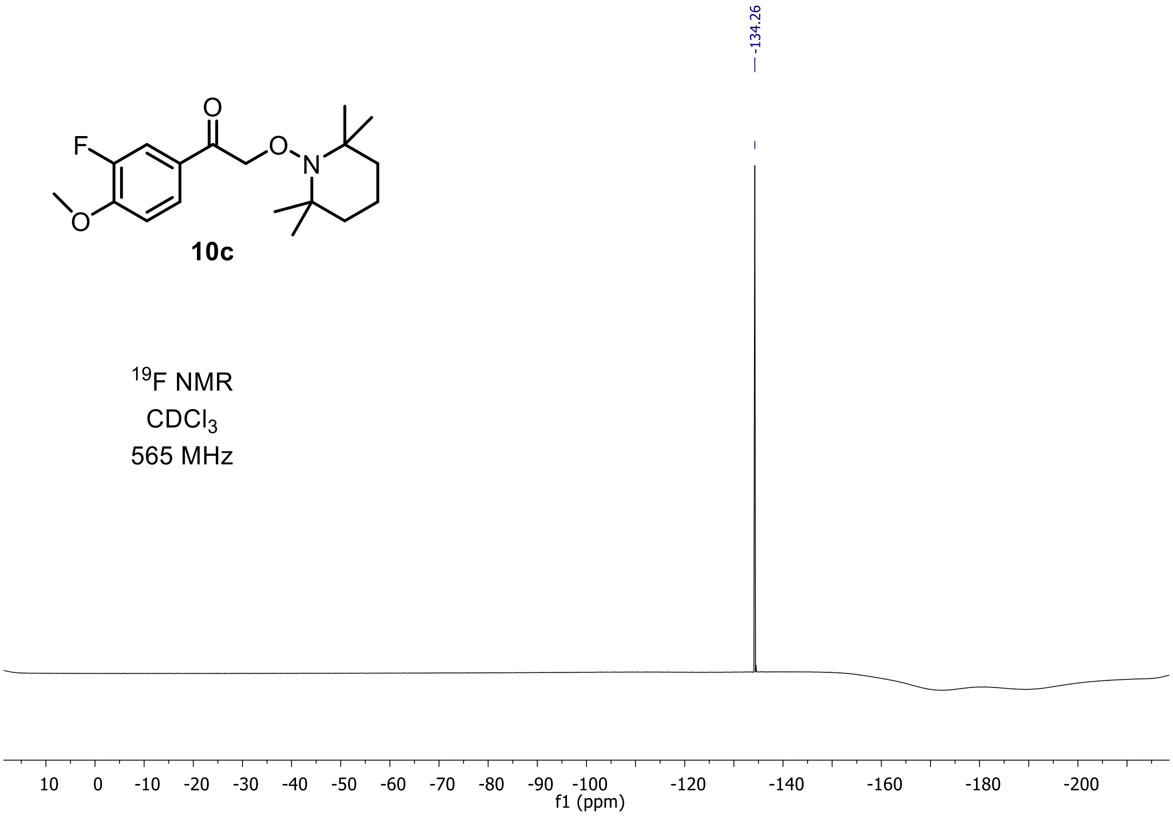
-134.26
F
O
O
N
O
10c
19F NMR
CDCl3
565 MHz
10 0 -10 -20 -30 -40 -50 -60 -70 -80 -90 -100 -120 -140 -160 -180 -200
f1 (ppm)

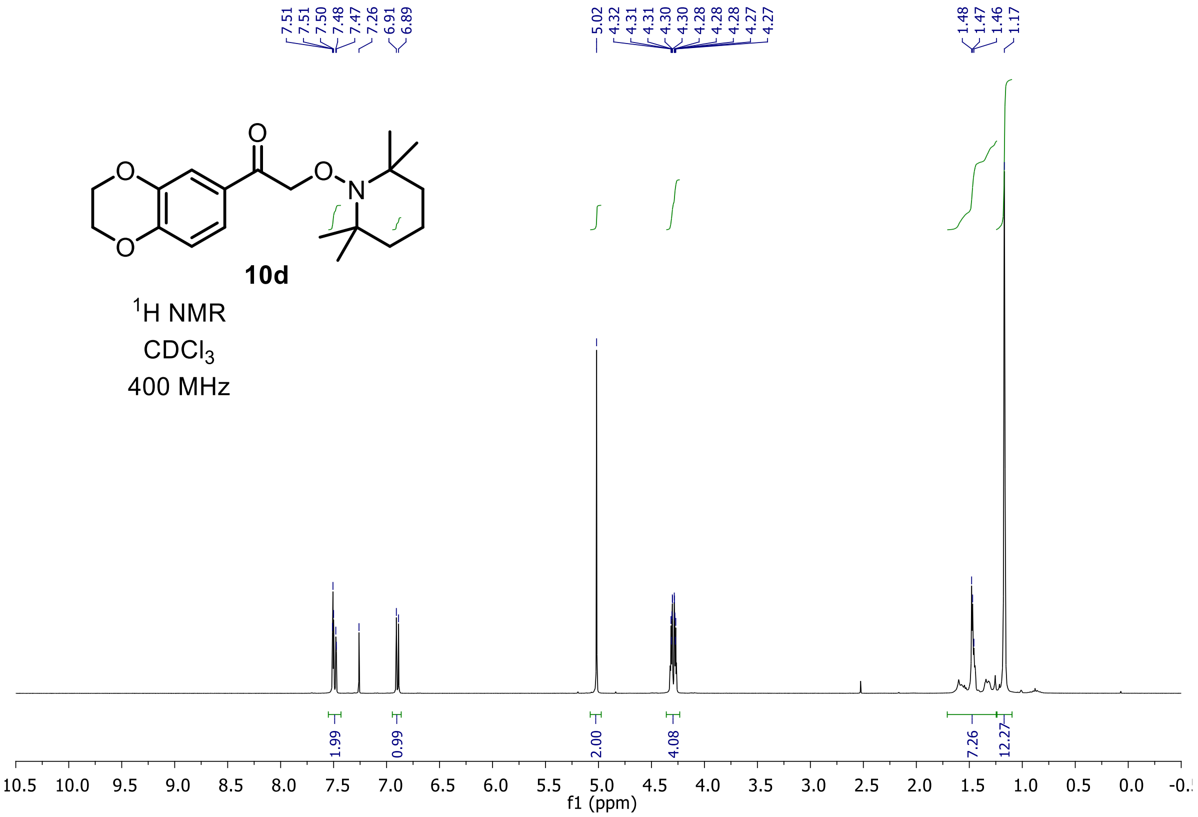
7.51
7.51
7.50
7.48
7.47
7.26
6.91
6.89
5.02
4.32
4.31
4.31
4.30
4.30
4.28
4.28
4.28
4.27
4.27
1.48
1.47
1.46
1.17
O
O
O
O
N
10d
1H NMR
CDCl3
400 MHz
1.99
0.99
2.00
4.08
7.26
12.27
f1 (ppm)

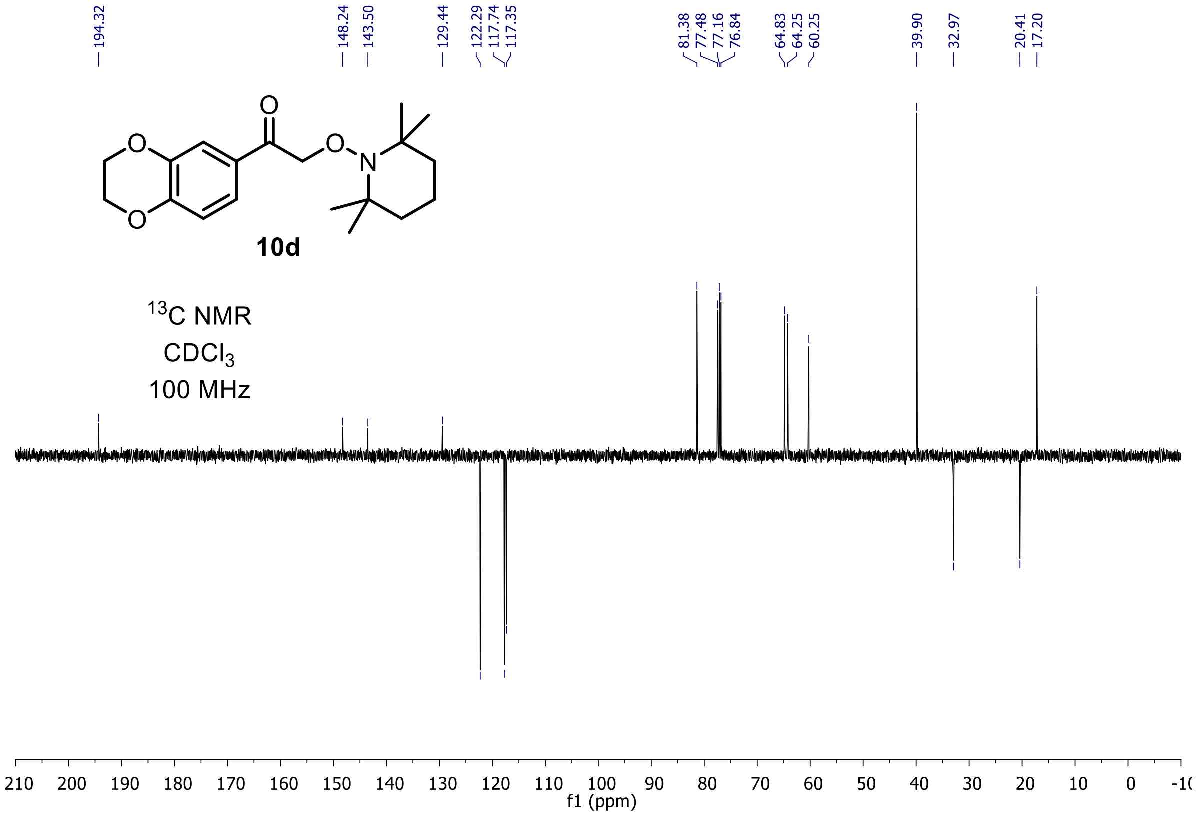
194.32
148.24
143.50
129.44
122.29
117.74
117.35
81.38
77.48
77.16
76.84
64.83
64.25
60.25
39.90
32.97
20.41
17.20
O
O
O
O
N
10d
13C NMR
CDCl3
100 MHz
f1 (ppm)

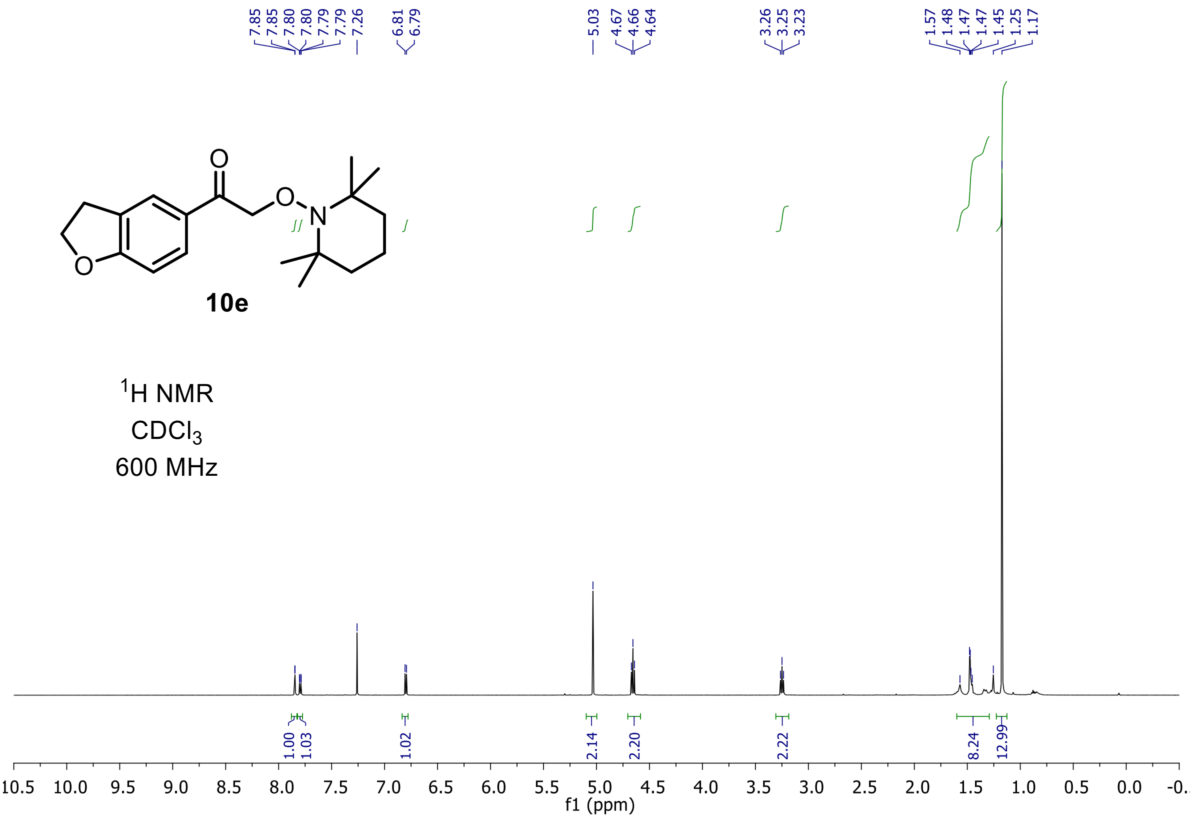

7.85
7.85
7.80
7.80
7.79
7.79
7.26
6.81
6.79
5.03
4.67
4.66
4.64
3.26
3.25
3.23
1.57
1.48
1.47
1.47
1.45
1.25
1.17
O
O
O
N
10e
1H NMR
CDCl3
600 MHz
1.00
1.03
1.02
2.14
2.20
2.22
8.24
12.99
10.5 10.0 9.5 9.0 8.5 8.0 7.5 7.0 6.5 6.0 5.5 5.0 4.5 4.0 3.5 3.0 2.5 2.0 1.5 1.0 0.5 0.0
f1 (ppm)

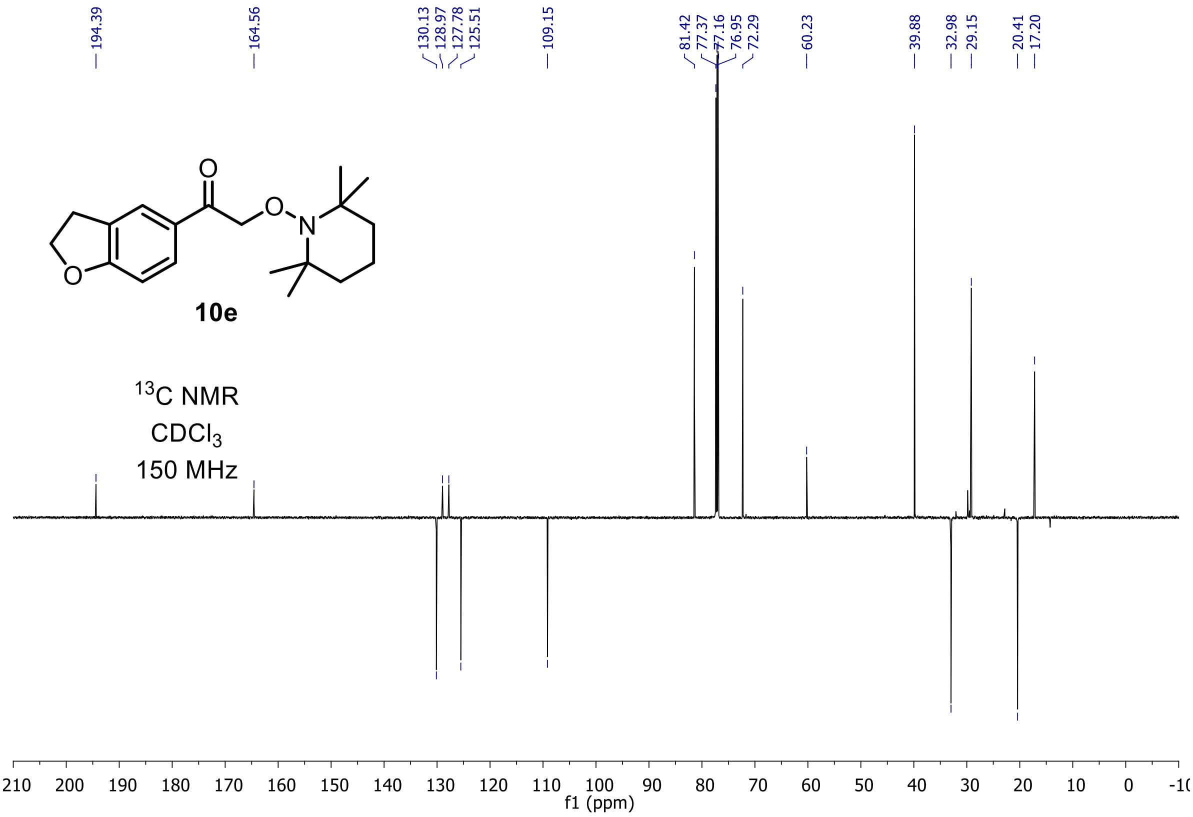

194.39
164.56
130.13
128.97
127.78
125.51
109.15
81.42
77.37
77.16
76.95
72.29
60.23
39.88
32.98
29.15
20.41
17.20
O
O
O
N
10e
13C NMR
CDCl3
150 MHz
210 200 190 180 170 160 150 140 130 120 110 100 90 80 70 60 50 40 30 20 10 0
f1 (ppm)

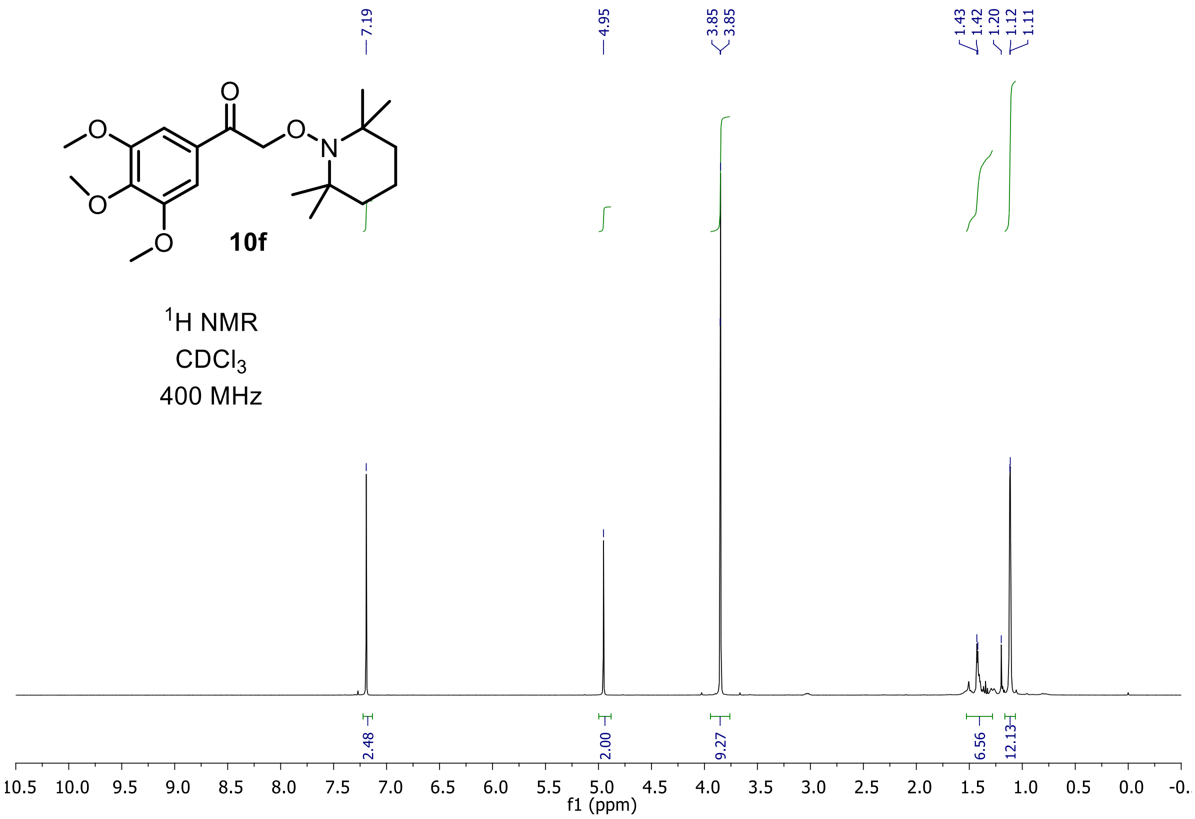
10f
1H NMR
CDCl3
400 MHz
7.19
4.95
3.85
3.85
1.43
1.42
1.20
1.12
1.11
2.48
2.00
9.27
6.56
12.13
f1 (ppm)

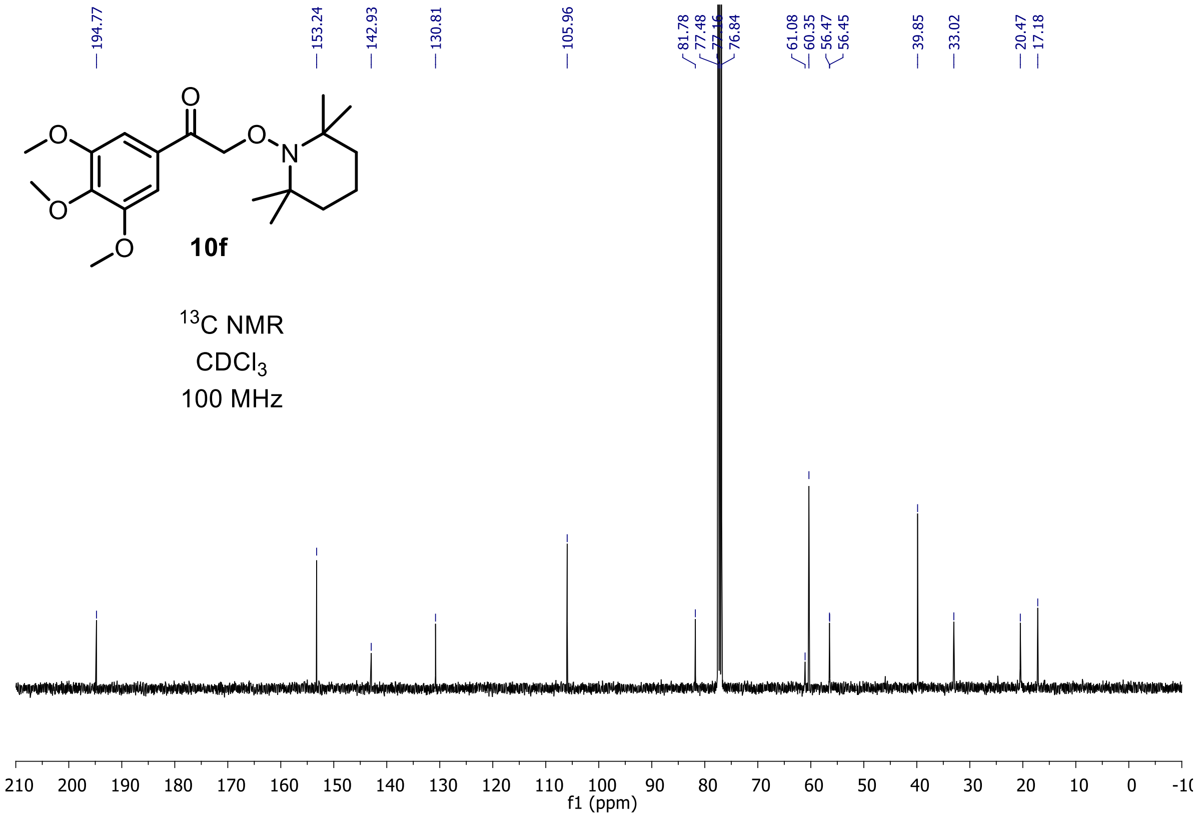
10f
13C NMR
CDCl3
100 MHz
194.77
153.24
142.93
130.81
105.96
81.78
77.48
76.84
61.08
60.35
56.47
56.45
39.85
33.02
20.47
17.18
f1 (ppm)

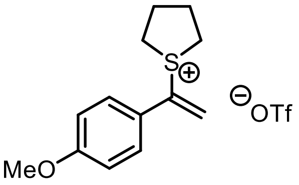

11

^{1}H NMR
$CDCl_3$
600 MHz

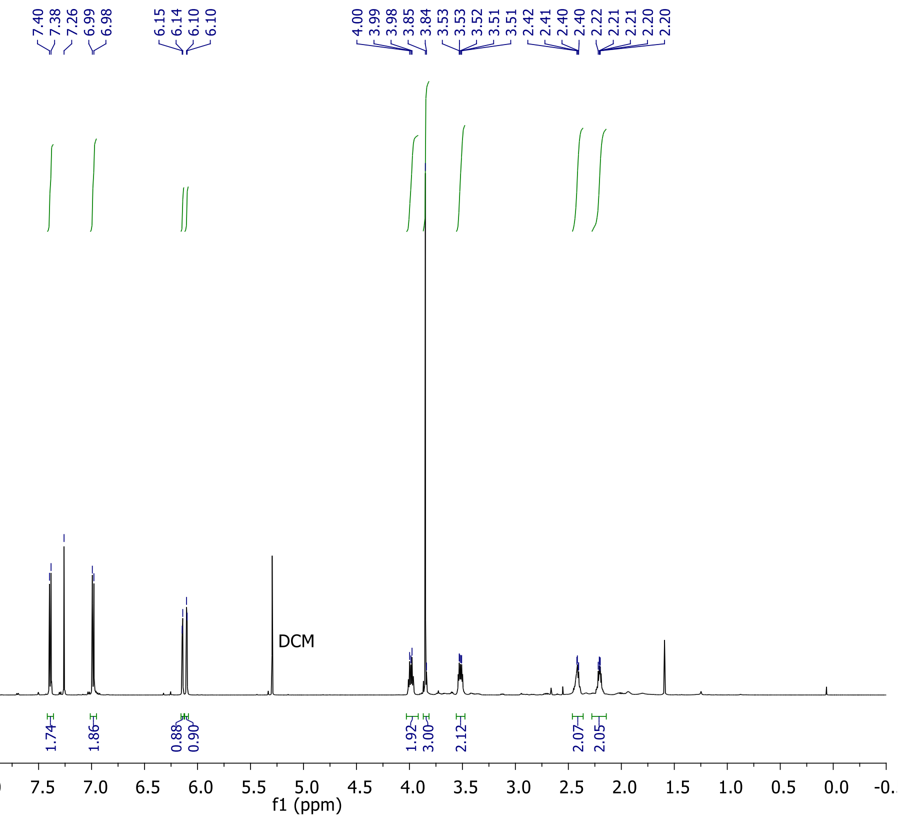

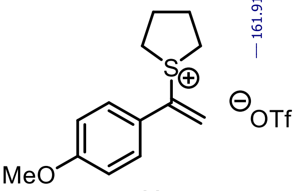

11

^{13}C NMR
$CDCl_3$
150 MHz

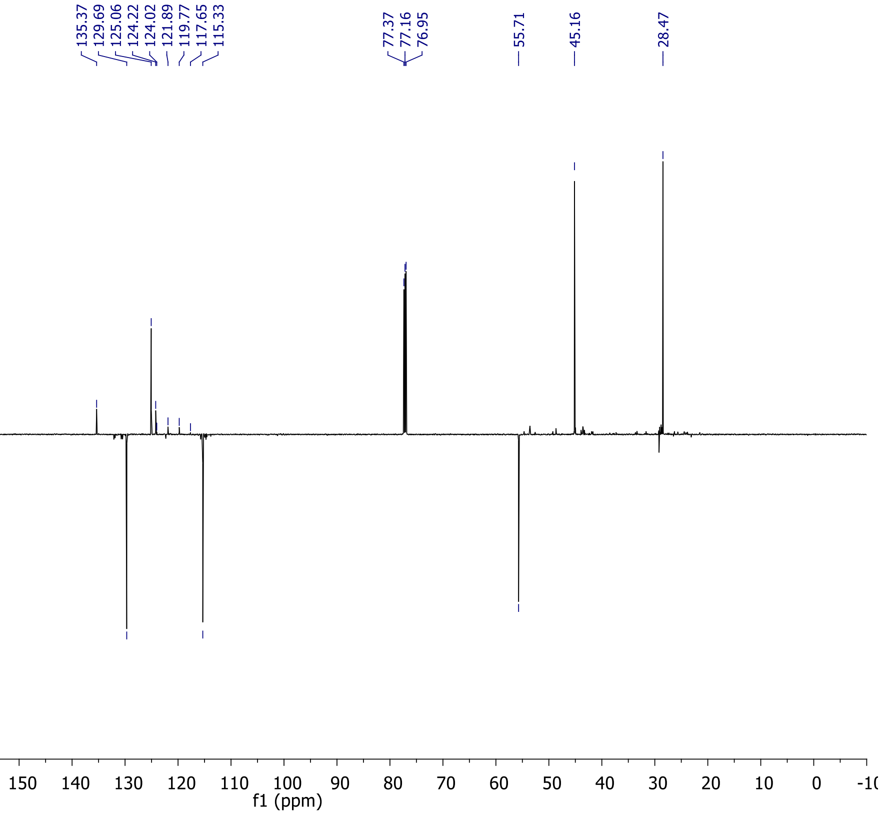

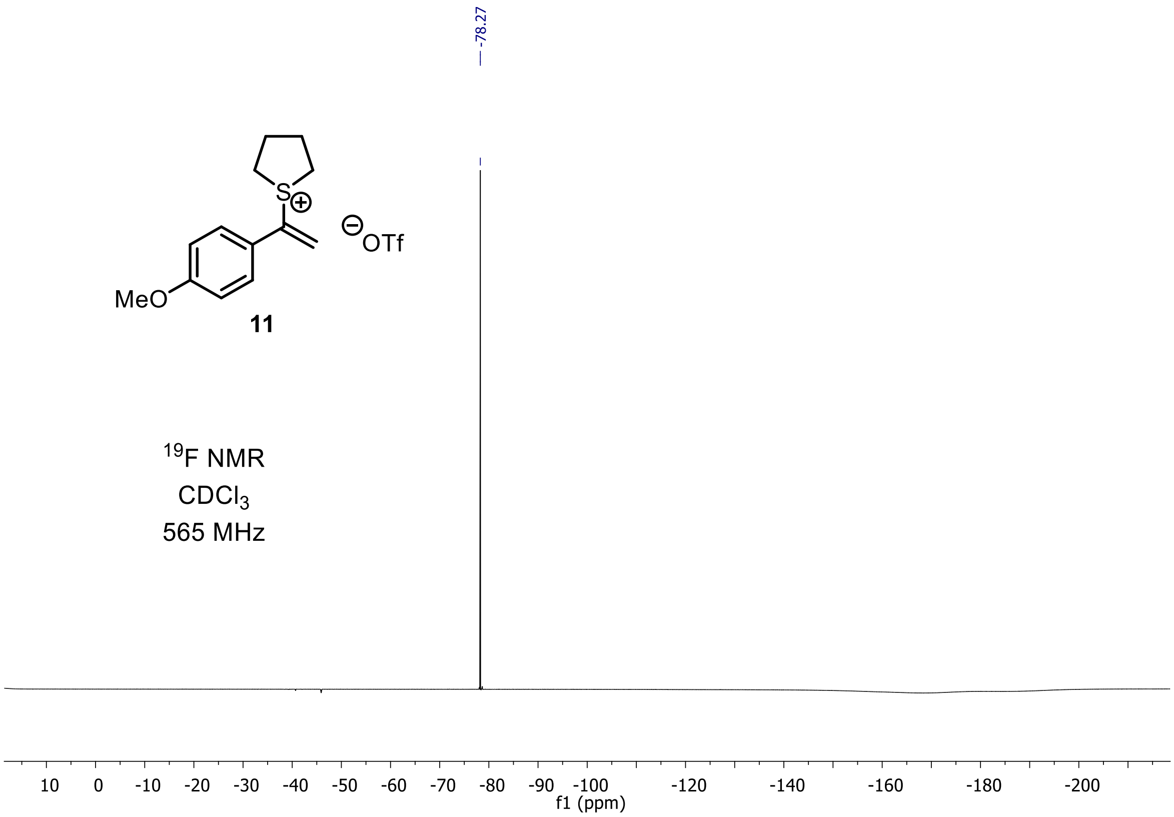

-78.27
S⊕
⊖OTf
MeO
11
^{19}F NMR
$CDCl_3$
565 MHz
10 0 -10 -20 -30 -40 -50 -60 -70 -80 -90 -100 -120 -140 -160 -180 -200
f1 (ppm)

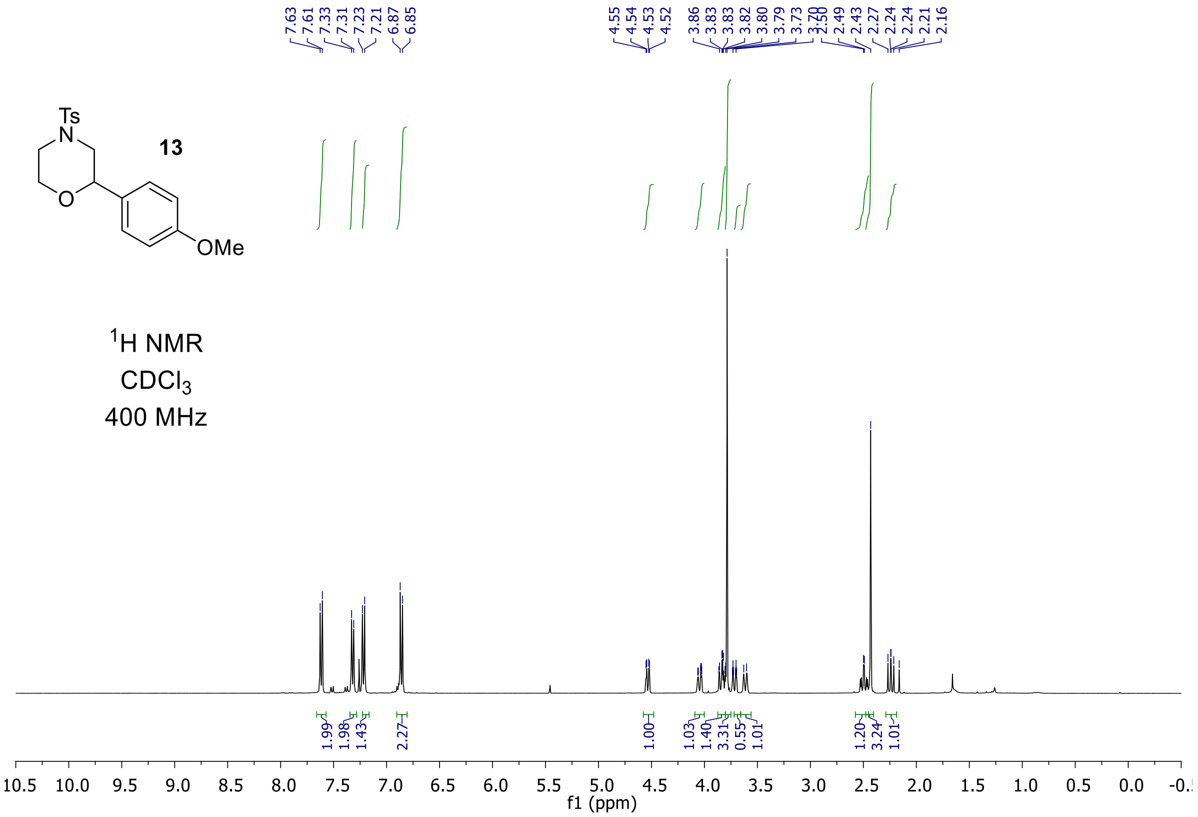
Ts
N
O
OMe
13
^{1}H NMR
CDCl$_3$
400 MHz
f1 (ppm)

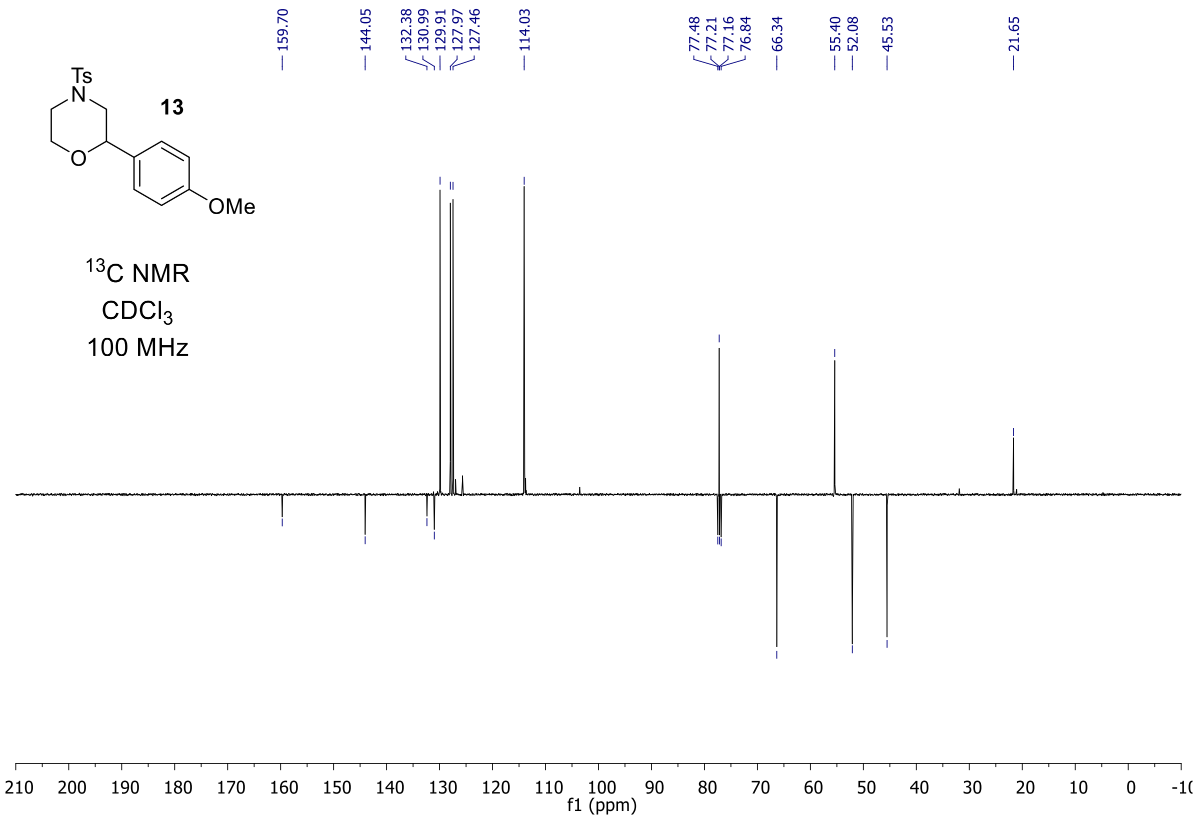
Ts
N
O
OMe
13
^{13}C NMR
CDCl$_3$
100 MHz
f1 (ppm)

HSQC (600 MHz, $CDCl_3$) **13**

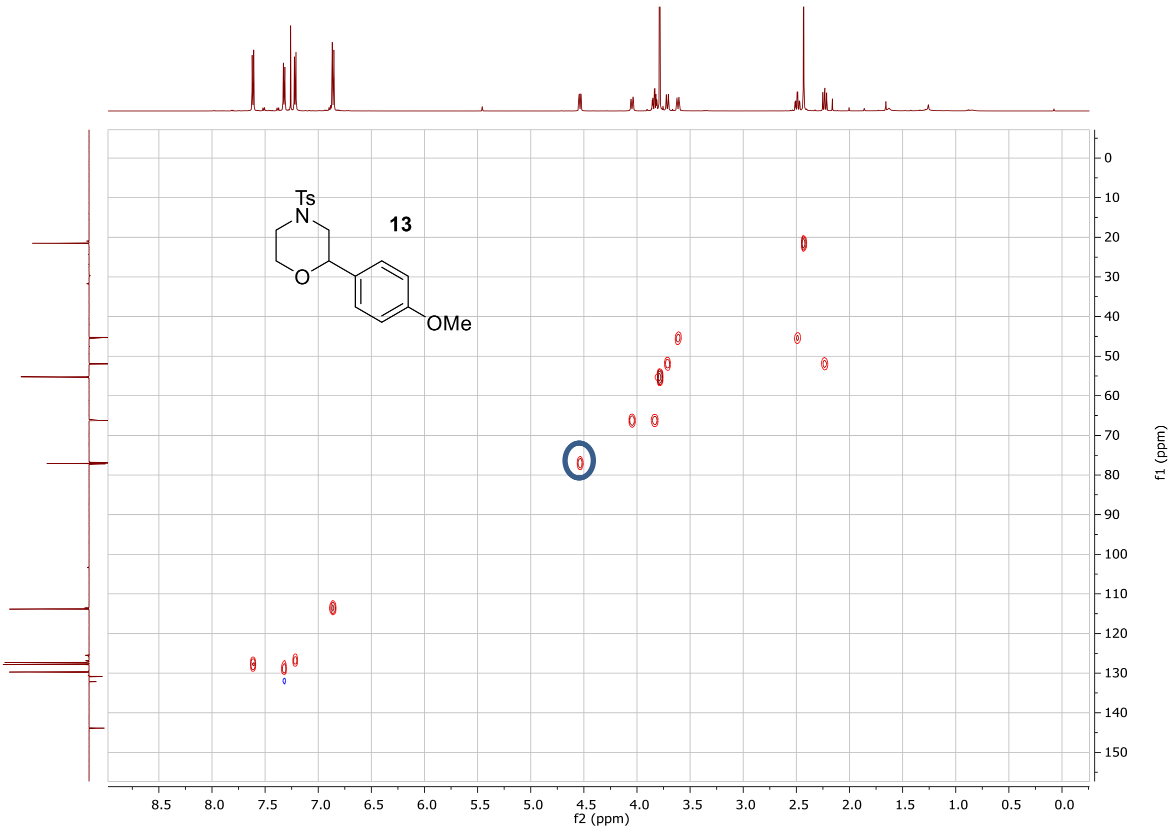

3 Computational details

Given the flexibility of the investigated molecules, wherever appropriate, the conformational space has been initially explored using the OPLS_2005 force field[6] and the systematic Monte Carlo conformers search routine implemented in MACROMODEL 11.5.[7]

Further, to consider the different possibilities that individual fragments can adopt within a particular complex, (e.g. the complex of a cation with the negatively charged TfO^- counterion), the electrostatic potential of the ions has been calculated applying a natural bond orbital (NBO) population analysis. The reciprocal positions of the fragments have been determined based on the calculated NBO charges. The so obtained complexes have been used to restrict further the conformational search and obtain the set of complexes that will be reoptimized using density functional theory (DFT) methods.

Accordingly, the structures located at force field level have then been subjected to a B3LYP-D3/def2-SVP[8–13] geometry optimization. The nature of all stationary points (minima and transition states) was verified through the computation of the vibrational frequencies. The thermal corrections to the Gibbs free energy were combined with the single point energies calculated at the DLPNO-CCSD(T)/def2-TZVP[14,15] to yield DLPNO-CCSD(T)//DFT Gibbs free energies ("G_{298}") at 298.15 K. All energies are reported in kcal mol^{-1}.

The density-based solvation model SMD[16] was applied to consider solvent (DCM) effects, both, in the geometries and energies. Solvation factors (for the Gibbs free energies at the DLPNO-CCSD(T)//DFT level of theory) have been calculated by single point energies in gas phase of the optimized geometries in solution. Free energies in solution have been corrected to a reference state of 1 mol l^{-1} at 298.15 K through addition of RTln(24.46) = +7.925 kJ mol^{-1} to the gas phase (1 atm) free energies.

The DFT calculations have been performed with the Gaussian09 program package.[17] The ORCA 4.0.1 software was applied for the DLPNO-CCSD(T) computations.[18]

3.1 Coordinates of the most stable ($\Delta G_{298,DCM}$) conformations as computed at the DLPNO-CCSD(T)/def2-TZVP//B3LYP-D3/def2-SVP level of theory

A

C	3.67415	1.46755	-0.53187
C	3.54222	1.05511	0.81774
C	2.30394	1.08733	1.41850
C	1.14721	1.52596	0.70685
C	1.30704	1.94850	-0.65372
C	2.53761	1.92758	-1.25253
H	4.40395	0.69951	1.38079
H	2.21748	0.75093	2.44980
O	4.81172	1.45924	-1.20249
C	6.02350	0.99037	-0.59799
H	6.79568	1.07029	-1.37305
H	6.29929	1.61779	0.26468
H	5.92345	-0.06071	-0.28447
C	-0.13229	1.44412	1.28497
C	-0.45491	0.94150	2.63887
H	0.43197	0.87276	3.27486
H	-1.20557	1.59129	3.11052
O	-1.11659	1.94970	0.53088
S	-2.76007	1.51058	0.47324
O	-3.10057	0.52701	1.47876
O	-3.42991	2.78769	0.35140
C	-2.77865	0.70188	-1.28229
F	-1.60411	0.78333	-1.86469
F	-3.17572	-0.54133	-1.15623
F	-3.66989	1.39633	-1.97279
H	-0.88630	-0.06607	2.53125
S	0.46280	-1.64966	0.24690
O	1.05732	-1.76044	1.59777
O	-0.81930	-0.88856	0.21249
O	1.39756	-1.34987	-0.85421
C	-0.11440	-3.39036	-0.12305
F	-0.98112	-3.81474	0.80440
F	0.92187	-4.23829	-0.14344
F	-0.72080	-3.44628	-1.31522
H	2.67418	2.23314	-2.29111
H	0.43595	2.26983	-1.22340

TS_{A-B}

C	-0.43260	-4.18335	0.52179
C	-1.09768	-3.39479	-0.44177
C	-0.46466	-2.28505	-0.96671
C	0.83770	-1.91014	-0.54908
C	1.51050	-2.73796	0.39850
C	0.88906	-3.85021	0.91878
H	2.51110	-2.47069	0.73983
H	1.38127	-4.48085	1.66137
O	-0.96653	-5.24909	1.11078
C	-2.31477	-5.62780	0.82150
H	-2.42755	-5.91251	-0.23725
H	-2.52834	-6.49578	1.45814
H	-3.01579	-4.81238	1.06326
C	1.40950	-0.68649	-1.00344
C	0.71999	0.40676	-1.59611
H	0.39784	1.12577	-0.65771
H	1.32827	1.04356	-2.25063
O	2.71922	-0.55911	-0.66150
S	3.63976	0.83690	-0.85433
O	4.11033	0.90132	-2.22252
O	3.01022	1.95307	-0.17627
C	5.01460	0.15863	0.25417
F	5.51991	-0.93144	-0.28756
F	5.93287	1.10258	0.34267
F	4.51017	-0.10847	1.44616
H	-0.25898	0.16277	-2.02003
S	-3.05463	-0.27458	-0.60381
O	-2.52874	0.15812	-1.92944
O	-3.81261	-1.54438	-0.63138
O	-2.11954	-0.11195	0.53040
C	-4.37850	1.00613	-0.25956
F	-5.33657	0.96239	-1.19368
F	-3.86256	2.24489	-0.25635
F	-4.94988	0.79575	0.93260
N	-0.15938	2.14486	0.42779
C	-0.89129	3.17893	-0.01036
C	-0.00039	1.88866	1.73496
C	-1.48980	4.05867	0.90216
C	-0.56955	2.73593	2.69376
C	-1.31871	3.83411	2.26772
H	-2.08895	4.89499	0.53713
H	-0.43284	2.52381	3.75601
H	-1.77645	4.50558	2.99897
C	-1.07484	3.30641	-1.49582
H	-1.62255	2.42482	-1.86666
H	-0.10184	3.33936	-2.01179
H	-1.64207	4.21113	-1.75390
C	0.76172	0.64855	2.10724
H	1.76724	0.65624	1.66239
H	0.86520	0.55185	3.19672
H	0.22163	-0.23099	1.72335
H	-0.99174	-1.69287	-1.71223
H	-2.11763	-3.61189	-0.75289

B(I)

C	-3.92095	0.39590	-0.08477
C	-2.82907	1.25036	-0.32022
C	-1.53681	0.74371	-0.38527
C	-1.28354	-0.63372	-0.21137
C	-2.38612	-1.47875	0.01572

C	-3.68666	-0.98232	0.08126
H	-3.01979	2.31761	-0.45128
H	-0.71434	1.43444	-0.57401
H	-2.23644	-2.55310	0.13904
H	-4.51006	-1.67435	0.25759
O	-5.13812	0.97629	-0.03861
C	-6.28580	0.17809	0.20753
H	-6.22488	-0.33528	1.18319
H	-7.14392	0.86388	0.21862
H	-6.43853	-0.57297	-0.58765
C	0.08783	-1.16875	-0.25899
C	0.53122	-2.39897	0.02296
H	-0.15305	-3.18347	0.34690
H	1.59221	-2.63761	-0.05837
S	1.92932	0.64870	0.35861
O	1.67788	0.21486	1.72238
O	1.04729	-0.21931	-0.70856
O	1.87224	2.04105	-0.05200
C	3.62273	0.00971	-0.15168
F	3.70074	-1.29017	0.10226
F	4.53773	0.65908	0.55335
F	3.80912	0.22741	-1.44323

B(II)

C	-3.54332	-0.71219	-0.21903
C	-2.51085	-1.47981	-0.75434
C	-1.26144	-0.87101	-0.91480
C	-1.03724	0.47121	-0.54764
C	-2.10825	1.22319	-0.00558
C	-3.34865	0.62971	0.15257
H	-2.67463	-2.52172	-1.03532
H	-0.44434	-1.46012	-1.33388
H	-1.95551	2.25952	0.30254
O	-4.80109	-1.14937	0.00350
C	-5.50170	-0.13120	0.76949
H	-6.52194	-0.05127	0.36962
H	-5.55615	-0.47792	1.81539
C	0.28680	1.08855	-0.73405
C	0.62138	2.38399	-0.75743
H	-0.14037	3.15953	-0.67118
H	1.66243	2.68553	-0.88053
S	1.98537	-0.71160	0.22102
O	1.33149	-0.42301	1.48653
O	1.33432	0.16321	-0.99758
O	2.19573	-2.05797	-0.27842
C	3.66823	0.12705	0.25662
F	3.51186	1.41838	0.51910
F	4.26768	-0.02423	-0.91339
F	4.39187	-0.43901	1.21215
C	-4.67362	1.16565	0.64182
H	-5.10950	1.85709	-0.09939
H	-4.60016	1.70490	1.59773

B(III)

C	4.44321	-0.73251	0.24963
C	4.20710	0.59137	0.64183
C	2.97305	1.19105	0.39336
C	1.94843	0.47620	-0.25886
C	2.19471	-0.85452	-0.64712
C	3.43205	-1.45158	-0.39328
H	4.98823	1.15839	1.15468
H	2.79966	2.21663	0.72516
H	1.41820	-1.42881	-1.15460
H	3.60387	-2.48559	-0.70280
C	0.64350	1.10971	-0.53585
C	0.31958	2.40657	-0.52493
H	1.07009	3.17054	-0.32087
H	-0.70193	2.72366	-0.73961
S	-1.13741	-0.72308	0.17023
O	-1.31440	-2.03777	-0.41762
O	-0.37283	0.20454	-0.94108
O	-0.58719	-0.50765	1.49791
C	-2.81007	0.13527	0.10406
F	-2.66430	1.41239	0.43283
F	-3.61570	-0.46082	0.97088
F	-3.30924	0.03886	-1.11736
H	5.40972	-1.20163	0.45056

TS$_{B\text{-}C(I)}$

C	3.56551	-0.36970	0.08896
C	3.37921	0.90872	-0.49688
C	2.20433	1.59470	-0.26621
C	1.18609	1.04068	0.56429
C	1.38198	-0.25845	1.13316
C	2.55059	-0.94182	0.90346
H	2.04468	2.57560	-0.71861
H	0.58885	-0.69872	1.73899
H	2.72690	-1.93234	1.32686
O	4.65382	-1.10720	-0.07121
C	5.73666	-0.65446	-0.89035
H	6.16856	0.27854	-0.49410
H	6.49178	-1.44969	-0.85755
H	5.40714	-0.50409	-1.93084
C	0.00854	1.72102	0.77480
C	-0.93519	2.47536	1.23719
H	-0.71704	3.08519	2.12541
O	-1.08727	0.73596	-1.00621
S	-2.50577	0.26665	-0.87050
O	-3.03275	-0.40594	-2.06545
O	-3.39798	1.23777	-0.19930

C	-2.34917	-1.11687	0.38810
F	-1.44820	-2.02273	-0.00917
F	-1.95208	-0.63751	1.57946
F	-3.52031	-1.73471	0.56201
H	-1.93929	2.50117	0.79318
H	4.14814	1.35285	-1.12779

TS$_{B-C(II)}$

C	-2.69682	-0.03125	0.73235
C	-2.53251	0.32720	-0.63157
C	-1.50968	1.16260	-0.99313
C	-0.62889	1.66066	0.02756
C	-0.80865	1.26098	1.39217
C	-1.84604	0.42619	1.75486
H	-1.34076	1.44714	-2.03335
H	-0.10147	1.62260	2.13743
H	-2.00657	0.11573	2.78819
O	-3.72513	-0.83627	0.93876
C	-4.44705	-1.04341	-0.32018
C	0.42728	2.46839	-0.30911
C	1.20212	3.48297	-0.53785
H	0.78603	4.49131	-0.40502
O	1.95154	0.88209	-0.97099
S	2.47525	-0.00981	0.11973
O	3.87629	-0.41738	-0.06266
O	2.06527	0.39051	1.47995
C	1.51427	-1.59089	-0.18046
F	1.83735	-2.51707	0.72813
F	0.19278	-1.37134	-0.09730
F	1.77465	-2.08553	-1.39522
H	2.24121	3.36687	-0.85498
C	-3.58384	-0.40466	-1.42875
H	-3.11950	-1.16825	-2.07272
H	-4.17065	0.26671	-2.07229
H	-4.59261	-2.12580	-0.43380
H	-5.42764	-0.55790	-0.20551

TS$_{B-C'}$

C	-3.22332	-1.46832	0.28154
C	-2.58729	-0.89975	1.39911
C	-1.93179	0.31131	1.27357
C	-1.91393	0.96734	-0.00019
C	-2.56464	0.37203	-1.12951
C	-3.21488	-0.83824	-0.97644
H	-1.40102	0.76541	2.10896
H	-2.52893	0.88112	-2.09425
H	-3.71272	-1.30888	-1.82621
C	-1.22377	2.13644	-0.15358
C	-0.53592	3.20704	-0.31760
H	0.52895	3.04480	-0.56956
O	1.67912	1.27974	-0.99342
S	1.90303	0.46828	0.23318
O	0.99564	0.79816	1.35846
O	3.31184	0.23178	0.59489
C	1.31481	-1.23231	-0.30503
F	2.10290	-1.73040	-1.26544
F	1.31942	-2.09080	0.72307
F	0.06088	-1.18480	-0.79023
H	-0.95476	4.21673	-0.21739
H	-2.60344	-1.41942	2.35890
H	-3.73379	-2.42891	0.39152

C(I)

C	1.67149	1.71748	0.17833
C	1.55802	0.94808	-1.01795
C	2.18454	-0.26287	-1.09849
C	2.99888	-0.74972	-0.00779
C	3.14210	0.07284	1.17616
C	2.49981	1.27165	1.25759
H	2.06406	-0.88802	-1.98441
H	3.76019	-0.28931	1.99982
H	2.57029	1.90610	2.14253
O	1.06370	2.85571	0.37119
C	0.09964	3.36274	-0.57232
H	-0.68420	2.61218	-0.74643
H	-0.32177	4.26067	-0.10503
H	0.59897	3.63438	-1.51527
C	3.60278	-1.95168	-0.09071
C	4.17533	-3.10798	-0.17064
H	5.18783	-3.21154	-0.58499
H	3.65786	-4.01412	0.17489
S	-1.31123	-0.26140	-0.22587
O	-0.85757	0.57617	0.91207
O	-1.53222	0.48265	-1.49229
O	-0.63221	-1.56703	-0.36702
C	-3.05049	-0.73840	0.28014
F	-3.04742	-1.39020	1.45105
F	-3.61258	-1.53915	-0.63587
F	-3.82735	0.34535	0.41365
H	0.90638	1.27526	-1.82429

C(II)

C	1.88412	1.40157	0.65025
C	1.76937	0.97455	-0.70602
C	2.04228	-0.31742	-1.03159
C	2.47012	-1.22592	0.01581
C	2.58885	-0.74912	1.37998
C	2.31164	0.55202	1.70076
H	1.92419	-0.69310	-2.04847
H	2.89877	-1.45687	2.15117

H	2.37778	0.93178	2.72091
O	1.55589	2.65020	0.82647
C	1.02153	3.21011	-0.42714
H	-0.03923	3.40910	-0.23213
H	1.56288	4.14888	-0.60471
C	2.71618	-2.51979	-0.26223
C	2.93836	-3.76551	-0.53038
H	3.93894	-4.11738	-0.81683
H	2.12264	-4.50065	-0.47886
S	-1.23947	-0.14103	-0.14479
O	-1.00619	1.16071	0.53191
O	-1.17607	-0.09630	-1.62489
O	-0.58140	-1.30466	0.48873
C	-3.05617	-0.45463	0.18615
F	-3.29926	-0.52789	1.50201
F	-3.45070	-1.60784	-0.37127
F	-3.81397	0.52805	-0.31972
C	1.23723	2.13184	-1.50640
H	0.29184	1.84232	-1.98653
H	1.95032	2.45559	-2.27990

C(III)

C	1.88882	-2.27647	-0.04561
C	1.93371	-1.59952	1.18663
C	2.29123	-0.26897	1.22095
C	2.63281	0.39893	-0.00499
C	2.59672	-0.31729	-1.25120
C	2.23450	-1.64666	-1.25578
H	2.30125	0.29496	2.15416
H	2.84178	0.21168	-2.17362
H	2.18373	-2.20327	-2.19305
C	2.91463	1.72592	0.00345
C	3.15767	2.99061	0.00795
H	4.18036	3.37585	0.12638
H	2.33082	3.70715	-0.10809
S	-0.98069	-0.17667	0.16675
O	-1.00378	-1.62331	-0.15468
O	-0.79120	0.15210	1.59809
O	-0.22199	0.67207	-0.78378
C	-2.75236	0.34954	-0.14566
F	-3.10379	0.13935	-1.42269
F	-2.91632	1.65502	0.11491
F	-3.60354	-0.33491	0.63180
H	1.64221	-2.11639	2.10180
H	1.57301	-3.32194	-0.06323

C'

C	-4.74832	-1.05025	0.09559
C	-4.88870	0.28915	0.47818
C	-3.81373	1.17037	0.35840
C	-2.58257	0.71212	-0.15505
C	-2.44683	-0.63785	-0.54303
C	-3.52771	-1.51005	-0.41285
H	-3.91685	2.21508	0.65962
H	-1.49320	-0.99120	-0.93888
H	-3.41647	-2.55539	-0.71140
C	-1.46930	1.60845	-0.27075
C	-0.50359	2.34509	-0.35581
H	0.33268	3.01726	-0.45970
O	0.93279	-0.70835	-1.27713
S	1.53258	-0.79899	0.04818
O	1.90625	-2.05964	0.65790
O	0.67199	0.01878	1.12112
C	3.07309	0.27422	0.00444
F	3.88875	-0.17986	-0.93624
F	2.72363	1.52721	-0.27579
F	3.67803	0.23342	1.18155
H	0.15016	0.74630	0.67437
H	-5.84131	0.64895	0.87488
H	-5.59225	-1.73776	0.19462

Lutidinium*OTf⁻

O	3.35571	0.33999	-1.11135
S	2.04471	0.62416	-0.51481
O	1.91514	1.86471	0.27467
O	0.88434	0.36011	-1.43349
C	1.81843	-0.72109	0.77264
F	2.76859	-0.65575	1.70690
F	0.62471	-0.58580	1.37867
F	1.85761	-1.93801	0.21531
N	-1.59534	0.14157	-0.45811
C	-2.19453	1.23302	0.07174
C	-2.14414	-1.09180	-0.52241
C	-3.48343	1.08738	0.58121
C	-3.43734	-1.25878	-0.02830
C	-4.10424	-0.16329	0.52436
H	-3.98767	1.95149	1.01529
H	-3.90515	-2.24300	-0.07288
H	-5.11651	-0.28524	0.91750
C	-1.41715	2.50927	0.06161
H	-0.40225	2.35007	0.45953
H	-1.29709	2.87193	-0.97336
H	-1.93146	3.28202	0.64703
C	-1.29969	-2.19756	-1.07210
H	-0.62919	-1.83450	-1.86408
H	-0.66266	-2.60432	-0.26855
H	-1.92717	-3.01198	-1.45770
H	-0.61605	0.26054	-0.83814

Lutidine

N	0.00795	-0.94545	-0.00240
C	-1.15527	-0.28226	0.00121
C	-1.20594	1.12433	0.00383
C	-0.01081	1.83867	0.00091
C	1.19941	1.13778	-0.00410
C	1.16811	-0.26307	-0.00455
C	2.42920	-1.08698	0.00256
H	2.41014	-1.82595	-0.81508
H	2.50801	-1.65833	0.94332
H	3.33058	-0.46572	-0.10115
H	2.15471	1.66755	-0.00704
H	-0.01651	2.93223	0.00177
H	-2.17086	1.63733	0.00682
C	-2.42483	-1.09546	-0.00077
H	-3.04959	-0.85991	0.87717
H	-2.18817	-2.16853	0.01159
H	-3.03310	-0.87857	-0.89517

TS$_{C'-D}$

C	-2.09820	3.44138	-1.55564
C	-0.78187	3.25672	-1.99969
C	0.23147	3.01365	-1.07895
C	-0.07779	2.95773	0.30377
C	-1.41366	3.14485	0.74472
C	-2.41335	3.38589	-0.18858
H	1.25650	2.82686	-1.40051
H	-1.64196	3.07074	1.80810
H	-3.44647	3.51894	0.13937
C	0.93303	2.63920	1.21527
C	1.85419	2.22952	1.95829
H	2.61382	2.65575	2.61543
O	1.65136	0.21252	-0.76354
S	2.58106	-0.56395	0.07141
O	2.79228	-1.98126	-0.21561
O	2.40092	-0.29456	1.56663
C	4.24254	0.23885	-0.25274
F	4.59090	0.06452	-1.52847
F	4.17917	1.55324	-0.00621
F	5.18384	-0.29343	0.52637
H	1.97759	0.96183	1.79494
H	-0.55294	3.28985	-3.06708
H	-2.89341	3.62108	-2.28381
O	-0.86608	0.26351	1.52565
S	-1.25620	-0.09792	0.09767
C	-0.72722	-1.82567	-0.14116
C	-0.30079	-2.54685	0.97243
C	-0.69046	-2.36660	-1.42833
C	0.14037	-3.86124	0.79525
H	-0.30080	-2.06615	1.95270
C	-0.25153	-3.68306	-1.59278
H	-0.99255	-1.77045	-2.29348
C	0.16031	-4.43025	-0.48227
H	0.48060	-4.43944	1.65834
H	-0.21919	-4.12286	-2.59301
H	0.51106	-5.45678	-0.61705
C	-3.06030	-0.39215	0.14701
C	-3.69383	-0.44776	1.38885
C	-3.78229	-0.46993	-1.04781
C	-5.08201	-0.61299	1.43461
H	-3.09439	-0.35390	2.29753
C	-5.16941	-0.63882	-0.99109
H	-3.27333	-0.39844	-2.01311
C	-5.81839	-0.70990	0.24794
H	-5.59085	-0.66249	2.40103
H	-5.74571	-0.70535	-1.91756
H	-6.90356	-0.83511	0.28806

D

C	6.39295	-0.96016	-0.99031
C	5.77243	0.23447	-1.36850
C	4.37896	0.31215	-1.43355
C	3.58601	-0.80849	-1.12122
C	4.22005	-2.00449	-0.73103
C	5.61165	-2.07886	-0.67249
H	6.09098	-3.01191	-0.36525
H	3.61676	-2.87153	-0.45331
C	2.11233	-0.71977	-1.18391
C	1.22722	-1.71064	-1.35808
H	0.15330	-1.50974	-1.31709
H	1.57406	-2.72241	-1.57617
O	1.58217	0.57363	-1.07006
S	1.71056	1.32376	0.45875
C	1.11317	0.07683	1.56622
C	2.11085	-0.61189	2.27894
C	1.73014	-1.67920	3.09280
C	0.38222	-2.05064	3.17702
C	-0.59658	-1.35268	2.45808
C	-0.24036	-0.27684	1.64748
H	-1.02002	0.25461	1.10681
H	-1.64853	-1.64168	2.50743
H	0.09249	-2.89300	3.81020
H	2.48958	-2.22473	3.65721
H	3.16186	-0.32526	2.19557
C	0.39523	2.50012	0.22734
C	0.53295	3.71988	0.90385
C	-0.50334	4.65023	0.79981
C	-1.63895	4.35723	0.03451
C	-1.74615	3.13806	-0.64431
C	-0.71895	2.19760	-0.56305
H	-0.79637	1.24518	-1.08567

H	-2.63823	2.88650	-1.22180
H	-2.44865	5.08803	-0.03308
H	-0.42048	5.60732	1.31961
H	1.42058	3.94073	1.50178
H	3.89997	1.24311	-1.74370
H	6.37509	1.11109	-1.61945
H	7.48308	-1.02003	-0.93769
O	-3.19103	0.30265	0.74802
S	-3.20672	-0.22073	-0.64008
O	-1.91385	-0.78870	-1.10520
O	-3.91300	0.62113	-1.62697
C	-4.28352	-1.74391	-0.49244
F	-5.51673	-1.42120	-0.08464
F	-4.38522	-2.37800	-1.66658
F	-3.76374	-2.60175	0.39862

Ph_2SO

S	0.00428	1.25212	1.01145
O	0.03266	2.56050	0.24071
C	-1.37505	0.27001	0.31224
C	-1.93585	0.69506	-0.89223
C	-1.87154	-0.83660	1.00657
C	-3.00311	-0.02862	-1.43254
H	-1.53366	1.58414	-1.38408
C	-2.93773	-1.55492	0.45478
H	-1.43850	-1.13829	1.96421
C	-3.50051	-1.15320	-0.76275
H	-3.44976	0.28883	-2.37849
H	-3.33571	-2.42537	0.98267
H	-4.33669	-1.71469	-1.18736
C	1.36888	0.23213	0.32957
C	2.09557	0.75465	-0.73951
C	1.69574	-0.99617	0.91160
C	3.16259	0.01292	-1.25861
H	1.81762	1.72935	-1.14760
C	2.76199	-1.72970	0.38281
H	1.12689	-1.38317	1.76130
C	3.49354	-1.22701	-0.70103
H	3.73812	0.40751	-2.10014
H	3.02611	-2.69472	0.82296
H	4.32877	-1.80283	-1.10785

4 References

[1] J. H. Chun, C. L. Morse, F. T. Chin, V. W. Pike, *Chem. Commun.* **2013**, *49*, 2151–2153.

[2] X. Li, Y. Sun, X. Huang, L. Zhang, L. Kong, B. Peng, *Org. Lett.* **2017**, *19*, 838–841.

[3] D. Kaiser, L. F. Veiros, N. Maulide, *Chem. - A Eur. J.* **2016**, *22*, 4727–4732.

[4] K. R. Law, C. S. P. McErlean, *Chem. - A Eur. J.* **2013**, *19*, 15852–15855.

[5] J. V. Matlock, T. D. Svejstrup, P. Songara, S. Overington, E. M. McGarrigle, V. K. Aggarwal, *Org. Lett.* **2015**, *17*, 5044–5047.

[6] J. L. Banks, H. S. Beard, Y. Cao, A. E. Cho, W. Damm, R. Farid, A. K. Felts, T. A. Halgren, D. T. Mainz, J. R. Maple, et al., *J. Comput. Chem.* **2005**, *26*, 1752–1780.

[7] "MacroModel, Schrödinger, LLC, New York, NY, 2018.," **2018**.

[8] A. Becke, *J. Chem. Phys.* **1993**, *98*, 5648–5652.

[9] C. Lee, W. Yang, R. G. Parr, *Phys. Rev. B* **1988**, *37*, 785–789.

[10] S. H. Vosko, L. Wilk, M. Nusair, *Can. J. Phys.* **1980**, *58*, 1200–1211.

[11] P. J. Stephens, F. J. Devlin, C. F. Chabalowski, M. J. Frisch, *J. Phys. Chem.* **1994**, *98*, 11623–11627.

[12] S. Grimme, J. Antony, S. Ehrlich, H. Krieg, *J. Chem. Phys.* **2010**, *132*, 154104.

[13] F. Weigend, R. Ahlrichs, *Phys. Chem. Chem. Phys.* **2005**, *7*, 3297–305.

[14] C. Riplinger, F. Neese, *J. Chem. Phys.* **2013**, *138*, 034106.

[15] C. Riplinger, B. Sandhoefer, A. Hansen, F. Neese, *J. Chem. Phys.* **2013**, *139*, 134101.

[16] A. V. Marenich, C. J. Cramer, D. G. Truhlar, *J. Phys. Chem. B* **2009**, *113*, 6378–6396.

[17] Frisch, G. Trucks, H. Schlegel, G. Scuseria, M. Robb, J. Cheeseman, G. Scalmani, V. Barone, B. Mennucci, G. Petersson, et al., *Gaussian 09, Revis. D.01, Gaussian, Inc., Wallingford CT* **2013**.

[18] F. Neese, *Wiley Interdiscip. Rev. Comput. Mol. Sci.* **2012**, *2*, 73–78.
